# Secreted metalloproteases ADAMTS9 and ADAMTS20 have a non-canonical role in ciliary vesicle growth during ciliogenesis

**DOI:** 10.1038/s41467-019-08520-7

**Published:** 2019-02-27

**Authors:** Sumeda Nandadasa, Caroline M. Kraft, Lauren W. Wang, Anna O’Donnell, Rushabh Patel, Heon Yung Gee, Kay Grobe, Timothy C. Cox, Friedhelm Hildebrandt, Suneel S. Apte

**Affiliations:** 1 Department of Biomedical Engineering- ND20, Cleveland Clinic Lerner Research Institute, Cleveland, OH 44195 USA; 2 Department of Pharmacology, Yonsei University College of Medicine, 50-1 Yonsei-ro, Seoul, 03722 South Korea; 3 Institute of Physiological Chemistry and Pathobiochemistry and Cells-in-Motion Cluster of Excellence (EXC1003-CiM), University of Münster, 48149 Münster, Germany; 4 Division of Craniofacial Medicine, Department of Pediatrics, University of Washington, 1959 NE Pacific St, Seattle, WA 98195 USA; 5 Department of Oral and Craniofacial Sciences, UMKC School of Dentistry, 650 E 25th St, Kansas City, MO 64108 USA; 6 Division of Nephrology, Boston Children’s Hospital, Harvard Medical School, 300 Longwood Avenue, Boston, MA 02115 USA

## Abstract

Although hundreds of cytosolic or transmembrane molecules form the primary cilium, few secreted molecules are known to contribute to ciliogenesis. Here, homologous secreted metalloproteases ADAMTS9 and ADAMTS20 are identified as ciliogenesis regulators that act intracellularly. Secreted and furin-processed ADAMTS9 bound heparan sulfate and was internalized by LRP1, LRP2 and clathrin-mediated endocytosis to be gathered in Rab11 vesicles with a unique periciliary localization defined by super-resolution microscopy. CRISPR-Cas9 inactivation of *ADAMTS9* impaired ciliogenesis in RPE-1 cells, which was restored by catalytically active ADAMTS9 or ADAMTS20 acting in *trans*, but not by their proteolytically inactive mutants. Their mutagenesis in mice impaired neural and yolk sac ciliogenesis, leading to morphogenetic anomalies resulting from impaired hedgehog signaling, which is transduced by primary cilia. In addition to their cognate extracellular proteolytic activity, ADAMTS9 and ADAMTS20 thus have an additional proteolytic role intracellularly, revealing an unexpected regulatory dimension in ciliogenesis.

## Abstract (web summary)

Ciliogenesis is a complex process requiring hundreds of molecules, although few secreted proteins have been implicated. Here, the authors show that the secreted metalloproteases ADAMTS9 and ADAMTS20 intracellularly regulate ciliogenesis from unique periciliary vesicles with proteolytic activity.

## Introduction

Proteolysis is an irreversible posttranslational modification with profound biological impact. Secreted and cell-surface proteases proteolytically modify extracellular matrix (ECM) and other secreted molecules, and a few also act intracellularly^1,2^ but none are known to participate in the biogenesis of cellular organelles. The primary cilium, which is solitary and sessile, compared to the motile cilia of ciliated epithelium, is a unique cellular organelle that is intimately associated with the extracellular environment and found in most post-mitotic cells^3^. It has a microtubule core and is enriched in several hundred other components^4^. The primary cilium extends by microtubule growth from a basal body, which is the post-mitotic maternal centrosome, to form a ciliary axoneme and it has a role in transduction of a variety of signals, including of morphogens such as hedgehogs^5^. Molecules localizing to the ciliary compartment and the peri-centrosomal vesicles are tightly regulated by the cell, trafficking via recycling endocytic or secretory vesicles and gaining privileged entry to the cilium via a proximal axoneme transition zone, a “gatekeeper” at the base of the cilium^6^. Transmembrane components such as smoothened are targeted to the ciliary membrane not only by lateral transport through plasma membrane but also via a complex mechanism utilizing cilium-bound recycling endocytic vesicles from the recycling endosome^7,8^. Diverse gene mutations result in primary cilium malformation or dysfunction, and the resulting clinical disorders, termed ciliopathies, affect the development of the kidney, heart, eye, brain, as well as metabolism^9^. Several signaling pathways additional to hedgehog signaling are mediated by the primary cilium, including TGFβ and PDGFRα^10^. Here we have uncovered the existence of a metalloproteinase-rich vesicular compartment at the base of the cilium, defining its origins and functional impact and providing role for proteolysis in biogenesis of the cilium.

Among a large family of secreted proteases having a characteristic domain structure, ADAMTS9 and ADAMTS20 are highly conserved orthologs of the nematode protease Gon-1, which is essential for gonadogenesis^11,12^. *Adamts9*-null embryos die at 7 days of gestation without undergoing gastrulation. In contrast, spontaneous *Adamts20*^Bt^ mutants survive and have defined a role for ADAMTS20 in remodeling of dermal ECM that regulates melanocyte colonization of skin^13,14^. Canine and human *ADAMTS20* variants are associated with cleft palate, cleft lip and syndactyly^15^. *Adamts20*^Bt/Bt^ mice have a low incidence of cleft palate, but upon deletion of one *Adamts9* allele, they have fully penetrant cleft palate and further reduction in pigmented hair follicles^16,17^. ADAMTS9 and ADAMTS20 participate in regression of interdigital webs via cleavage of the proteoglycan versican, a major component of the embryonic ECM^18^ and versican accumulates in anomalies resulting from ADAMTS9 or ADAMTS20 inactivation^16–18^, identifying it as a key ADAMTS9 and ADAMTS20 substrate.

Here, we show that combined inactivation of both ADAMTS9 and ADAMTS20 impairs formation of primary cilia and causes severe developmental anomalies, which include craniofacial malformations and neural tube defects. These findings provide unexpected insights on ciliogenesis and a non-canonical intracellular role for proteases hitherto thought to have exclusively extracellular actions.

## Results

### ADAMTS9 and ADAMTS20 are enriched at the primary cilium base

**Fig. 1:**
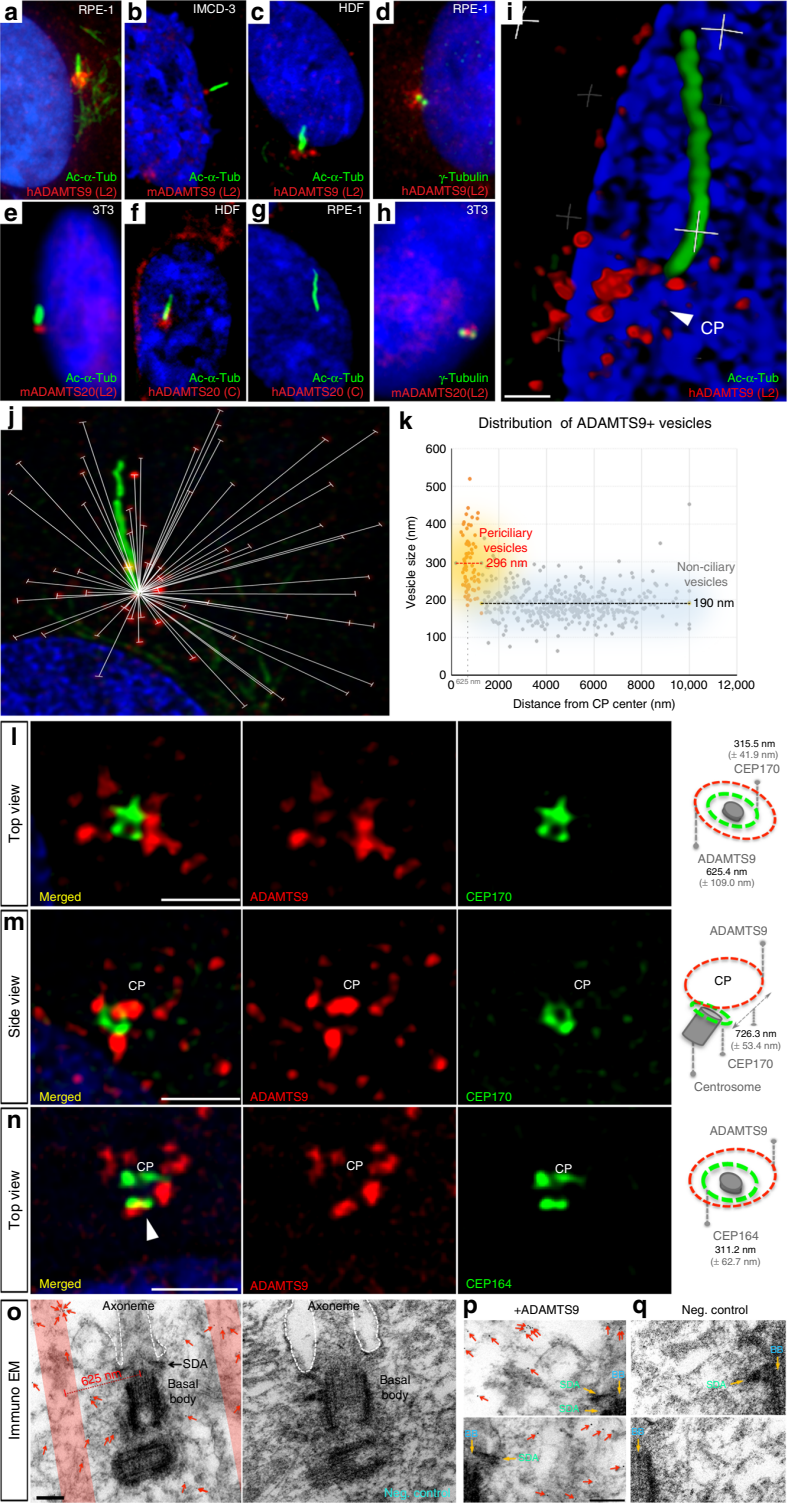
ADAMTS9 and ADAMTS20 localize to the cilium base. **a**–**c** Immunostaining for primary cilia (acetylated α-tubulin, green), and human or mouse ADAMTS9 (red), shows ADAMTS9 localization at the primary cilium base in serum-starved RPE-1 cells (**a**), IMCD-3 cells (**b**), and human dermal fibroblasts (HDFs) (**c**). **d** Co-immunostaining of γ-tubulin (green) shows ADAMTS9 (red) around the basal body. **e**–**g** Focal ADAMTS20 staining (red) is present at the base of the primary cilium of NIH-3T3 cells (**e**), and HDFs (**f**), but not RPE-1 cells (**g**). **h** ADAMTS20 (red) is adjacent to the basal body (γ-tubulin, green) of NIH-3T3 cells. **i** 3D-projection of deconvolution super-resolution confocal microscopy (DSCM) of RPE-1 cells (imaged at ×1000 magnification) shows vesicle-like ADAMTS9 staining (red) at the primary cilium base (CP, presumed ciliary pocket, white arrowhead). **j**, **k** Representative DSCM image used for determining size and geographic distribution of ADAMTS9+ vesicles (**j**) and scatter plot (**k**) summarizing the analysis. **l**, **m** ADAMTS9+ vesicles (red) are located lateral and distal (above) to CEP170 (green), which defines the lateral-most boundary of sub-distal appendages (315.5 nm ± 41.9 nm SD). **n** ADAMTS9+ vesicles (red) are located lateral to the distal appendages (CEP164 (green)) (311.2 nm ± 62.7 nm SD). The arrowhead shows apparent overlap that was nonoverlapping in 3D. Right-hand panels depict relative localizations and radial distances measured by DSCM. **o** Immuno-EM of ADAMTS9 in RPE-1 cells shows 5 nm gold localization (arrowheads) in a barrel-shaped zone (pink shading) surrounding the basal body and 625 nm ± 110 nm from its center. Secondary antibody-only negative control lacks gold particles. **p**, **q** High magnification images show 5 nm gold particles (red arrows) localized to the left (top image) or right side (bottom image) of the basal body in the ADAMTS9-stained TEM samples, whereas none are visible in the negative control. Yellow arrows point to the basal body and the sub-distal appendages. Scale bars = 500 nm in **i**, 2 μm in **l**–**n** and 200 nm in **o**–**p**

ADAMTS9 and ADAMTS20 are secreted proteases known to proteolytically cleave the ECM component versican. Surprisingly, several new ADAMTS9- and ADAMTS20 mono-specific polyclonal antibodies we generated (Supplementary Fig. 1a–c) as well as commercial antibodies showed intense staining of ADAMTS9 and ADAMTS20 at the base of the primary cilium in human RPE-1 cells, mouse IMCD-3 cells, mouse NIH-3T3 cells, and primary human dermal fibroblasts (HDFs) upon induction of ciliogenesis by 24 h of serum starvation (Fig. 1a–h). ADAMTS9 localized to the cilium base in each cell type, and surrounded the γ-tubulin-stained basal body (Fig. 1d). ADAMTS20 antibodies similarly stained the base of the primary cilium of HDFs and NIH-3T3 cells respectively, but not RPE-1 cells, which do not express *ADAMTS20* (Fig. 1e–g). Deconvolution super-resolution confocal microscopy (DSCM), with a defined resolution of 140 nm, consistently resolved ADAMTS9 localization to multiple well-circumscribed vesicular structures forming rosette-like patterns at the base of primary cilia (Fig. 1l, Supplementary Fig. 2a). Spatial mapping of ADAMTS9+ vesicles using DSCM revealed distinct vesicle populations, one comprising relatively small vesicles (average diameter 190 nm) distributed extensively across the cell surface and not vicinal to the centrosome, whereas significantly larger (average diameter 296 nm) vesicles were located in a barrel-shaped distribution 625.4 ± 109.0 nm from the centrosomal axis (Fig. 1j, k). To define their precise spatial relationship to the basal body and the cell membrane, we localized ADAMTS9 and centrosome appendage-specific markers by DSCM. ADAMTS9-stained vesicular rosettes were further lateral to and nearer the cell surface than the outermost boundary of the centrosome defined by CEP170, a sub-distal appendage (SDA) marker^19^ (Fig. 1l, m). ADAMTS9 showed minimal overlap with the distal appendage marker CEP164 ^19^, being located further lateral to it, but at a similar distance from the cell membrane (Fig. 1n). Immunogold electron microscopy revealed intracellular gold particles labeling ADAMTS9 that surrounded the basal body (625.4 ± 109.0 nm from its axis (Fig. 1o–q)) consistent with DSCM. The pre-embedding immunostaining method used precluded the observation of membranous vesicles due to the detergents used to permeabilize cells.

### Secreted ADAMTS9 undergoes receptor-mediated endocytosis

**Fig. 2:**
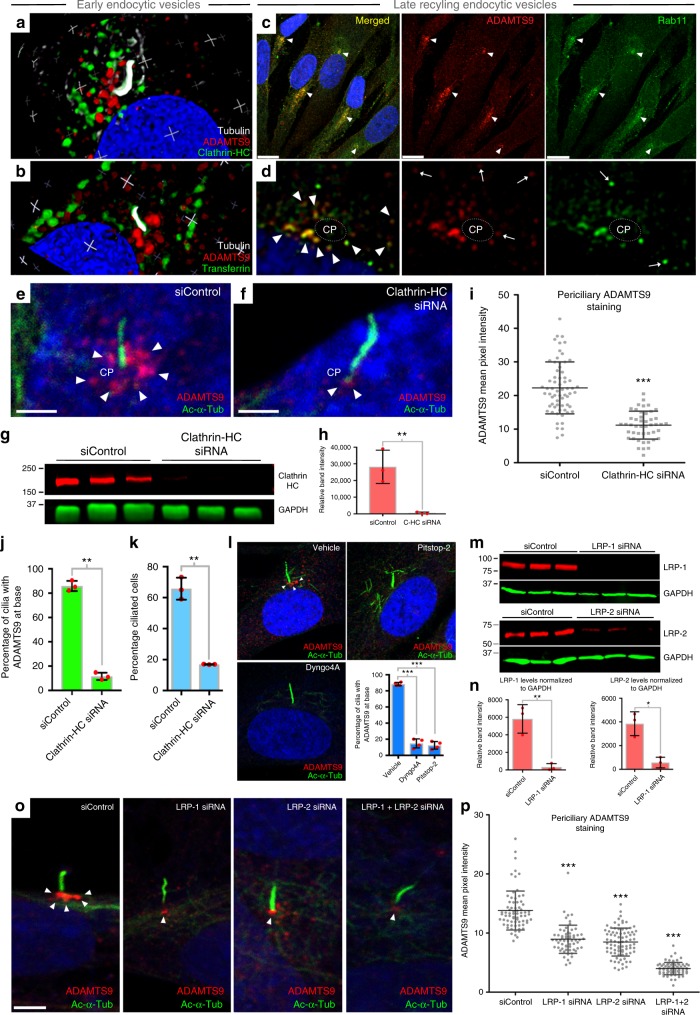
ADAMTS9 is internalized by clathrin-mediated endocytosis and transported to the periciliary region. **a**, **b** 3D-projections of DSCM images of **a** ADAMTS9 (red), clathrin heavy chain (HC) (green), and tubulin (white), in RPE-1 cells show clathrin-coated endocytic vesicles surrounding the primary cilium but no overlap with ADAMTS9+ vesicles. **b** Endocytic vesicles (Alexa Fluor 647-transferrin, green) show minimal overlap with ADAMTS9+ vesicles at the cilium base. **c**, **d** Low magnification (**c**) and DSCM images (**d**) of ADAMTS9 (red) and Rab11 (green) show co-staining in RPE-1 cells. Arrowheads in **d** show co-stained vesicles surrounding the ciliary pocket (CP), and white arrows point to non-co-stained vesicles. **e**, **f** Clathrin-HC knockdown results in a loss of ADAMTS9 staining at the cilium base (*N* = 3 independent experiments). **g**, **h** Western blot and clathrin-HC (red) quantification in RPE-1 cell lysates 48 h after siRNA transfection normalized to GAPDH (green) as control (*N* = 3 independent experiments, **p < 0.001, Student’s *t* test). **i** Quantification of ADAMTS9 pixel intensity at the cilium base shows significant reduction by clathrin-HC siRNA (*N* = 3 independent experiments, ***p < 0.0001, Student’s *t* test). **j**, **k** Percentage of ciliated cells and percentage of cilia with ADAMTS9 staining (*N* = 3 independent experiments, **p < 0.001; ***p < 0.0001, Student’s *t* test). **l** Treatment of RPE-1 cells with the clathrin inhibitors Dyngo4A or Pitstop-2 after serum starvation for 24 h results in loss of ciliary ADAMTS9 staining (*N* = 4 independent experiments, ***p < 0.0001, Student’s *t* test). **m**, **n** Western blot and quantification of LRP-1 and LRP-2 48 h after siRNA treatment shows depletion of LRP-1 and LRP-2 (normalized to GAPDH, green) (*N* = 3 independent experiments for each treatment, **p < 0.001, *p < 0.05, Student’s *t* test). **o** Immunostaining of RPE-1 cells with ADAMTS9 (red) and acetylated α-tubulin (green), after serum starvation of LRP-1- and LRP-2-depleted cells or with combined depletion shows reduced ciliary ADAMTS9. **p** ADAMTS9 pixel intensity at the cilium base after treatment of LRP-1, LRP-2 or LRP-1 + LRP-2 siRNAs is significantly reduced compared to control siRNA levels (*N* = 3 independent experiments, ***p < 0.0001, Student’s *t* test). Scale bars = 10 μm in **c**, 2 μm in **e**, **f** and **o**. Bar charts and dotplots show mean and SD (error bars)

An ADAMTS9 propeptide antibody gave no staining at the base of the cilium, despite recognition of ADAMTS9 zymogen on western blots (Supplementary Fig. 2b,c), indicating that cilium-associated ADAMTS9 lacked the propeptide. Indeed, it was previously established that the ADAMTS9 propeptide was excised extracellularly by furin^20^, suggesting the interesting possibility that ADAMTS9-positive vesicle-like structures at the base of the cilium contained secreted, furin-processed and endocytosed ADAMTS9. Antibodies to clathrin heavy chain (CHC) or endocytosed Alexa 647 fluor-labeled transferrin mark early endocytic vesicles surrounding the cilium^21,22^ (Fig. 2a, b), but ADAMTS9 staining overlapped minimally with such vesicles. In contrast, costaining with Rab11, a post-recycling endocytic vesicle marker, showed considerable overlap with ADAMTS9 at the cilium base (Fig. 2c, d). Rab11+ and Rabin8+ recycling endocytic vesicles contribute to the ciliary vesicle^23^, whose growth at the distal end of the basal body, thus transporting cargo essential for the growing cilium, is a necessary process in ciliogenesis^24–26^. To test if ADAMTS9 localization to the cilium required endocytosis, we depleted CHC mRNA (Fig. 2e–h), which significantly reduced ADAMTS9+ vesicles at the cilium base (Fig. 2e, f, i, j), as well as ciliogenesis in RPE-1 cells (Fig. 2k). Inhibition of clathrin-mediated endocytosis starting 24 h after serum starvation (and thus following cilium formation) using the clathrin inhibitors Dyngo4A or Pitstop2 eliminated ADAMTS9 localization at the cilium (Fig. 2l). Dyngo4A reduced cilium length, but neither inhibitor affected the percentage of ciliated cells (Supplementary Fig. 3b,c). This finding suggested that ADAMTS9 was essential for ciliogenesis, and likely to be necessary for maintenance of cilium length. Taken together, the results suggested that secreted ADAMTS9 was recycled, localizing to a subpopulation of late recycling endocytic vesicles targeted to the primary cilium in a highly dynamic process necessary for ciliogenesis.

A yeast-two hybrid screen for ADAMTS9 interactors using its Gon1-domain identified low-density lipoprotein receptor-related protein-1 (LRP1), which mediates receptor-mediated endocytosis of several metalloproteinases, including ADAMTS4 and ADAMTS5^27^. Previous work demonstrated that LRP3 was essential for ciliogenesis in RPE-1 cells^7^ and LRP2 for neural tube morphogenesis and Shh signaling in the mouse retina^28^. LRP-1 and LRP-2 knockdown individually or in combination followed by serum starvation significantly reduced ADAMTS9+ vesicles at the cilium base (Fig. 2m–p). These results suggested that LRP-1 and LRP-2 and clathrin-mediated endocytosis were necessary for ADAMTS9 recycling.

### ADAMTS9 and ADAMTS20 act as proteases in ciliogenesis

**Fig. 3:**
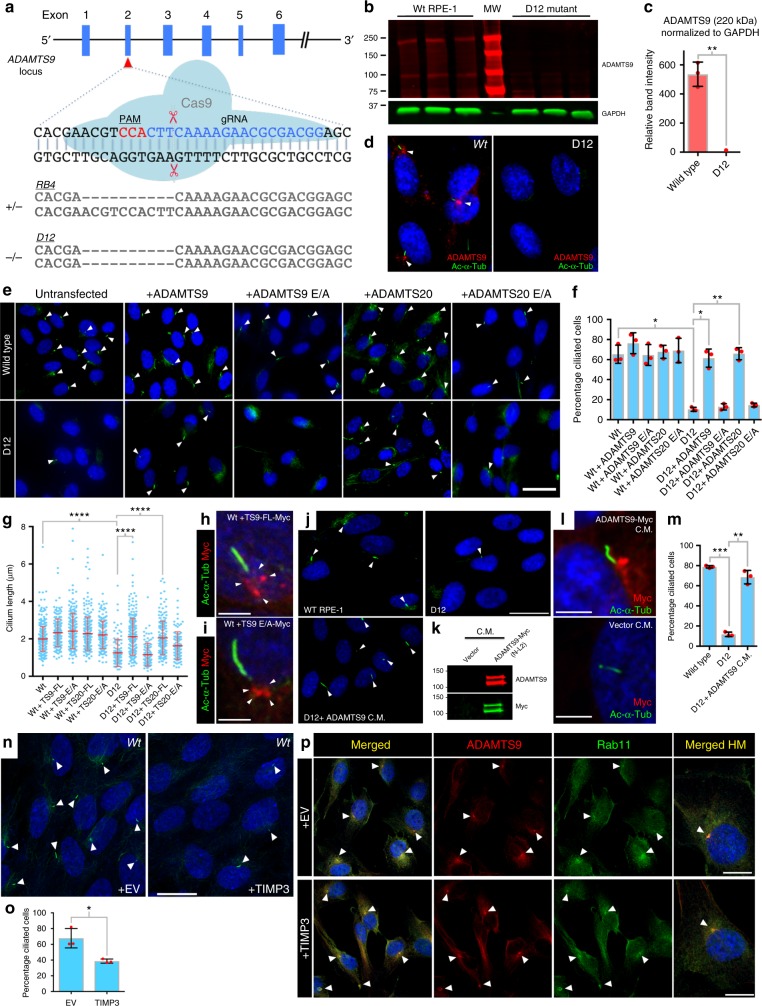
ADAMTS9 and ADAMTS20 catalytic activity is essential and functionally redundant in ciliogenesis. **a** CRISPR-Cas9 inactivation of *ADAMTS9* in RPE-1 cells and sense-strand sequence of specific indels obtained in hemizygous (RB4) and homozygous (D12) RPE-1 clones. The PAM site and guide RNA (gRNA) sequences are shown in red and blue letters, respectively. **b**–**d** Western blotting (**b**, **c**) and immunofluorescence (**d**) shows elimination of ADAMTS9 in D12 cells. Arrowheads point to ADAMTS9 rich foci at the base of cilia. For (**c**), *N* = 3 lanes each group, **p < 0.001. **e−g** Immunofluorescence of wild-type RPE-1 and D12 cells with acetylated α-tubulin (green) after transfection with the indicated constructs (**e**) and the percentage of ciliated cells (**f**) and cilium length (**g**) resulting. Note presence of the primary cilium in D12 cells transfected with either wild-type ADAMTS9 or ADAMTS20, but not in the corresponding catalytically inactive (E/A) mutants (*N* = 3 independent experiments, ****p < 0.00001; **p < 0.001; *p < 0.05, Student’s *t* test). Arrowheads point to primary cilia. **h**, **i** Transfected myc-tagged (red) ADAMTS9 (**h**) and ADAMTS9-E/A (**i**) localize to the base of the primary cilium (green) in wild-type RPE-1 cells. Arrowheads point to vesicles at the base of the primary cilium. **j** ADAMTS9-containing conditioned medium (C.M.) restores ciliogenesis in D12 cells. Arrowheads point to primary cilia. **k** Western blot of supplemented medium with anti-myc (green) or anti-ADAMTS9 (red). **l** Myc-tag (red) immunostaining shows concentration of exogenously supplied ADAMTS9 at the base of the cilium. **m** Quantitation of the percentage of ciliated cells in wild-type, D12 and D12 cells treated with exogenous ADAMTS9 shows rescue of ciliogenesis in D12 cells (*N* = 3 independent experiments, ***p < 0.0001, **p < 0.001, Student’s *t* test). **n**, **o** Transfection of the metalloprotease inhibitor TIMP3 significantly reduces the percentage of ciliated RPE-1 cells (*N* = 3 independent experiments, *p < 0.05, Student’s *t* test). **p** Transfection of the metalloprotease inhibitor TIMP3 does not affect ADAMTS9 (red) and Rab11 (green) colocalization. Arrowheads point to ADAMTS9/Rab11 rich foci in cells. Scale bars = 25 μm in **e**, 20 μm in **j**, **n**, 10 μm in **p**, 3 μm in **h**, **i**, **l**. Bar charts and dotplots show mean and S.D (error bars)

RPE1 cells express ADAMTS9 but not ADAMTS20 (Fig. 1a, g). CRISPR/Cas-9 targeting of exon 2 of *ADAMTS9* (Fig. 3a) generated sequence-verified RPE-1 clones with heterozygous (RB4) and homozygous (D12) frameshift indels (Fig. 3a) without off-target mutations (Supplementary Fig. 3f). Loss of ADAMTS9 immunostaining and western blotting of mutant cells validated *ADAMTS9* inactivation (Fig. 3b–d) (Supplementary Fig. 3g–j). Serum-starved RB4 and D12 cells each showed significantly reduced ciliogenesis (Fig. 3e, f; Supplementary Fig. 3j,k) and significantly shorter primary cilia (Fig. 3g), indicating that in the absence of ADAMTS20, RPE-1 cells were highly sensitive to reduced ADAMTS9. Overexpression of ADAMTS9 and ADAMTS20 in RB4 and D12 cells restored ciliogenesis, but importantly, their catalytically inactive mutants did not (Fig. 3e–g), (Supplementary Fig. 3j,k). The C-terminal myc tag of transfected wild type or catalytically inactive ADAMTS9 localized peri-centrosomally (Fig. 3h, i). This finding demonstrated not only that ADAMTS9 acted via the secretory pathway (i.e., was secreted and subsequently internalized), but argued against a putative alternatively spliced gene product that could regulate ciliogenesis by its targeting to the cytosol. The periciliary localization of the catalytically inactive form also eliminated the possibility that it failed to rescue ciliogenesis owing to defective trafficking to the cilium base. Most significantly, serum-free medium from myc-tagged ADAMTS9-expressing HEK293T cells rescued the ciliogenesis defect of D12 cells, and myc-tag staining was observed at the cilium base, supporting trafficking by endocytic recycling and action in-*trans* (Fig. 3j–m). Consistent with a requirement for catalytic activity, overexpression of the metalloprotease inhibitor TIMP3, which inhibits ADAMTS proteases^29^, suppressed ciliogenesis (Fig. 3n, o). Overexpression of TIMP3 did not impair ADAMTS9 trafficking to Rab11+ vesicles ruling out altered trafficking of ADAMTS9 by TIMP3 and supporting blockade of proteolytic activity (Fig. 3p).

### ADAMTS9 knockout arrests specific aspects of ciliogenesis

**Fig. 4:**
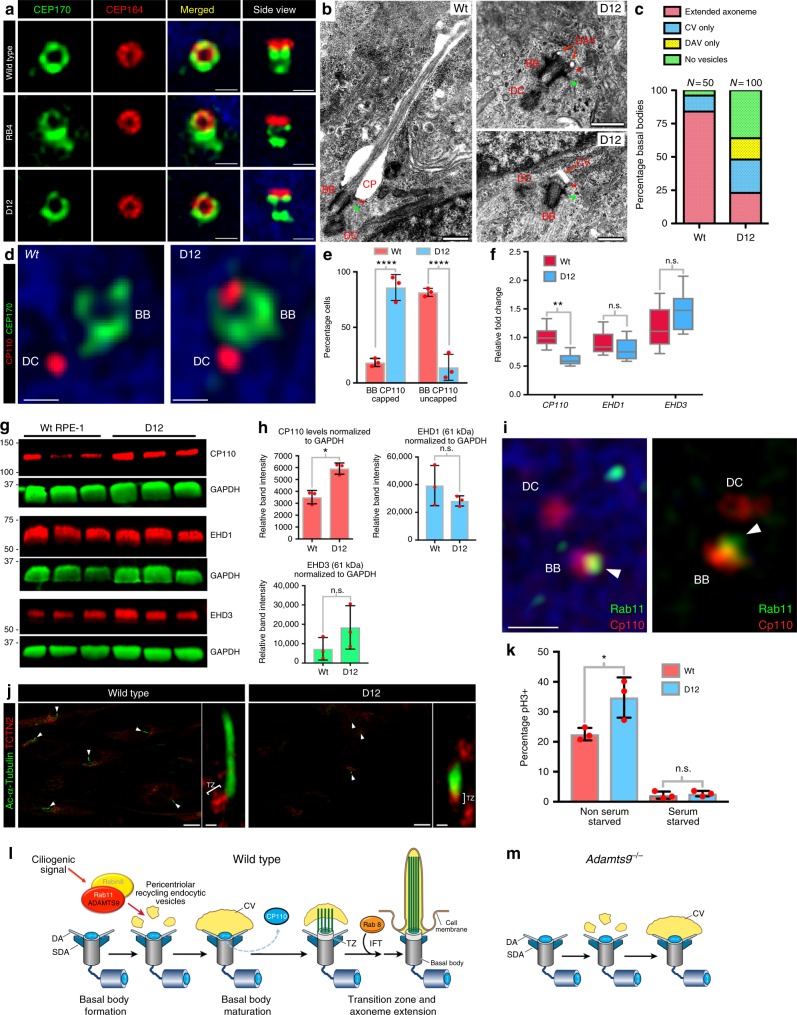
Basal body maturation is impaired in *ADAMTS9-*null cells. **a** Super-resolution imaging of distal (CEP164, red) and sub-distal appendages (CEP170, green) shows normal basal body formation in RB4 and D12 cells. **b** TEM of serum-starved wild-type and D12 cells shows D12 basal bodies (BB) with docked distal appendage vesicles (DAV) and ciliary vesicles (CV) (red arrows) but no axoneme. CP, ciliary pocket; DC, daughter centriole. **c** Quantification of basal body-associated vesicles at different stages of formation and growth shows that the majority of the mutant basal bodies do not progress beyond ciliary vesicle docking. *N* = 50 BB (WT), *N* = 100 BB (D12). **d** DSCM imaging of 24 h serum-starved wild-type and D12 cells stained for CP110 (red) and CEP170 (green) shows that the basal body of D12 cells retains the distal CP110 cap. **e** Quantification of basal bodies with and without CP110 caps (*N* = 3 independent experiments, ****p < 0.00001, Student’s *t* test). **f** Quantitative RT-PCR of *CP110, EHD1* and *EHD3* mRNAs in wild-type (Wt) and D12 RPE1 cells. *N* = 3 independent experiments; n.s., not significant; **p < 0.001, Student’s *t* test. **g**, **h** Western blot (**g**) and quantification (**h**) of CP110, EHD1 and EHD3 (red) normalized to GAPDH (green) shows higher CP110 in 24 h serum-starved *ADAMTS9* mutant D12 cells but no change in EHD1/ EHD3 levels (*N* = 3 independent experiments, *p < 0.05, Student’s *t* test). **i** DSCM imaging of 6 h serum-starved wild-type RPE-1 cells co-stained with CP110 (red) and Rab11 (green) shows Rab11 + vesicles near CP110 cap of the basal bodies (arrowheads). **j** Staining for the transition zone (TZ) marker TCTN2 shows a TZ in wild type cells and the few mutant (D12) cells that produce ciliary axonemes (arrowheads). **k** Quantification of phospho-histone H3 positive cells show increased cell proliferation in non-serum-starved mutant cells and minimal proliferation upon serum starvation, similar to wild-type cells (*N* = 3 experiments, *p < 0.05, Student’s *t* test). **l**, **m** Schematic showing the ciliogenesis sequence^33^ and arrest at the ciliary vesicle docking stage in the absence of ADAMTS9 (right). Scale bars = 500 nm in **a**–**d**, **i**. Bar charts and dotplots show mean and SD (error bars). In box and whisker plots, whiskers indicate the range and boxes mark upper and lower quartiles. The centerline indicates the mean

Immunostaining for the centrosome components CEP170 and CEP164 revealed that formation of the centrosome and its distal and SDAs was unaffected in RB4 and D12 cells (Fig. 4a). TEM of serum-starved wild-type and D12 cells showed that the majority of the mutant basal bodies lacked prominent docked ciliary vesicles (CV) whereas a significant proportion had distal appendage bound vesicles (DAV) or small CVs unfused to the cell membrane (Fig. 4b, c). Axoneme elongation requires basal body maturation, which involves removal of the centrosome distal cap protein CP110^30^ from the basal body, but not the daughter centrosome, essentially halving CP110 protein levels upon serum starvation^31^. DSCM of *ADAMTS9*-null D12 cells showed they retained CP110-staining of both centrosomes, contrasting with wild-type cells, which had a single CP110-marked centrosome (Fig. 4d, e). Quantitative RT-PCR analysis shows significantly lower levels of CP110 mRNA but western blotting showed significantly higher CP110 levels upon serum starvation in D12 cells compared to wild-type cells (Fig. 4f–h). In contrast, the endosomal membrane trafficking molecules EHD1 and EHD3, which play crucial roles in the early steps of ciliogenesis and CP110 cap removal^32^ were unaffected by the loss of *ADAMTS9* (Fig. 4f–h). Super-resolution imaging of wild-type RPE-1 cells serum-starved for 6 h to visualize early steps in ciliogenesis demonstrated Rab11+ vesicles near CP110 capped basal bodies (Fig. 4i) in agreement with previous reports. Since CP110 and the contents of Rab11+ vesicles are in distinct cellular compartments, increased CP110 observed in the mutant cells is unlikely due to direct proteolytic activity of ADAMTS9, which is intra-vesicular. The few short cilia found in D12 cells formed a transition zone marked by tectonin-2 immunostaining (Fig.  4j). Although D12 cells had a higher percentage of phospho-histone H3 positive cells in serum-replete cultures, their proliferation was comparable to wild-type cells after starvation (Fig. 4k). Our findings thus define the point of ADAMTS9 impact as occurring during ciliogenesis and directly affecting basal body maturation and ciliary vesicle growth stages (Fig. 4l, m)^33^.

### *Adamts9* and *Adamts20* combined mutants resemble ciliopathies

**Fig. 5:**
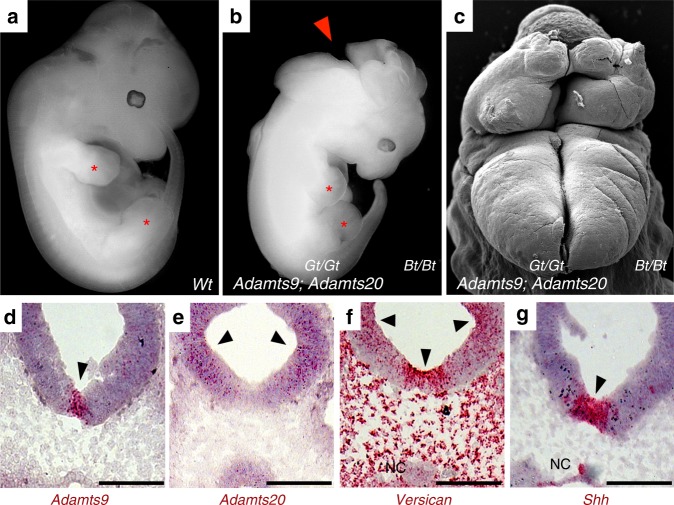
Severe developmental defects resembling ciliopathies in *Adamts9*^*Gt/Gt*^*; Adamts20*^*Bt/Bt*^ mouse embryos. **a** E12.5 wild-type and *Adamts9*^*Gt/Gt*^*;Adamts20*^*Bt/Bt*^ embryos (**b**) showing occipital exencephaly (red arrowhead) and a malrotated body axis in the latter. Red asterisks indicate right-side limbs, which are abnormally close in the mutant (**b**). **c** Scanning electron micrograph of the exencephalic hindbrain of an E12.5 *Adamts9*^*Gt/Gt*^*;Adamts20*^*Bt/Bt*^ embryo. **d**–**g** RNAscope^®^ in situ hybridization shows expression domains of *Adamts9* (**d**), *Adamts20* (**e**), their cognate ECM substrate *Vcan* (**f**) and *Shh* (**g**) in the rostral neural epithelium of E9.5 embryos. Robust *Adamts9* expression is observed in the rostral floor plate (arrowhead in **d**) within the *Shh* expression domain (arrowhead in **g**). *Adamts20* is predominantly expressed in the lateral neural epithelium (arrowheads in **e**). *Vcan* is strongly expressed in the floor plate and the lateral neural epithelium (lateral arrowheads in **f**), with strong expression in surrounding craniofacial mesenchyme. NC, notochord. Scale bars = 100 μm in **d**–**g**

To determine the role of ADAMTS9 and ADAMTS20 on ciliogenesis and the resulting functional impact on morphogenesis, we analyzed mouse mutants of these proteases. Because of early lethality of *Adamts9*-null mutants, we utilized a hypomorphic allele, *Adamts9*^Gt^, which survives to 15 days of gestation (E15)^17^. In combination with the *Adamts20*^*Bt*^ allele we found a severe morphogenetic defect suggesting defective ciliogenesis. Specifically, *Adamts9*^Gt/Gt^*; Adamts20*^Bt/Bt^ embryos, which also survived until 15 days of gestation, had severe developmental anomalies resembling ciliopathy mutations in mice (Supplementary Movies 1–4), including occipital exencephaly, indicative of their cooperative action in neural tube closure (Fig. 5a–c, Supplementary Fig. 4a–d). Other major developmental anomalies in these mutants included mandibular hypoplasia, eye defects, abnormal trachea-esophageal septation, increased facial width, cleft palate, cleft lip, and lateral asymmetry with the right-sided limbs abnormally close and the left-side limbs much further apart (Supplementary Fig. 4e–h, Supplementary Movies 1–4). Body axis distortion led to visceral heterotaxy in mutant embryos (Supplementary Movies 3,4). *Adamts9*^Gt/Gt^ embryos also had cleft palate (Supplementary Fig. 4i–l), and further *Adamts9* dosage reduction via combination of *Adamts9*^Gt^ with the inactivating *Adamts9*^Del^ allele (*Adamts9*^Gt/Del^) resulted in failure to undergo rotation, an open neural tube and death by E10.5 (Supplementary Fig. 4m–p).

Intriguingly*, Adamts9* was strongly expressed in the midline neural floor plate from the onset of neural tube formation at E8.5 days until E11.5 but at lower levels in the lateral neural epithelium (Fig. 5d, Supplementary Fig. 5a). *Adamts20* is expressed more laterally than *Adamts9* in the neural epithelium and at much lower levels in the ventral midline (Fig. 5e, Supplementary Fig. 5a). *Adamts9* and *Adamts20* mRNA overlapped with mRNA encoding their cognate substrate versican^12^, which was expressed strongly in the ventral midline embracing the *Adamts9* domain, and in lateral neural epithelium (Fig. 5f) coinciding with *Adamts20*. The *Adamts9* expression domain tightly coincided with the ventral floor plate *Shh* expression domain (Fig. 5g), but did not overlap with notochord *Shh* expression (Supplementary Fig. 5a). Along the length of the neural tube, *Adamts9* expression was limited to the floor plate in the hindbrain region and was absent further rostrally and caudally (Supplementary Fig. 5b), whereas *Adamts20* showed extensive rostral−caudal expression^14^ and was expressed in the midline floor plate caudally (Supplementary Fig. 5a). The exclusive hindbrain exencephaly of *Adamts9*^*Gt/Gt*^*; Adamts20*^*Bt/Bt*^ mutants and *Adamts9*^*Gt/Del*^ mutants further suggested a local role for ADAMTS9 alone as well as in partnership with ADAMTS20. Intriguingly, expression of *Shh* and *Vcan* were interdependent, i.e., *Vcan*^hdf/hdf^ embryos (a null mutant^34^) had reduced *Shh* expression and *Shh-*null mice lacked *Vcan* mRNA in the neural tube epithelium (Supplementary Fig. 5c,d).

### *Adamts9*^*Gt/Gt*^*; Adamts20*^*Bt/Bt*^ embryos have ciliary defects

**Fig. 6:**
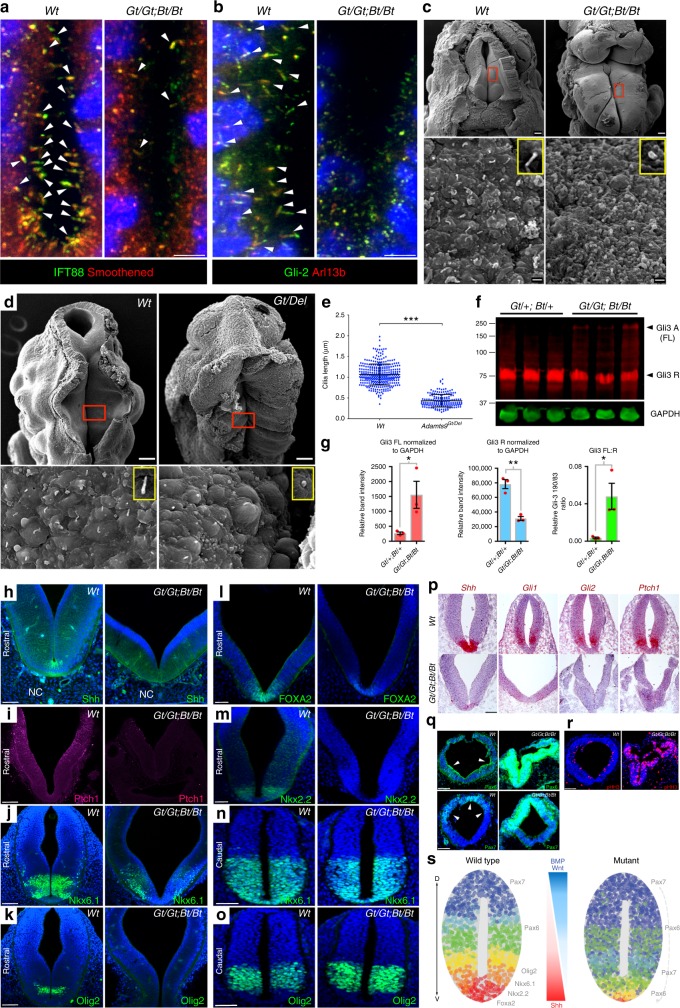
*Adamts9*^*Gt/Gt*^*; Adamts20*^*Bt/Bt*^ embryos have impaired ciliogenesis and Shh signaling. **a**, **b** Coimmunostaining for IFT88 (green) and Arl13b (red) with smoothened (red), and Gli-2 (green) respectively, reveals fewer cilia and reduced Hh signaling in E9.5 *Adamts9*^*Gt/Gt*^*; Adamts20*^*Bt/Bt*^ neural tubes. **c** Scanning electron microscopy (SEM) of E12.5 wild type and *Adamts9*^*Gt/Gt*^*; Adamts20*^*Bt/Bt*^ neural tubes shows short cilia in mutants. Boxed areas are shown at higher magnification in lower panels. Inserts (yellow) in the lower panels show single cilia. **d** SEM of E9.5 *Adamts9*^*Gt/Del*^ embryos shows an open neural tube and short cilia. Red boxes are shown at higher magnification in the lower panels. Inserts in the lower panels (yellow box) show single cilia. **e** Quantification of primary cilium length in wild-type and *Adamts9*^*Gt/Del*^ embryos shows short cilia in the mutants (*N* = 3 embryos, ***p < 0.001, Student’s *t* test). **f** Gli3 western blot of E12.5 *Adamts9*^*Gt/+*^*; Adamts20*^*Bt/+*^ and *Adamts9*^*Gt/Gt*^*; Adamts20*^*Bt/Bt*^ heads shows reduced processed Gli3 (83 kDa) in mutants. **g** Quantification of relative band intensities for full-length Gli3 (190 kDa) and processed Gli3 (83 kDa) form and the relative ratios (*N* = 3 embryos, *p < 0.05, **p < 0.001, Student’s *t* test). **h**–**m** Rostral neural tube sections of E9.5 wild-type and *Adamts9*^*Gt/Gt*^*; Adamts20*^*Bt/Bt*^ embryos immunostained for Shh (**h**), Ptch1 (**i**), Nkx6.1 (**j**), and Olig-2 (**k**), FOXA2 (**l**), Nkx2.2 (**m**) shows reduced Shh signaling and aberrant ventralization in mutants (*N* = 3 embryos each). NC, notochord. **n**, **o** Nkx6.1 (**n**) and Olig-2 (**o**) staining (green) of caudal neural tube sections show normal Shh signaling caudally in mutants (N  = 3 embryos each). **p** ISH for *Shh, Gli1, Gli2* and *Ptch1* mRNA shows reduced Shh-responsive mRNAs in *Adamts9*^*Gt/Gt*^*; Adamts20*^*Bt/Bt*^ rostral neural tube (*N* = 3 embryos each for each probe). **q** BMP-activated dorsal neuronal markers Pax6 and Pax7 (green) show stronger signal and ventral expansion in *Adamts9*^*Gt/Gt*^*; Adamts20*^*Bt/Bt*^ embryos. **r** Phospho-histone H3 (pHH3, red) staining shows more proliferating cells in the *Adamts9*^*Gt/Gt*^*; Adamts20*^*Bt/Bt*^ rostral neural tube. **s** Cartoon depicting neuronal domains in wild-type and mutant neural tubes. Scale bars = 50 μm in **h**–**r**, 5 μm in **a**, **b** and 100 μm and 1 μm in **c**, **d**. Bar charts and dotplots show mean (centerline) and SD (error bars) except **g** (S.E.M.)

Confocal microscopy Z-stacks of thick vibratome sections coimmunostained for Smo and the ciliary axoneme marker IFT88 showed few primary cilia in *Adamts9*^*Gt/Gt*^*; Adamts20*^*Bt/Bt*^ neural tubes, whereas Smo localization to well-formed cilia was seen in the wild-type neural tube (Fig. 6a, Supplementary Fig. 6a,b). In wild type but not *Adamts9*^*Gt/Gt*^*; Adamts20*^*Bt/Bt*^ neural tubes Gli2-stained cilia were also marked by Arl13b (Fig. 6b, Supplementary Fig. 6c,d). Scanning electron microscopy (SEM) demonstrated rudimentary primary cilia in *Adamts9*^*Gt/Gt*^*; Adamts9*^*Bt/Bt*^ and *Adamts9*^Gt/Del^ neural tubes (Fig. 6c–e). A hallmark of mouse cilium mutants is impairment of Gli3 processing, which occurs in primary cilia^35–38^. Western blots showed more unprocessed Gli3 (Gli3-190), and less processed Gli3 repressor (Gli3-83) in *Adamts9*^Gt/Gt^*; Adamts20*^Bt/Bt^ heads (Fig. 6f, g). Signaling by all three Hh ligands, *Shh*, *Ihh* and *Dhh* relies on the primary cilium^3^. Upon Hh binding to Ptch1, repression of Smoothened (Smo) is relieved and it relocates to the primary cilium to activate Gli transcription factors^39^. Reduced staining of Shh and the Shh receptor Patched-1, itself a Shh target, was seen in the *Adamts9*^Gt/Gt^*;Adamts20*^Bt/Bt^ rostral neural epithelium (Fig. 6h, i). The ventral neuronal markers activated by Shh signaling, Nkx6.1, Olig2, FOXA2 and Nkx2.2 were severely downregulated or aberrantly distributed in the mutant rostral neural tubes (Fig. 6j–m). Nkx6.1 and Olig2 staining of caudal neural tube sections, however, showed comparable staining in wild-type and mutant embryos indicating that impairment of Shh signaling was spatially limited to the rostral neural tube (Fig. 6n, o). RNA ISH analysis of *Shh*, *Gli1*, *Gli2* and *Ptch1* confirmed the downregulation of Shh responsive gene transcription in E9.5 mutant rostral neural tubes (Fig. 6p). Positional identity of neural epithelial cells is determined by counter-gradients of Shh (from the floor plate) and BMP/Wnt signaling (from the roof plate)^40,41^. Exencephaly with reduced ventralization and ventral encroachment of dorsal neuronal markers is a characteristic finding in mouse cilium defects arising from mutated intraflagellar transport (IFT) proteins^37^, centrosome proteins^42^ and transition zone proteins^43^. Indeed, the dorsal neuronal markers Pax6 and Pax7 showed increased intensity and ventral expansion in the mutant neural tubes (Fig. 6q), accompanied by increased, ventrally expanded *Bmp4* expression (Supplementary Fig. 6k,l), with normal expression of the BMP inhibitor *Noggin* (Supplementary Fig. 6m,n). As in other exencephalic mutants *Adamts9*^Gt/Gt^*; Adamts20*^Bt/Bt^ neural tubes also showed increased cell proliferation (Fig. 6r). Since the observed neural epithelial cells of the mutants indeed do possess cilia, albeit short and bulbous, the observed ciliated cells are nonproliferating and thus excess proliferation is likely not causative in shortening of the cilium in the mutants. We therefore concluded that dysregulated Shh and BMP signaling as a result of reduction of primary cilia in the hindbrain was the likely mechanism of *Adamts9*^Gt/Gt^*; Adamts20*^Bt/Bt^ neural tube defects (Fig. 6s). Genetic interaction of *Adamts9* with *Ptch1* was tested and showed that *Adamts9*^Gt/Gt^; *Ptch1*^*l*acZ/+^ embryos consistently (3/3) developed occipital exencephaly, lateral asymmetry and abnormally positioned limbs similar to *Adamts9*^Gt/Gt^*; Adamts20*^Bt/Bt^ mutants (Supplementary Fig. 6e–g). *Adamts20*^Bt/Bt^; *Ptch1*^*lacZ*/+^ newborns (11/11) had fully penetrant cleft palate (Supplementary Fig. 6h,i), a phenotype previously observed in 100% of *Adamts20*^Bt/Bt^; *Adamts9*^Gt/+^ mutants and 3% of *Adamts20*^Bt/Bt^ mice^16,17^. Furthermore, *Adamts9*^Gt/+^; *Ptch1*^*l*acZ/+^ mice had highly penetrant hind limb preaxial polydactyly (72%, 8/11) (Supplementary Fig. 6j), a phenotype observed in only 2% of *Ptch1*^*l*acZ/+^ mice^44^. Both *Adamts9* and *Adamts20* are expressed in the developing limb buds and are crucial for web regression^18,45,46^ but polydactyly was seen neither after conditional deletion of *Adamts9*^45^, nor in *Adamts9*^Gt/Gt^*; Adamts20*^Bt/Bt^ embryos.

### ECM alterations in *Adamts9*^Gt/Gt^*; Adamts20*^Bt/Bt^ neural tubes

**Fig. 7:**
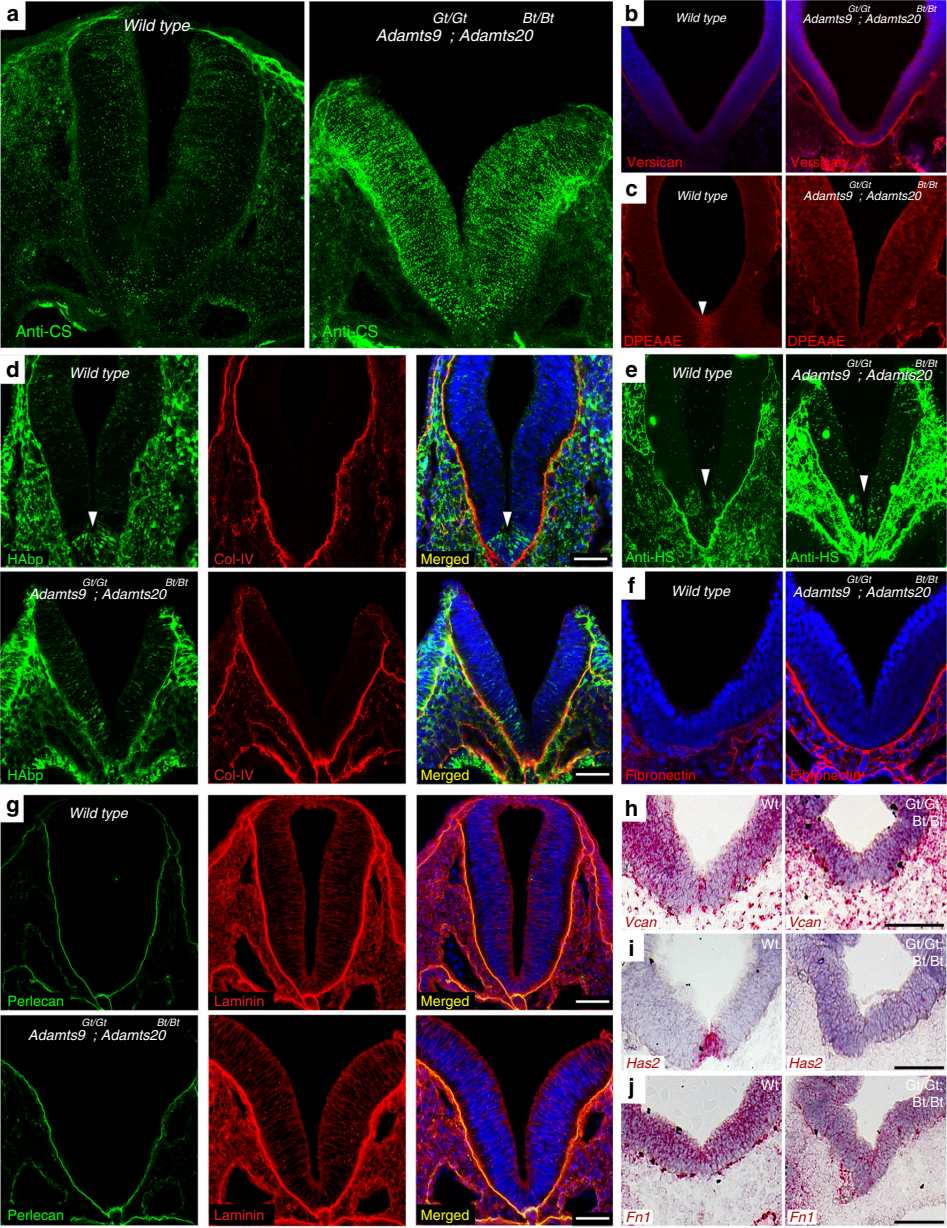
Abnormal ECM dynamics in *Adamts9*^*Gt/Gt*^*; Adamts20*^*Bt/Bt*^ neural epithelium. **a** 3D-projection of 30 μm-thick vibratome sections stained for chondroitin sulfate (CS) in E9.5 wild-type and *Adamts9*^*Gt/Gt*^*; Adamts20*^*Bt/Bt*^ embryos shows intense CS staining in interstitial ECM of mutant neural epithelium. **b** Immunostaining for versican (GAG β) shows stronger staining throughout mutant neural epithelium. **c** The versican-neo epitope antibody anti-DPEAAE shows strong staining in the midline of the wild-type neural tube floor plate (arrowhead) but not in the mutant. **d** Coimmunostaining with hyaluronan-binding protein (HAbp) (green) and anti-collagen IV (red) shows loss of hyaluronan in the mutant floor plate. Collagen IV in the basement membrane of the neural epithelium shows no change in the mutant. **e** Anti-heparan sulfate (anti-HS) shows intense staining in the mutant craniofacial mesenchyme around the neural epithelium and to a lesser degree in the neural tube (arrowhead). **f** Fibronectin is present in wild-type neural tube basement membrane and surrounding mesenchyme where it stains strongly in the mutant. **g** Perlecan (green) and laminin (red) show comparable staining of wild-type and mutant basement membranes. Laminin is also present in ECM of the craniofacial mesenchyme. **h**–**j** RNA in situ hybridization shows loss of *Has2* (**i**) in the mutant neural tube whereas *Vcan* (**h**) and *Fn1* (**j**) expression are unaffected. Scale bars = 50 μm in (**d**–**g**), 100 μm in (**h**–**j**)

Since the cognate function of ADAMTS9 and ADAMTS20 is ECM remodeling, we examined if loss of ADAMTS9 and ADAMTS20 affected ECM dynamics of the mouse neural epithelium, in addition to their role in ciliogenesis. Versican, an ADAMTS9 and ADAMTS20 substrate, is a large secreted chondroitin sulfate (CS) proteoglycan^47,48^. Intense CS staining and increased versican staining were seen in the *Adamts9*^Gt/Gt^*; Adamts20*^Bt/Bt^ neural tube at E9.5 (Fig. 7a, b, Supplemental movie 5,6) and versican proteolysis revealed by the neo-epitope antibody anti-DPEAAE was reduced in the floor plate^49^ (Fig. 7c). Versican binds to the glycosaminoglycan hyaluronan (HA)^50^ and *Has2*, the major HA synthase, is a direct target of the Shh signal transducers Gli1 and Gli2^51^. In wild-type neural tubes HA was concentrated in the floor plate, a distribution perturbed in *Adamts9*^Gt/Gt^*; Adamts20*^Bt/Bt^ neural tubes (Fig. 7d). Increased HS-staining was seen in the mutant neural epithelium and surrounding mesenchyme (Fig. 7e). Consistent with a potential role in HS-proteoglycan turnover suggested by this finding, we observed direct ADAMTS9 binding to heparin and embryo-derived heparan sulfate (Supplementary Fig. 6o,p). Fibronectin, a recently identified ADAMTS9 substrate (Wang et al., submitted) also stained more strongly in the mutant neural tube basement membrane and mesenchyme (Fig. 7f), whereas basement membrane molecules collagen IV, perlecan, and laminin were unaffected (Fig. 7d, g). Thus, proteoglycan−hyaluronan distribution in the neural tube was selectively affected by loss of *Adamts9* and *Adamts20*. *Has2* mRNA expression was lost in the mutant neural tube floor plate as expected of an Shh target^51^, whereas *Vcan* and *Fn1* mRNA were unaffected (Fig. 7h–j).

### Abnormal ciliary signaling and ECM in *Adamts9*^Gt/Gt^ yolk sac

**Fig. 8:**
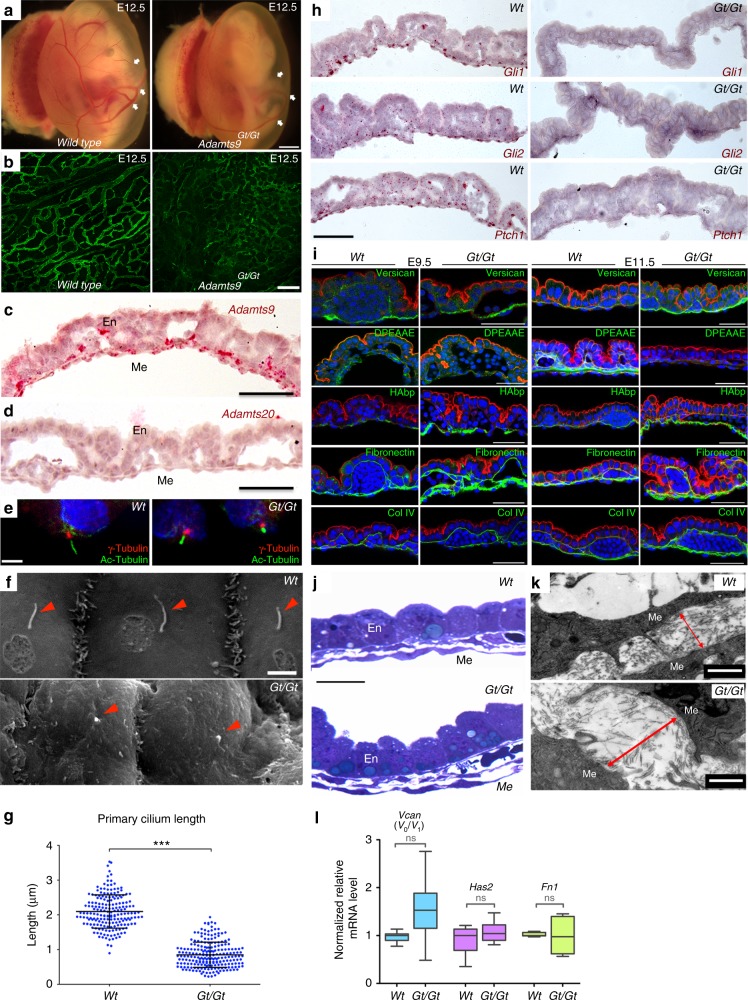
Defective angiogenesis, cilia, Hh signaling and ECM in *Adamts9*^*Gt/Gt*^ yolk sac. **a** E12.5 *Adamts9*^*Gt/Gt*^ embryos have poorly formed yolk sac vasculature. White arrows point to vitelline vessel branch points. **b** En-face images of isolectin-B4-stained (green) whole-mount yolk sacs show abnormal vasculature in *Adamts9*^*Gt/Gt*^ yolk sacs. **c**, **d** RNA in-situ hybridization shows robust *Adamts9* expression (red) in E11.5 mesoderm (ME) but not yolk sac endoderm (En). *Adamts20* (**d**) is not expressed in the yolk sac. **e** Primary cilia and centrosomes visualized by acetylated α-tubulin (green) and γ-tubulin (red) respectively, or SEM (**f**, arrowheads) show short bulbous-tipped primary cilia in *Adamts9*^*Gt/Gt*^ yolk sac mesothelium. **g** Quantitation of primary cilium length by SEM in wild-type and *Adamts9*^*Gt/Gt*^ yolk sac mesothelium (*N* = 3 yolk sacs from each genotype, ***p < 0.001, Student’s *t* test). **h** In-situ hybridization for *Gli1*, *Gli2*, and *Ptch1* mRNA in E11.5 yolk sacs (red) shows absent hedgehog signaling in *Adamts9*^*Gt/Gt*^ yolk sacs (*N* = 3 yolk sacs from each genotype). **i** E9.5 and E11.5 *Adamts9*^*Gt/Gt*^ yolk sacs were stained for versican, cleaved versican (DPEAAE), hyaluronan (HAbp), fibronectin, and collagen-IV, and counterstained with Alexa-568 phalloidin (red) to outline cell layers. Versican, hyaluronan and fibronectin accumulated in yolk sac mesoderm whereas staining for versican neo-epitope DPEAAE was lost in the *Adamts9*^*Gt/Gt*^ yolk sac. Collagen IV staining was unaffected (*N* = 3 yolk sacs from each genotype). **j** Toluidine blue staining of 1 μm plastic-embedded sections of E11.5 yolk sacs show a thicker mesoderm in the mutant resulting from expanded ECM. **k** TEM images of the yolk sac show increased ECM between mesoderm cells (red arrows) in the *Adamts9*^*Gt/Gt*^ yolk sac, primarily noncollagenous in appearance. **l** qRT-PCR analysis for *Vcan, Has2* and *Fn1* shows comparable levels in mutant and wild-type yolk sac (*N* = 3 yolk sacs from each genotype, Student’s *t* test). Scale bars = 2 mm in **a**, 100 μm in **b**, 50 μm in **c**, **d** and **h**, **i**, 2 μm in **e**, **f**, 10 μm in **j**, **k**, and 1 μm in the lower panels of **k**. Dotplot shows mean and SD. In box and whisker plot, whiskers indicate the range and boxes mark upper and lower quartiles. The centerline indicates the mean

The primary cilium mediates Ihh signaling during yolk sac angiogenesis^52^. Like *Ihh*^−/−^ embryos, *Adamts9*^*Gt/Gt*^ embryos had poorly developed yolk sac microvasculature (Fig. 8a, b). *Adamts9* was specifically expressed in yolk sac mesoderm, including the mesothelium (Fig. 8c), whereas *Adamts20* mRNA was absent in the yolk sac (Fig. 8d). Confocal and SEM imaging showed shorter primary cilia in *Adamts9*^*Gt/Gt*^ yolk sac mesothelium (Fig. 8e–g). In-situ hybridization showed that Hh responsive mRNAs *Gli1*, *Gli2*, and *Ptch1*, expressed by mesoderm cells in wild-type yolk sac, were absent in *Adamts9*^*Gt/Gt*^ yolk sacs (Fig. 8h). *Adamts9*^*Gt/Gt*^ yolk sacs had more intense versican, fibronectin and hyaluronan staining and reduced versican cleavage, whereas collagen IV staining was unaltered (Fig. 8i). Consistent with ECM accumulation in the mutants, yolk sac mesoderm was thicker owing to an excess of amorphous, i.e., nonfibrillar ECM (Fig. 8j, k). RT-qPCR analysis of *Vcan, Has2* and *Fn1* was comparable, suggesting that versican and fibronectin accumulation ensued from reduced proteolytic turnover (Fig. 8l). Together, the findings from *Adamts9*^Gt/Gt^; *Adamts20*^Bt/Bt^ and *Adamts9*^Gt/Del^ neural tube and *Adamts9*^Gt/Gt^ yolk sac indicate a dual role for ADAMTS9 and ADAMTS20, i.e., in ciliogenesis as well as ECM turnover.

### Cilium defects are independent of versican proteolysis

**Fig. 9:**
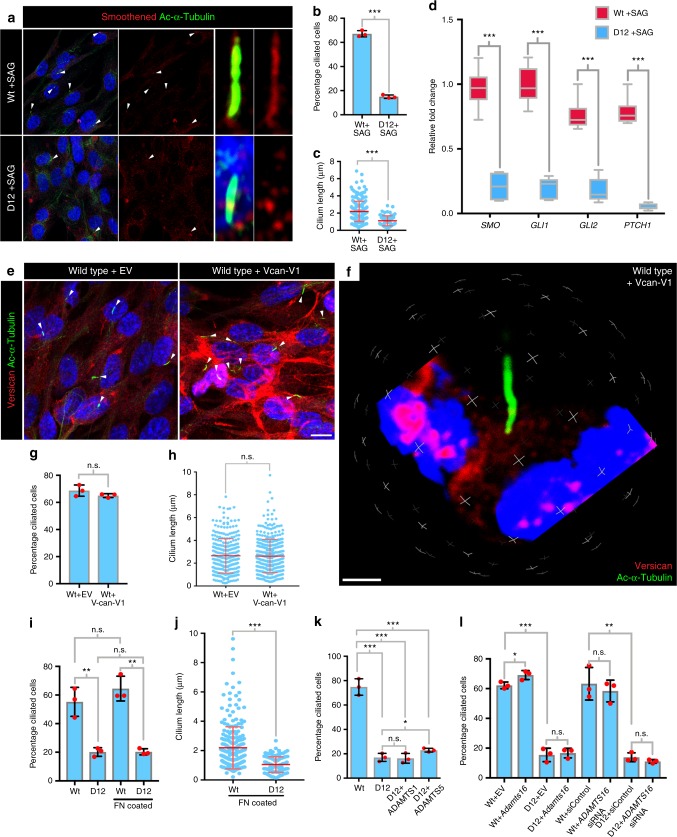
Cilium defects in *ADAMTS9*-null cells are independent of Shh signaling and versican cleavage. **a** Smoothened (red) and acetylated α-tubulin (green) coimmunostaining of serum-starved wild-type RPE-1 and D12 cells treated with 500 nm SAG shows cilium-localized SMO in wild-type but not mutant cells. Arrowheads indicate primary cilia. **b** Percentage of ciliated cells in wild-type RPE-1 and D12 cells treated with 500 nm SAG (*N* = 3 independent experiments, ***p < 0.0001, Student’s *t* test). **c** Quantification of cilium length in wild-type and D12 cells after treatment with 500 nm SAG shows no rescue of length (*N* = 3 independent experiments, ***p < 0.0001, Student’s *t* test). **d** qRT-PCR of *SMO, GLI1, GLI2* and *PTCH1* after 500 nm SAG treatment of wild-type and D12 cells shows attenuated response in mutant cells (*N* = 3 independent experiments, ***p < 0.0001, Student’s *t* test). **e**, **f** Coimmunostaining of versican (red) and acetylated α-tubulin (green) in wild-type cells transfected with empty vector or *VCAN*-V1 shows increased versican around wild-type cells without an effect on ciliation (arrowheads). **g** Percentage of cells with primary cilia in wild-type cells transfected with empty vector or *VCAN*-V1 (*N* = 3 independent experiments, Student’s *t* test). **h** Quantification of primary cilium length in wild-type cells transfected with empty vector or *VCAN*-V1 (*N* = 3 independent experiments, Student’s *t* test). **i** Quantification of percentage of ciliated cells in wild-type and D12 cultures on 20 μg/mL fibronectin-coated or noncoated culture slides has no effect on wild-type or D12 cells (*N* = 3 independent experiments, **p < 0.01, Student’s *t* test). **j** Quantification of primary cilium length in wild-type and D12 cells cultured on 20 μg/mL fibronectin-coated slides did not restore D12 cilium length (*N* = 3 independent experiments, ***p < 0.0001, Student’s *t* test). **k** Transfection of ADAMTS1 or ADAMTS5 failed to rescue ciliogenesis in D12 cells (*N* = 3 experiments, ***p < 0.0001; *p < 0.05, Student’s *t* test). **l** Overexpression of *Adamts16* or *ADAMTS16* depletion failed to rescue ciliogenesis in D12 cells (*N* = 3 independent experiments, ***p < 0.00001; **p < 0.001; *p < 0.05, Student’s *t* test). Dotplot shows mean and SD. In box and whisker plot, whiskers indicate the range and boxes mark upper and lower quartiles. The centerline indicates the mean

We tested if the ADAMTS9-null RPE-1 cells had similar impairment of Shh responsiveness and versican remodeling and if the latter might have a role in perturbing ciliogenesis. Serum-starved wild-type and D12 cells were treated with the 500 nm smoothened agonist (SAG) for 12 h to induce Shh signaling. Staining with an SMO antibody and acetylated α-tubulin showed ciliary axoneme localized SMO staining in wild-type cells and a lack of axoneme staining in D12 cells (Fig. 9a). Treatment with SAG neither rescued the percentage of ciliated cells nor their length in D12 cells (Fig. 9b, c). Quantitative RT-PCR revealed significantly low levels of Shh responsive (*Ptch1, Gli1*) gene expression in D12 cells in response to SAG treatment, although *Smo*, which is not a known Shh target, was also unexpectedly downregulated (Fig. 9d).

RPE-1 cells had low levels of *Vcan* mRNA expression and correspondingly, versican immunostaining, even in *ADAMTS9* knockout D12 cells, during the 2-day culture period utilized for ciliogenesis assays (Supplementary Fig. 7a,b). However, upon culturing wild-type and D12 cells for 5 days we did observe versican accumulation in *D12* cells and greater versican cleavage in wild-type cells (Supplementary Fig. 7c). Therefore, to test if increased pericellular versican could affect ciliogenesis, we overexpressed versican in RPE-1 cells (Fig. 9e, f). Despite the high level of versican resulting in their pericellular matrix, neither the percentage of ciliated cells nor cilium length were affected (Fig. 9g, h). Similarly, culturing either wild-type or D12 cells upon 20 μg/mL fibronectin-coated wells had no impact on ciliogenesis of wild-type cells nor did it rescue ciliogenesis in the D12 cells (Fig. 9i, j) (Supplementary Fig. 7d). Transfection of D12 cells with ADAMTS1 and ADAMTS5, which also cleave versican, did not restore ciliogenesis to wild-type levels, further supporting the conclusion that impaired processing of versican was not causally relevant to reduced ciliogenesis in the absence of ADAMTS9 (Fig. 9k). Previously genetic inactivation of ADAMTS16, a phylogenetically distantly related protease in rats, was associated with increased cilium length in renal tubular epithelium^53^. However, overexpression of *Adamts16* in D12 cells did not rescue the ciliogenesis defect, although it did increase cilium length in wild-type cells. Conversely, knockdown of *ADAMTS16* had no impact on ciliogenesis in wild-type or D12 cells (Fig. 9l).

## Discussion

**Fig. 10:**
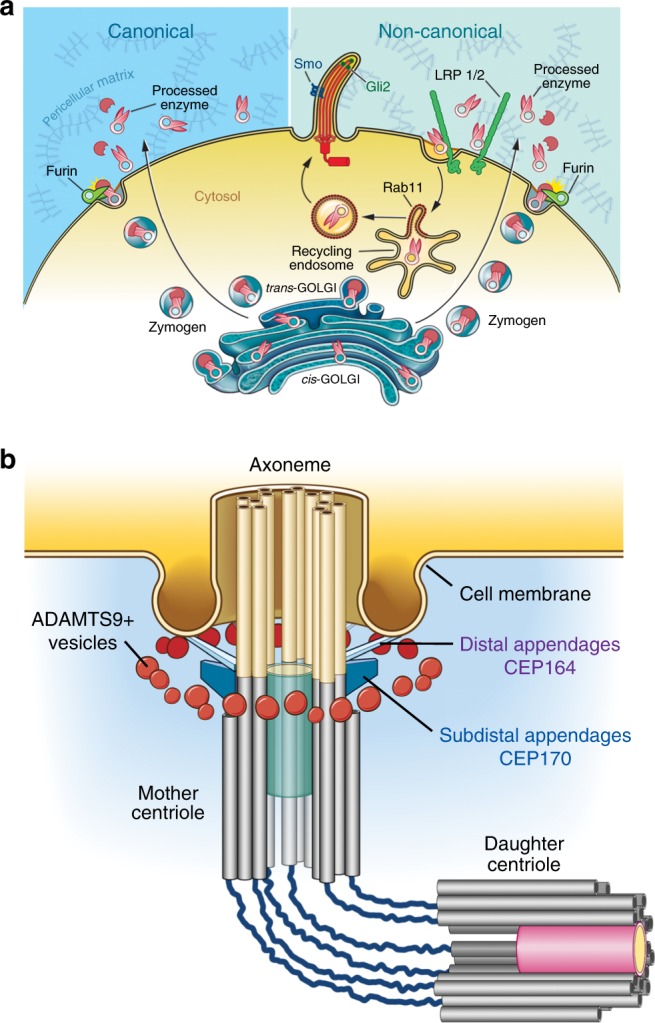
The voyage of ADAMTS9 to the base of the primary cilium. **a** Schematic summarizing the life cycle and dual role of ADAMTS9 in ECM remodeling (left-hand side) and ciliogenesis (right-hand side). **b** Schematic illustrating the geographical distribution of ADAMTS9 vesicles at the ciliary base

Here, we show that ADAMTS9 working alone, or in partnership with ADAMTS20 where they are coexpressed is essential for ECM turnover and ciliogenesis during mammalian morphogenesis (Fig. 10a). The severe impact of their deficiency on cilium biogenesis provides an obvious mechanism for the observed impairment of Shh and Ihh signaling in the neural tube and yolk sac respectively. The findings also clearly illustrate a tissue-specific threshold for ADAMTS9 in ciliogenesis, i.e., in the yolk sac, where *Adamts20* is not expressed, deficiency of *Adamts9* alone impairs ciliogenesis and when *Adamts9* dosage was substantially reduced below that of *Adamts9*^Gt/Gt^ embryos in *Adamts9*^Gt/Del^ embryos, open neural tube and defective cilia also resulted. We conclude that many cells may be over-engineered to ensure availability of ADAMTS9 and/or ADAMTS20 above a required threshold, and that both proteases may be broadly relevant to ciliogenesis. Indeed, *ADAMTS9* was previously identified as a positive regulator of ciliogenesis in an siRNA screen for putative ciliary length regulators^7^, but not investigated further and has been recently implicated as the mutant gene in human ciliopathies affecting the kidney and brain^54^.

Incorporating prior work on ADAMTS9 biosynthesis and trafficking^12,17,20,55–58^ as well as new data on heparin binding and receptor-mediated endocytosis, the present work tracks the voyage of ADAMTS9 from the secretory pathway to cilium (Fig. 10a). ADAMTS9, having undergone obligate, regulatory glycosylation in the secretory pathway^56,58^, binds to the cell surface, which may be via HSPG, LRP1/2 and furin interaction^12,20^ and is activated by furin^20^. It is then recycled to the ciliary vesicles via LRP1/2 and clathrin-mediated endocytosis to regulate ciliogenesis via accumulation around the basal body (Fig. 10b). ADAMTS20 has not been as extensively investigated, but the limited data clearly suggest that it too is internalized to participate in ciliogenesis.

Considerable evidence argues against unconventional ADAMTS9 transfer into the cytosol. ADAMTS9 has a bona fide signal peptide and transfected ADAMTS9 constructs are readily detected at the cell surface and in the medium^12,56,58^, bound to secretory chaperones^57^ and modified by Golgi enzymes^20,56,58^. That ADAMTS9 present at the cilium base lacks its propeptide strongly supports recycling of secreted ADAMTS9, since its propeptide is excised at the cell surface and not in the *trans*-Golgi^20^. Notably, CHC-knockdown or inhibition of clathrin-mediated endocytosis removed ADAMTS9 from the cilium base. Also arguing against an alternative mRNA spliced cytosolic ADAMTS9 proteoform, ciliogenesis was rescued in D12 cells by transfection with the canonical ADAMTS9 and ADAMTS20 cDNA. ADAMTS9^Gt^ arose from a gene trap insertion that leads to constitutive ADAMTS9 membrane anchorage with impaired furin processing^17^. Since ADAMTS9^Gt/Gt^ yolk sac mesothelium lacks cilia, we conclude that release from the cell and propeptide excision by furin are crucial for ADAMTS9 function. The rescue of ciliogenesis in ADAMTS9-deficient RPE1 cells by ADAMTS9 supplied in conditioned medium and localization of exogenous ADAMTS9 to the cilium base conclusively establishes the constitutively secreted protein as the participant in ciliogenesis.

*Adamts9* and *Adamts20* mRNA expression domains had minimal overlap in the neural tube, yet *Adamts9*^Gt/Gt^ embryos completed neural tube closure. Here, it is possible that low-level *Adamts20* expression, or secreted ADAMTS20 acting *in-trans* compensates for reduced ADAMTS9 activity. Indeed, in RPE1 cells, the present work unequivocally demonstrates that ADAMTS9 and ADAMTS20 are interchangeable, and can act in *trans*. ADAMTS9 and ADAMTS20 catalytic activity is essential for their function at the ciliary base, but whether it functions *in-cis*, i.e., via autocatalysis of each protease to a functionally optimal product essential for ciliogenesis, or by proteolysis of an intravesicular substrate to activate its role in ciliogenesis remains to be elucidated. In theory, ADAMTS9/ADAMTS20 localized to the Rab11+ vesicles, could proteolytically modify the ectodomain of a transmembrane molecule necessary for ciliogenesis.

The role of endocytic trafficking in ciliary vesicle development is poorly understood and few secreted proteins participating in ciliogenesis are known. A seminal study of ciliogenesis identified a ciliary vesicle adjacent to the nascent axoneme and determined that it was of Golgi origin^59^, yet recycling endosome markers also label peri-centrosomal vesicles^7^. Knockdown of PTPN23, a nontransmembrane tyrosine phosphatase required for endosomal cargo sorting in early endocytic compartments^60^, and ASAP1, a GTPase-activating protein required for peri-centrosomal accumulation of endocytic vesicles^61^, significantly reduced the number of ciliated RPE-1 cells. A secreted phospholipase PLA2G3, is a negative ciliogenesis regulator^7^ and PI3K-C2-α, a phosphatidylinositol 3-kinase, regulates endocytic trafficking to the cilium and ciliogenesis^62^. To our knowledge the only other secreted molecule associated with a cilium defect is laminin-511^63^. Although PTPN23, PI3K-C2-α and PLA2G3 localize in peri-centromeric endocytic vesicles^7,62^, laminin-511 is not known to do so and may act indirectly by ensuring apical-basal polarity of epithelial cells, which manifest short primary cilia in its absence^63^. ADAMTS9 is concentrated in a distinct subset of Rab11+ late-recycling endocytic vesicles surrounding the basal body. Together, with Rab8, a master regulator of ciliary protein trafficking, Rab11+ vesicles transport cargo bound for the cilium during ciliogenesis (reviewed by ref. ^64^). ADAMTS9-containing vesicles comprise a unique population lying external to previously identified concentric domains surrounding the centrosome. ADAMTS20 trafficking was not investigated in the same detail, since its ability to rescue of ciliogenesis in the D12 cells and comparable localization as ADAMTS9 to the cilium base suggests it follows a similar pathway.

ADAMTS9 and ADAMTS20 cooperate with ADAMTS5 in versican proteolysis during interdigit web regression^18^, but ADAMTS5 did not rescue the cilium defect in D12 cells, suggesting that it was not directed to the Rab11+ vesicle population or it lacks the specific proteolytic activity embodied in ADAMTS9 and ADAMTS20. ADAMTS16, which is distantly related to ADAMTS9, was reported to restrict the length of the primary cilium in renal tubular epithelium^53^; however, its loss had no impact on rat morphogenesis, and it is not known to localize to the cilium. Although the present studies unequivocally demonstrate the role of ADAMTS9 and ADAMTS20 in ciliogenesis, they could contribute to neural tube closure and Hh signaling in mice by additional mechanisms. ADAMTS9 heparin-binding is potentially relevant to Shh activation, diffusion or transport, since Shh binds HSPGs and undergoes proteolytic shedding at the cell surface^65,66^. Furthermore, increased proteoglycan in neural epithelium can potentially modify tissue biophysical properties and cell adhesion, each being required for closure of the neural tube^67^. We have not formally tested these possibilities, choosing to specifically address ciliogenesis here.

A key question that arises is whether the observed primary cilium defect is secondary to impaired ECM turnover. The cilium is closely approximated to ECM and carries ECM receptors such as integrins^68^. Altered cell−matrix interactions in the absence of ADAMTS9 influence smooth muscle cell focal adhesion formation, shape, actin cytoskeleton assembly and nuclear morphology via accumulation of pericellular versican^69^. Arguing for a direct role in ciliogenesis, ADAMTS9 and ADAMTS20 localization to peri-centrosomal vesicles is necessary for ciliogenesis. RPE-1 cells express low levels of the ADAMTS substrate versican, and no changes in versican or cell shape (reflecting anomalous cell−matrix interactions) were noted in D12 cells in the 24 h time-frame utilized in our ciliogenesis assays. Furthermore, expression of ADAMTS5 did not rescue ciliogenesis in ADAMTS9-deficient D12 cells, versican overexpression did not affect ciliogenesis in wild-type RPE-1 cells, and the ciliogenesis defect of D12 cells could not be rectified by providing a high concentration of the proadhesive molecule fibronectin. We therefore conclude that although ADAMTS9 and ADAMTS20 unequivocally mediate proteolytic modification of ECM components as a canonical function, they act noncanonically as modulators of ciliogenesis. Major future directions from this work include identification of potential ADAMTS9/ADAMTS20 substrates at either the cell surface or within cilia-bound endocytic vesicles that are crucial for ciliogenesis.

## Methods

### Mice

Mouse experiments were conducted under a Cleveland Clinic Institutional Animal Care and Use Committee (IACUC) approved protocol (2015:1530), in compliance with all relevant ethical regulations. Mice were maintained in a fixed light−dark cycle with ad libitum access to food and water. *Adamts9*^*Gt*^ ^17^, *Adamts9*^Del^ ^45^, *Adamts20*^*Bt*^ ^13^ and *Vcan*^*Hdf*^ ^70^ alleles and their genotyping were previously described. *Ptch1*^*LacZ*^ mice (Stock No. 003081) and *Shh*^*eGFP-Cre*^ mice (Stock No. 005622) were purchased from The Jackson Laboratories and genotyped as instructed.

### Mammalian cell culture

hTERT RPE-1 (CRL-4000; ATCC, Manassas, VA), IMCD-3 (CRL-2123; ATCC, Manassus, VA), and NIH-3T3 cells (CRL-1658; ATCC, Manassus, VA) were cultured in DMEM F12 with 10% FBS, 100 U/mL penicillin/streptomycin under 5% CO_2_. RPE-1 growth medium was supplemented with 0.01 mg/mL hygromycin B. Human neonatal foreskin dermal fibroblasts (HDFs) were purchased from Lonza (CC-2509, Basel, Switzerland) and cultured in DMEM with 10% FBS, 100 U/mL penicillin/streptomycin under 5% CO_2_.

### Plasmids and transfection

Expression plasmids for human ADAMTS9, mouse ADAMTS20, and their corresponding inactive mutants (ADAMTS9 E/A and ADAMTS20 E/A) were previously described^12,20^ as were ADAMTS1, ADAMTS5, ADAMTS16 and TIMP3 constructs^71–74^. Three hundred nanograms of each construct was transfected using Lipofectamine 3000 reagent (Invitrogen, catalog no. L3000-015) in eight-well chamber slides. Fifty nanograms of Vcan-V1 plasmid^75^ was transfected for over expressing versican in RPE-1 cells.

### CRISPR/Cas9 mutagenesis

We used custom polycistronic plasmids encoding guide RNAs targeting exon 2 of *ADAMTS9*, Cas9 and GFP or RFP (Sigma-Aldrich, Cat. No. 03211705MN, 03211707MN). For establishing mutant cell lines, hTERT-RPE-1 cells were cultured in six-well plates and transfected with 2.5 μg of plasmid DNA using Lipofectamine 3000 reagent (Invitrogen, Cat. No. L3000-015). Transfected cells were trypsinized after 24 h and GFP or RFP-positive cells were sorted into 96-well plates using- the FACSAria-II cell sorter for clonal growth (BD Biosciences). Wells containing >1000 cells/clone were trypsinized and expanded into 24-well plates after 1 week. Genomic DNA was harvested from the established clones using the DirectPCR (Tail) digest reagent (Viagen, Cat. No. 102-T) and exon 2 of *ADAMTS9* was PCR amplified using the proof-reading Phusion high-fidelity polymerase (NEB, Cat. No. F530L). Amplicons were excised from 2% agarose gels, purified using the QIAquick Gel Extraction kit (QIAGEN, Cat. No. 28704), cloned in pCR-Blunt II-TOPO vector using the Zero blunt PCR cloning kit (Life Technologies, Invitrogen, Cat. No. K2800-40) and transformed into One Shot TOP10 chemically competent *E. coli* (Invitrogen, Cat. No. C404006). At least ten bacterial colonies grown on LB agar plates containing 50 μg/mL kanamycin were picked (corresponding to a single 96-well RPE-1 cell colony). Plasmid DNA was harvested using the QIAprep spin mini-prep kit and the inserted amplicons were Sanger-sequenced using the SP6 primer. hTERT-RPE1 clones with 50% disrupted and 50% wild-type sequences were considered heterozygous (clone RB4), whereas those with 100% mutant sequences were identified as homozygous mutants (D12). Lack of ADAMTS9 was confirmed by western blotting and immunostaining.

### Immunostaining and fluorescence microscopy

Immunostaining of mouse tissue was carried out on 30 μm-thick vibratome sections^17^, or on paraffin-embedded 7 μm sections. Immunostaining of cultured cell lines were carried out in four-chamber or eight-chamber cell culture slides. High-resolution fluorescent images of mouse embryo sections were acquired using a Leica TCS SP5 II multiphoton confocal microscope equipped with a ×25 water immersion objective (Leica Microsystems, Wetzlar, Germany) or using an Olympus BX51 upright microscope (Olympus, Center Valley, PA) equipped with a Leica DFC7000T camera and Leica Application Suite v4.6 imaging software (Leica Microsystems, Wetzlar, Germany). Details of all antibodies used are provided in Supplementary Table 1.

### Deconvolution super-resolution confocal microscopy (DSCM)

A Leica TCS SP8 confocal microscope (Leica Microsystems, Wetzlar, Germany) upgraded with the Hyvolution-2 modification was used for super-resolution microscopy. In brief, confocal images were acquired utilizing the HyD hybrid detectors, and the Leica LAS X operating software (version 3.1.5.16308) and simultaneously deconvolved using the Huygens HyVolution-2 plug-in (Scientific volume Imaging, Hilversum, Netherlands). Hyvolution-2 combines multiparameter fluorescence imaging with point spread function-based real deconvolution, allowing for high-speed multicolor super-resolution imaging with a maximum resolution of 140 nm. ×1000 super-resolution images of primary cilia or centrosomes in RPE-1 cells were acquired using an HCX PL Apo ×100 1.47NA oil immersion objective and a ×10 zoom. Cells were cultured in Falcon 4-chamber slides (Fisher scientific, Cat. No. 354114,) and mounted in ProLong Gold Antifade mountant containing DAPI (Cell Signaling, Cat. No. 8961S). For 3D-projection of deconvoluted Z-stacks, the Volocity 3D imaging software (version 6.3, PerkinElmer, Inc., Waltham, MA) 3D-opacity projection method was used.

### Optical projection tomography

Fixed embryos were embedded in low melting point agarose (IBI scientific, Cat. No IB70056) (1.1% in Milli-Q water), the agarose blocks trimmed and then superglued to magnetic stubs. The embedded tissue was then placed in 100% methanol for 24 h, which was replaced twice (each for a further 24 h), to ensure complete dehydration. The samples were submerged in one part benzyl alcohol to two parts benzyl benzoate for 24 h and again the solution replaced twice for 24 h each to ensure complete optical clearing of the tissue. Cleared samples were subjected to imaging in a Bioptonics 3001M optical Projection Tomograph in the Small Animal Tomographic Analysis (SANTA) Facility at the Seattle Children’s Research Institute. The embryos were imaged at ~12.6 μm resolution using a 1024 × 1024 image format: 88 ms exposure, three-frame averaging. Imaging was conducted under UV light using a GFP1 filter (425 nm excitation, 475 nm emission) to detect tissue autofluorescence. Raw imaging data (TIF format) were reconstructed into multidimensional slice data (BMP format) using NRecon v1.6 software (Skyscan, Belgium). Reconstructed data were then analyzed both in 2D multiplanar format in Dataviewer (Skyscan, Belgium) and in 3D following rendering in the Drishti v2.6 Volume Exploration software (http://sf.anu.edu.au/Vizlab/drishti).

### Electron microscopy and immunogold electron microscopy

For SEM imaging of neural tube or yolk sac cilia, embryos were harvested from timed pregnancies at 9.5 or at 12.5 days of gestation and fixed in 4% paraformaldehyde (PFA) + 2.5% glutaraldehyde in PBS overnight. To visualize primary cilia in wild-type neural tube, the cranial vault was de-roofed after fixation. Samples were post-fixed in 1% osmium tetroxide for 1 h, washed in deionized sterile water and dehydrated in an ethanol gradient. Samples were immersed in a 1:1 mixture of 100% EtOH:100% hexamethyldisilazane (HMDS) overnight, washed three times in 100% HMDS and air dried for 48 h. Dehydrated samples were mounted on standard aluminum specimen mounts utilizing double-sided adhesive tape with the open neural tube facing up. Samples were coated with gold to a 15 nm thickness using an EMS1550TES sputter coater (Electron Microscopy Sciences, Hatfield, PA), and imaged using a JEOL JSM5310 Scanning Electron Microscope, (JEOL, Inc. Peabody, MA) equipped with an Orion image acquisition and handling system image-grabber (Model p13315A, JEOL, Inc. Peabody, MA). For imaging primary cilia of the yolk sac mesothelium, dissected yolk sacs were flat-mounted with the mesothelial side facing up prior to fixation.

Immunogold labeling for ADAMTS9 in RPE-1 cells was done using the pre-embedding technique. RPE-1 cells were plated in two-chamber Permanox slides (Lab-Tek, catalog no. 177429) and cultured to confluency before serum starvation for 48 h. Cells were washed in PBS and fixed in 4% PFA in PBST for 20 min. Cells were washed and permeabilized with PBST for 30 min before blocking in 5% normal goat serum for 1 h and overnight incubation with or without primary antibody against human ADAMTS9 (linker-2) at a 1:600 dilution at 4 °C. Samples were washed in PBST for 30 min and incubated with blocking buffer (0.1% BSA, 0.1% fish gelatin, and 0.2% Tween20 in PBS) for an additional 30 min. Goat anti-rabbit 5 nm gold-conjugated secondary antibody (EMS, catalog no. 15725) was diluted at 1:50 in blocking buffer and incubated for 4 h at room temperature. Samples were washed in blocking buffer for 30 min and fixed overnight in 2.5% glutaraldehyde, 4% PFA in 0.2 M cacodylate buffer (pH 7.3). Dehydrated samples were embedded in pure Eponate12 resin and polymerized overnight at room temperature. Eighty-five nanometers ultrathin sections were collected and stained with uranyl acetate and lead citrate and imaged using a FEI Tecnai G2 Spirit BioTWIN Transmission Electron Microscope (FEI company, Hillsboro, OR) equipped with an Orius 832 CCD camera (Gatan, Inc., Pleasanton, CA).

### RNAscope in-situ hybridization

E8.5-E11.5 mouse embryos were fixed in 4% PFA prior to paraffin embedding. Seven micrometer-thick sections were collected immediately prior to in-situ hybridization experiments using RNAscope probes specific for mouse *Adamts9* (ACD, Cat. No. 400401), *Adamts20* (ACD, Cat. No. 400541), *Vcan* exon 7 (GAG-α domain) (ACD, Cat. No. 428311), *Vcan* exon 8 (GAG-β domain) (ACD, Cat. No. 428321), *Shh* (ACD, Cat. No. 314361), *Ptch1* (ACD, Cat. No. 402811), *Gli-1* (ACD, Cat. No. 311001), *Gli-2* (ACD, Cat. No. 405771), *Bmp4* (ACD, Cat. No. 401301), *Noggin* (ACD. Cat. No. 467391). Hybridization was done using a HybEZ oven and detected using the 2.5 HD Red detection kit. Hematoxylin was used as the counterstain. Sections were photographed on an Olympus BX51 upright microscope (Olympus, Center Valley, PA) using a Leica DFC7000T camera and Leica Application Suite v4.6 imaging software (both from Leica, Wetzlar, Germany).

### Transferrin uptake assay

hTERT-RPE-1 cells grown to confluence in eight-chamber cell culture slides and serum-starved for 24 h were cooled on ice for 10 min and the culture medium was switched to ice-cold serum-free medium for 5 min to block endocytosis. Alexa Fluor647-conjugated transferrin (Life Technologies, catalog no. T2366) was diluted in serum-free culture medium to a final concentration of 0.25 mg/mL and was prewarmed to 37 °C before adding to the cells. Cells were incubated with medium containing Alexa Fluor647-transferrin at 37 °C for 30 min, washed thrice with serum-free medium, fixed in 4% PFA for 15 min, washed and costained for ADAMTS9 and acetylated α-tubulin and imaged using super-resolution confocal microscopy at ×1000 magnification.

### Inhibition of endocytosis

hTERT-RPE-1 cells were grown to confluence in four-chamber cell culture slides and cultured in serum-free medium for 24 h to induce ciliogenesis. Dyngo4A (Abcam, catalog no. ab120689) and Pitstop 2 (Abcam, catalog no. ab120687) were dissolved in DMSO as 100 mM stock solutions. Serum-free culture medium containing 20 μM Dyngo4a or 10 μM Pitstop-2 was prewarmed to 37 °C and added to RPE-1 cells for 4–8 h. Serum-free culture medium containing 0.2% DMSO was used as a vehicle control. After incubation, cells were washed thrice in fresh serum-free culture medium, fixed in 4% PFA diluted in PBS containing 0.1% Tween-20 (PBST), for 20 min and stained for acetylated α-tubulin and ADAMTS9.

hTERT-RPE-1 cells grown to 80% confluence in four-chamber culture slides were transfected with Silencer® Select predesigned and validated siRNA targeting human CHC (s477, Ambion catalog no. 4390824), LRP-1 (106762, Ambion catalog no. AM51331) and LRP-2 (143880, Ambion catalog no. AM16708), or Silencer select negative control siRNA-2 (Ambion catalog no. 4390846) using Lipofectamine RNAiMAX reagent (Invitrogen, catalog no. 13778075). Cells were incubated with the transfection mix for 48 h for complete depletion of the target protein and washed in PBS and cultured for additional 24 h in serum-free medium for ciliogenesis. Treated cells were fixed in 4% paraformaldehyde in PBS containing 0.1% Tween 20 (PBST) for 20 min and stained for acetylated α-tubulin and ADAMTS9.

### SAG treatment of RPE-1 cells

Wild-type or D12 hTERT-RPE-1 cells were grown to confluence in eight-chamber cell culture slides for immunostaining, or six-well culture plates for RNA extraction, and cultured in serum-free medium for 24 h to induce ciliogenesis. Serum-free culture medium containing 500 nM InSolution Smoothened Agonist (SAG) (EMD Milipore, Cat. # 566661) was added to cells for 12 h. Cells cultured in chamber slides were washed in PBS and fixed in 4% PFA for analysis by immunostaining and cells cultured in six-well plates were washed in PBS and RNA was extracted using TRIzol RNA extraction reagent (Thermo Fisher, Cat. # 15596026).

### Isolation of HS and HS coupling to HiTrap columns for FPLC

HS columns were generated as follows: 1 g (wet weight) C57/BL6 mouse embryos (E12-E18) were homogenized and digested in 320 mM NaCl, 100 mM sodium acetate (pH 5.5) containing 1 mg/mL pronase, and 1 mg/mL proteinase K for 72 h at 40 °C. Fresh enzymes were added every 12 h. The digested samples were centrifuged, filtered, diluted 1:3 in water and 2.5 mL aliquots were applied to DEAE Sephacel columns. Eluted glycosaminoglycans were lyophilized, diluted, and quantified by the carbazole reaction. The material was then coupled to NHS-activated sepharose columns. The columns were tested using recombinant HS-binding proteins fibroblast growth factors 2 and 8, vascular endothelial growth factor and Semaphorin 3F as positive controls. FPLC was conducted on an Äkta protein purifier (GE Healthcare) at 8 °C. Samples were applied to the columns in the absence of salt, and bound material was eluted by using a linear 0 to 1.5 M NaCl gradient in 0.1 M phosphate buffer (pH 7.0). Eluted fractions were TCA precipitated and analyzed by SDS-PAGE. Signals were quantified by using ImageJ. For heparin affinity chromatography, commercial heparin Sepharose columns (GE Healthcare) were used.

### Western blotting

SDS-PAGE was carried out on 7.5% acrylamide gels or 4–20% gradient gels under reducing conditions. Proteins were transferred to Immobilon-FL transfer membranes (EMD Milipore, cat. no. IPFL00010) and blocked using 5% nonfat dry milk made in PBST. Primary antibodies were diluted in blocking buffer and incubated overnight at 4 °C. LI-COR IR dye secondary antibodies (LI-COR Biosciences, Lincoln, NE), goat anti-mouse IR dye 800CW (Catalog no. 926-32210), or goat anti-rabbit IR dye 680RD (Catalog no. 926-68071) were used at 1:10,000 dilutions in PBS to detect primary antibodies. Uncropped images of all blots can be found in Supplementary Fig. 8.

### Western blot band intensity quantifications

A LI-COR Odyssey CLx scanner (LI-COR Biosciences, Lincoln, NE) operated using LI-COR Image Studio (ver. 4.0) was used to scan antibody-stained western blot membranes and for quantification of band intensities. Experimental band intensities were normalized to GAPDH intensity measured in each lane. Statistical significance of normalized band intensities was analyzed and graphed using the GraphPad Prism software (La Jolla, CA). Statistical significance was determined by an unpaired two-tailed Student’s *t* test.

### Fluorescent microscopy image quantifications

For quantifying vesicle diameters and distances, super-resolution confocal microscopy images obtained at ×1000 optical magnification were measured by the Leica LAS X software (version 3.1.5.16308) distance measurement function as shown in Fig. 1j. For quantifying ADAMTS9 pixel intensities at the base of the cilium, conventional confocal microscopy images (nondeconvoluted) were analyzed by ImageJ. In brief, a boxed region of interest (ROI) was drawn directly underneath the axoneme and transferred to the corresponding ADAMTS9 channel (red/568 channel), and the arbitrary mean pixel intensity for the ROI was measured. The “restore selection” function was used to transfer the same ROI to all images analyzed. To determine primary cilium length in cultured cells, the freehand distance measurement tool in ImageJ (NIH) was used to measure the acetylated α-tubulin staining signal of fluorescent images taken at ×100 magnification. To determine primary cilium length in mouse neural tubes and yolk sacs the freehand distance measurement tool in ImageJ was used to trace the primary cilium in SEM images. The GraphPad Prism software (La Jolla, CA) was used to determine statistical significance using an unpaired two-tailed Student’s *t* test.

## Supplementary information

Supplementary Information

Peer Review File

Description of Additional Supplementary Files

Supplementary Movie 1

Supplementary Movie 2

Supplementary Movie 3

Supplementary Movie 4

Supplementary Movie 5

Supplementary Movie 6

Reporting Summary

## Data Availability

The data that support the findings of this study are available from the Lead Contact, Dr. Suneel Apte (aptes@ccf.org). Requests for resources and reagents should also be directed to the Lead Contact, Dr. Suneel Apte (aptes@ccf.org).
