# Supplementary material for: Secreted metalloproteases ADAMTS9 and ADAMTS20 have a non-canonical role in ciliary vesicle growth during ciliogenesis
Source: Nat Commun. 2019 Feb 27;10:953. doi: 10.1038/s41467-019-08520-7 (PMC6393521; doi:10.1038/s41467-019-08520-7)
Supplement: Supplementary file 1 — Supplementary Information [file 41467_2019_8520_MOESM1_ESM.pdf]

Nandadasa et al

Secreted metalloproteases ADAMTS9 and ADAMTS20 have a non-canonical role in ciliary vesicle growth during ciliogenesis

SUPPLEMENTARY INFORMATION

Supplemental Figures 1-8

Supplemental Table 1

Supplemental Figure 1, Related to Figure 1.

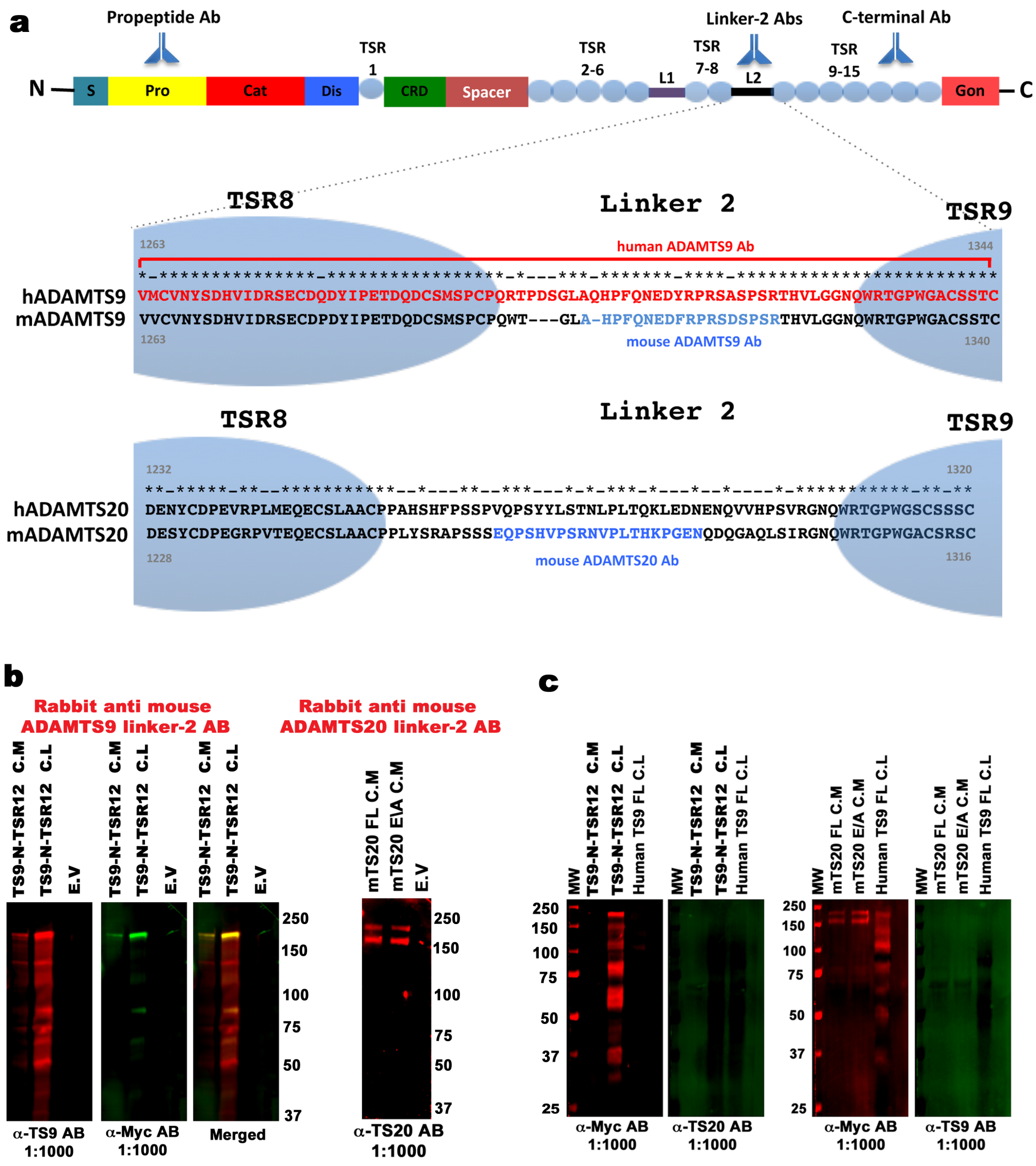

Supplemental Figure 1, Related to Figure 1. Generation and validation of primary antibodies against human and mouse ADAMTS9 and ADAMTS20.

(a) Domain structure (top) of ADAMTS9 and ADAMTS20 with the locations of antibody epitopes in linker 2 (L2) and the C-terminal region indicated. The linker 2 immunogenic peptides (bottom) are indicated by the red bracket over aligned human (red) and mouse (blue) ADAMTS9 and

ADAMTS20 sequences. The asterisks indicate identical residues. Truncated blue ovals show the location of thrombospondin type 1 repeats flanking L2.

(b) Validation of rabbit anti-mouse ADAMTS9 and ADAMTS20 linker-2 antibodies by western blotting of cell lysates and conditioned medium of HEK293 cells transfected with human ADAMTS9 N-TSR12-Myc or mouse ADAMTS20-myc.

(c) Western blotting using the mouse ADAMTS9 and ADAMTS20 linker-2 antibodies shows lack of cross reactivity of the antibodies with ADAMTS20 or ADAMTS9 respectively.

Supplemental Figure 2, Related to Figure 1

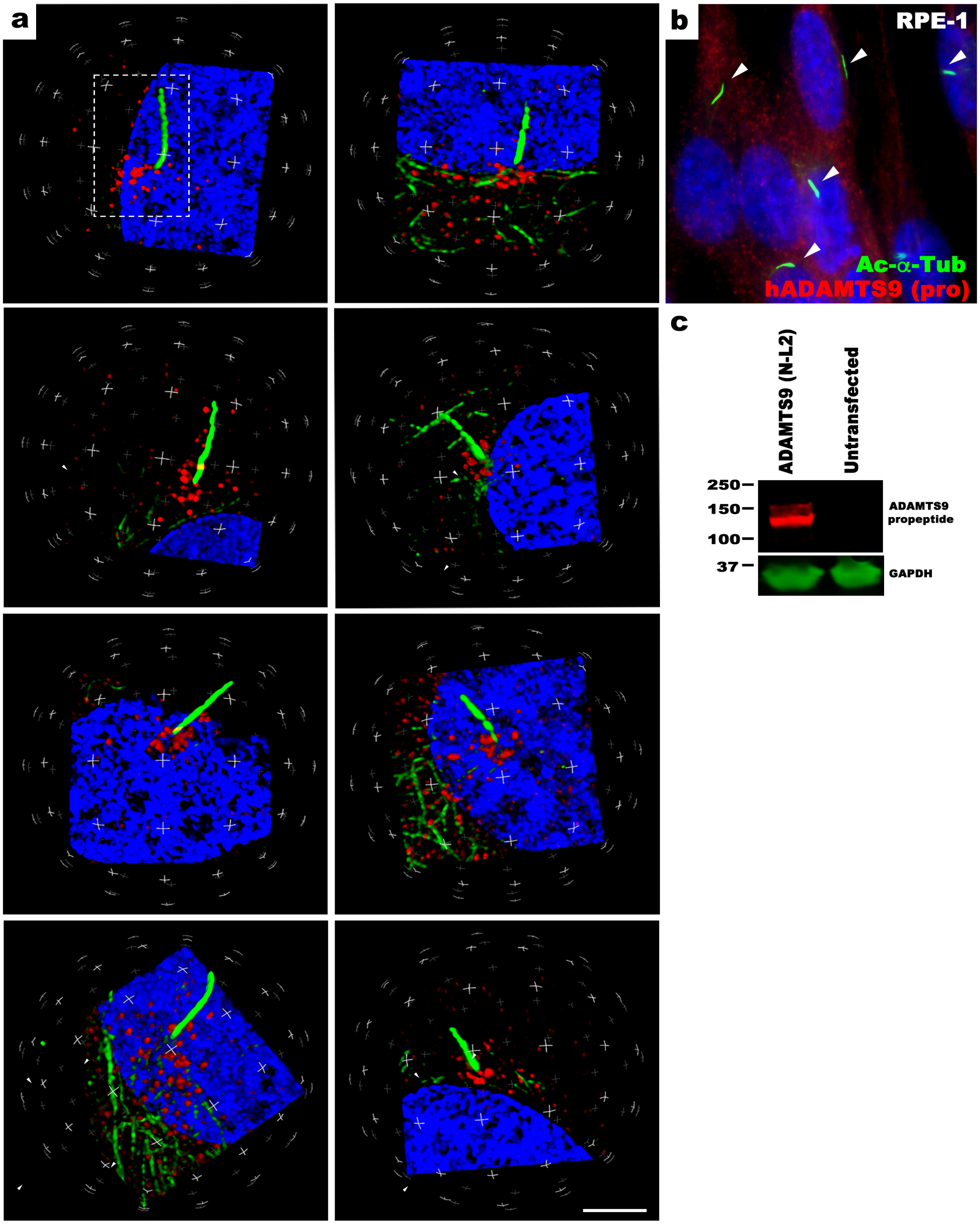

Supplemental Figure 2, Related to Figure 1: Super-resolution imaging of ADAMTS9+ vesicles.

(a) Eight examples of 3D-projections of super-resolution confocal microscopy Z-stacks of RPE-1 cells imaged at 1000X magnification used for determining the diameter of ADAMTS9+ vesicles

and their distance from the base of the primary cilium. Boxed area indicates panel shown in main **Figure-1i.**

(b) Immunostaining of RPE-1 cells with an antibody against the human ADAMTS9 propeptide (red) does not detect periciliary localization although abundant cytoplasmic and membrane staining are seen. White arrowheads indicate primary cilia stained with anti acetylated α-tubulin (green).

(c) Western blot of cell lysates showing that the ADAMTS9 propeptide antibody specifically recognizes human ADAMTS9 zymogen overexpressed in HEK293 cells.

Supplemental Figure 3, Related to Figures 2,3

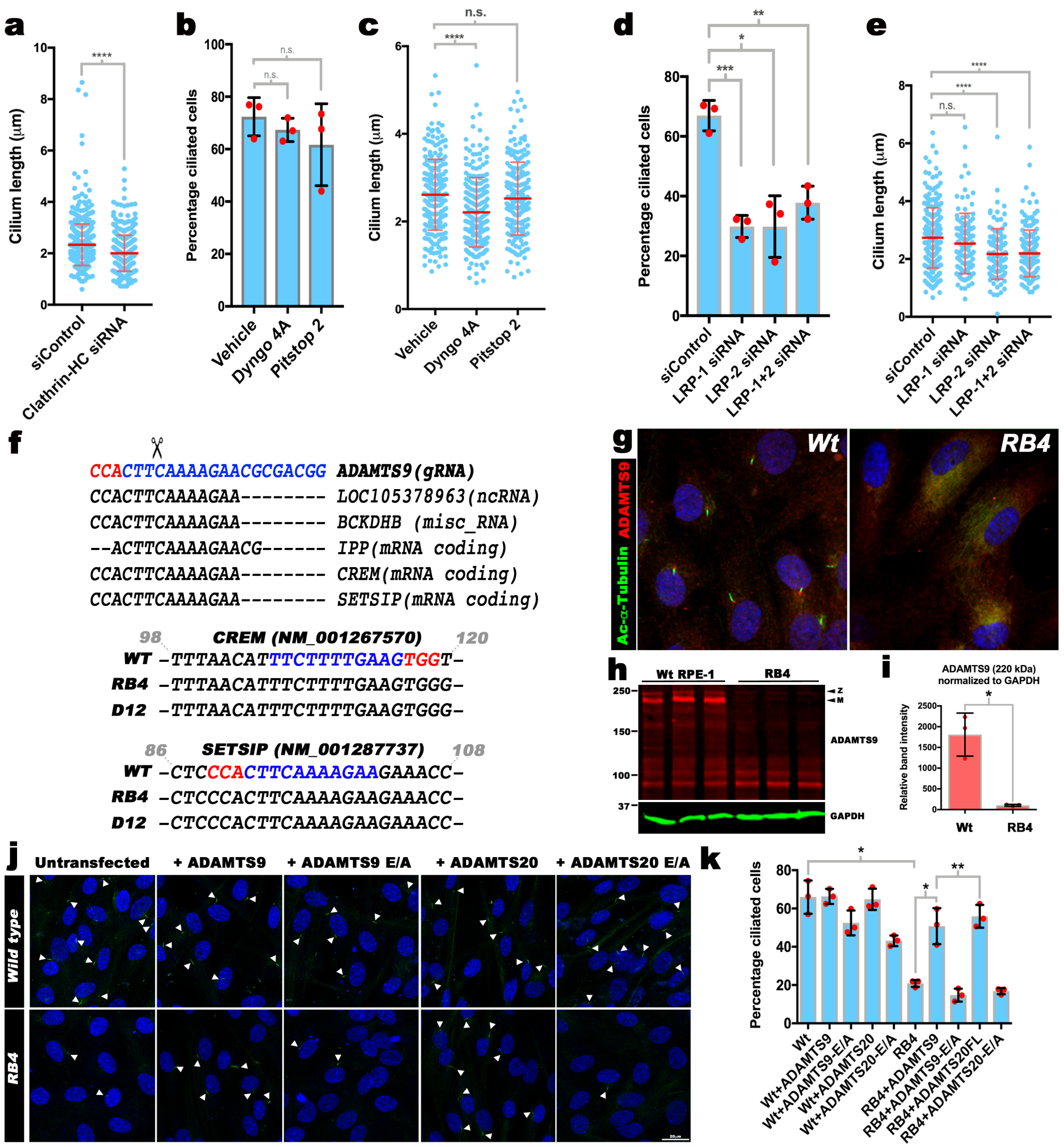

Supplemental Figure 3, Related to Figures 2,3: Quantification of cilia in endocytosis blocking experiments and following CRISPR/Cas9 mutagenesis of ADAMTS9 in RPE-1 cells.

(a) Quantification of cilium length in siControl or clathrin-HC siRNA treated cells (N=3 experiments, ****, p<0.00001).

(b,c) Quantification of the percentage of ciliated cells **(b)** and cilium length **(c)** after the treatment of vehicle or Dyngo 4A and Pitsop 2 endocytosis inhibitors (N=3 experiments, ****, p<0.00001).

(d,e) Quantification of the percentage of ciliated cells **(d)** and cilium length **(e)** of cells transfected with control siRNA, LRP-1, LRP-2 or LRP-1 and LRP-2 siRNAs together (N=3 experiments, *, p<0.05; ***, p<0.0001; **, p<0.001; ****, p<0.00001).

(f) Analysis of the top 5 genes having homologous sequences to the gRNA sequence (potential off-targets). The *ADAMTS9* gRNA sequence is shown in blue and the PAM sequence is shown in red. The noncoding and miscellaneous RNA sequences, and the non-homology at the PAM sequence was used to eliminate the top 3 sequences from further analysis. PCR amplification, followed by Sanger sequencing of *CREM* (transcript variant 31), and *SETSIP* showed no off-target in RB4 and D12 cell lines.

(g) Wild type and RB4 ($ADAMTS9^{+/-}$) RPE-1 cells stained with acetylated α-tubulin (green) and ADAMTS9 (red) show lack of primary cilia and ADAMTS9 staining in the mutant cells.

(h-i) Western blot and quantitation of the 220 kDa ADAMTS9 band shows near-complete loss of ADAMTS9 production in RB4 cells (N=3 experiments, *, p<0.05).

(j-k) Ciliogenesis is rescued in RB4 cells by transfection of full-length ADAMTS9 or ADAMTS20 but not by their catalytically inactive (E/A) forms. Panel **(k)** indicates the percentage of ciliated cells (N=3 experiments, **, p<0.001; *, p<0.05).

Bar charts and dotplots show mean and S.D.

Supplemental Figure 4, Related to Figure 5

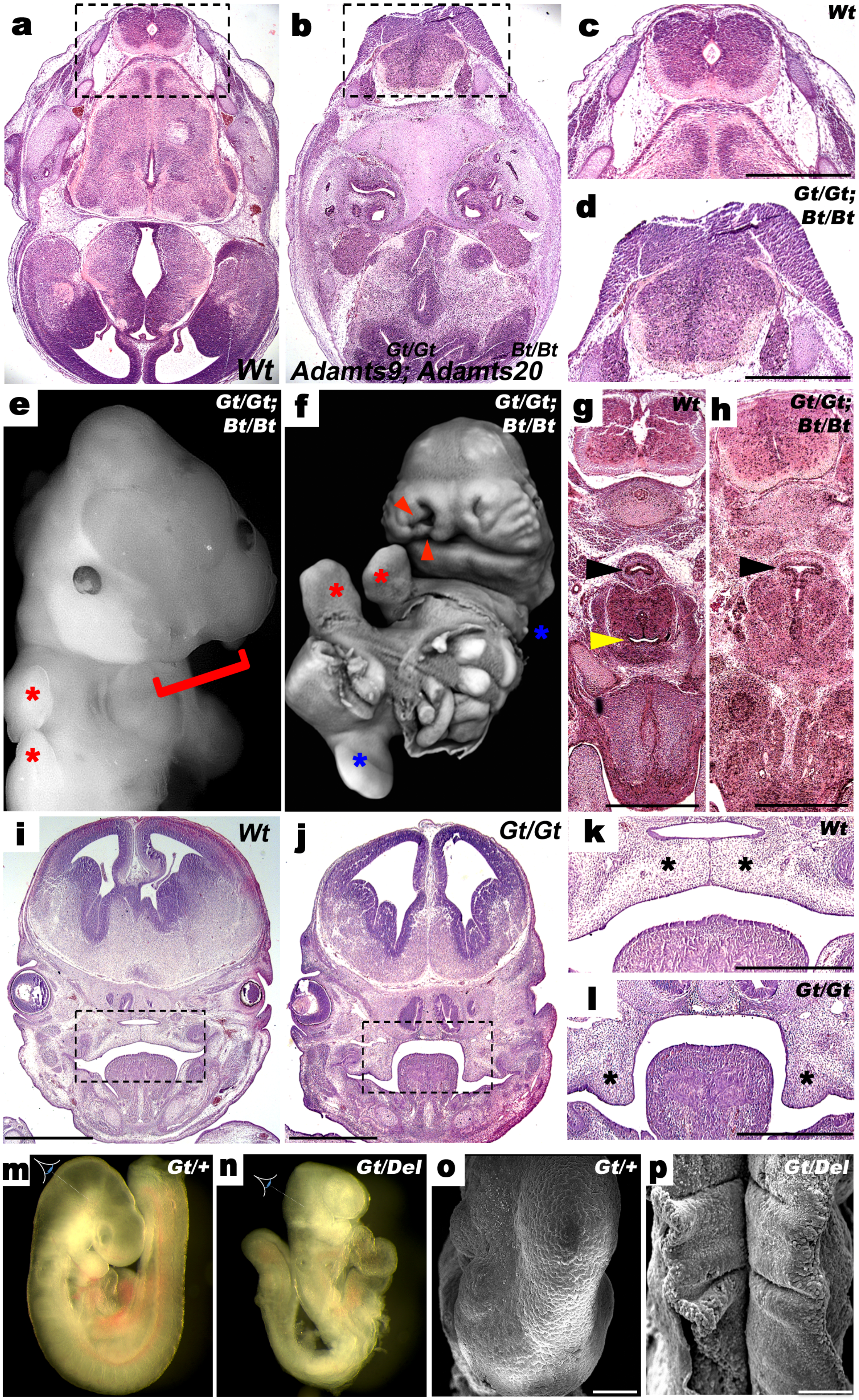

Supplemental Figure 4, Related to Figure 5: Neural tube and craniofacial defects in *Adamts9$^{Gt/Gt}$; Adamts20$^{Bt/Bt}$* embryos.

(a-d) Hematoxylin and Eosin (H&E) stained transverse sections through the heads of E12.5 wild type and *Adamts9$^{Gt/Gt}$; Adamts20$^{Bt/Bt}$* embryos showing exencephaly in the occipital region (boxed) of the mutant. Panels c and d present the boxed areas in panels a and b.

(e) E12.5 *Adamts9$^{Gt/Gt}$; Adamts20$^{Bt/Bt}$* embryos have severe facial clefting and a shortened right-side torso. Red asterisks mark the abnormally approximated right-side limbs and the red bracket marks the facial cleft.

(f) Optical projection tomography (OPT) image of an E12.5 *Adamts9$^{Gt/Gt}$; Adamts20$^{Bt/Bt}$* embryo showing axial asymmetry and cleft lip (red arrows). Red and blue asterisks mark the right and left limbs respectively.

(g,h) H&E stained section of E12.5 wild type **(e)** and *Adamts9$^{Gt/Gt}$; Adamts20$^{Bt/Bt}$* embryo **(f)** showing defective tracheoesophageal septation in the mutant. Black and yellow arrowheads point to the separated esophagus and trachea in the wild type embryo, whereas they are fused in the mutant (black arrowhead).

(i-l) E14.5 *Adamts9$^{Gt/Gt}$* embryos show failed palatal shelf (asterisks) elevation and fusion resulting in cleft palate.

(m,n) E9.5 *Adamts9$^{Gt/Del}$* embryos fail to undergo rotation.

(o,p) Scanning electron micrographs showing open neural tube in an E9.5 *Adamts9$^{Gt/Del}$* embryo. Scale bars = 500μm in **c,d,g,h**, 1000μm in **i,j**, and 100μm in **k,l,o,p**.

Supplemental Figure 5, Related to Figure 5

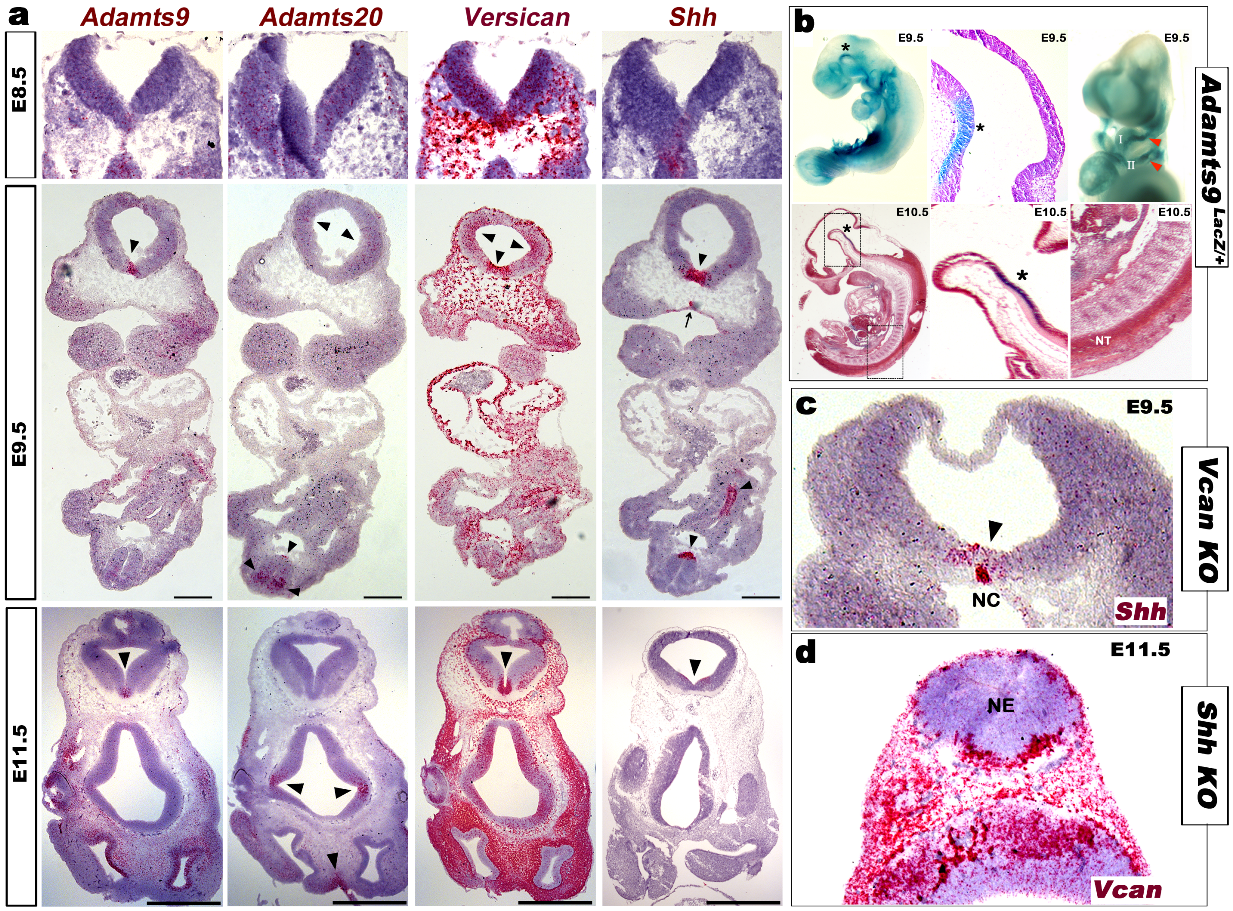

Supplemental Figure 5, Related to Figure 5: *Adamts9, Adamts20, Vcan,* and *Shh* expression during neural tube development.

(a) In-situ hybridization (red signal) of E8.5, E9.5 and E11.5 wild type mouse embryos. During neural tube formation (E8.5 and E9.5) *Adamts9* is expressed in the rostral, but not caudal neural tube floor plate, while *Adamts20* expression is predominantly in the lateral neural epithelium. Strong *Vcan* (GAG β) expression is observed in the rostral floor plate, lateral neural epithelium and adjacent mesenchyme. *Shh* is expressed by the notochord, the rostral and caudal neural tube floor plate and the endoderm at E9.5

(b) β-gal staining of E9.5 and E10.5 *Adamts9*$^{LacZ/+}$ embryos shows *Adamts9* expression is limited to the rostral neural tube floor plate (asterisk). Red arrowheads indicate *Adamts9* expression in the core of the first and second branchial arches. NT indicates the caudal neural tube.

(c) *Shh* expression in the floor plate (arrowhead) is reduced in E9.5 *Vcan*$^{Hdf/Hdf}$ embryos (compare with panel **a,** E9.5), whereas notochordal expression is unaffected.

(d) *Vcan* expression in the neural tube floor plate is lost in E11.5 *Shh*$^{-/-}$ embryos while the expression in the surrounding mesenchymal cells is unaffected (compare with panel **a** E11.5). NE indicates neural epithelium. Scale bars =100μm, and 500μm in **a.**

Supplemental Figure 6, Related to Figures 6,7

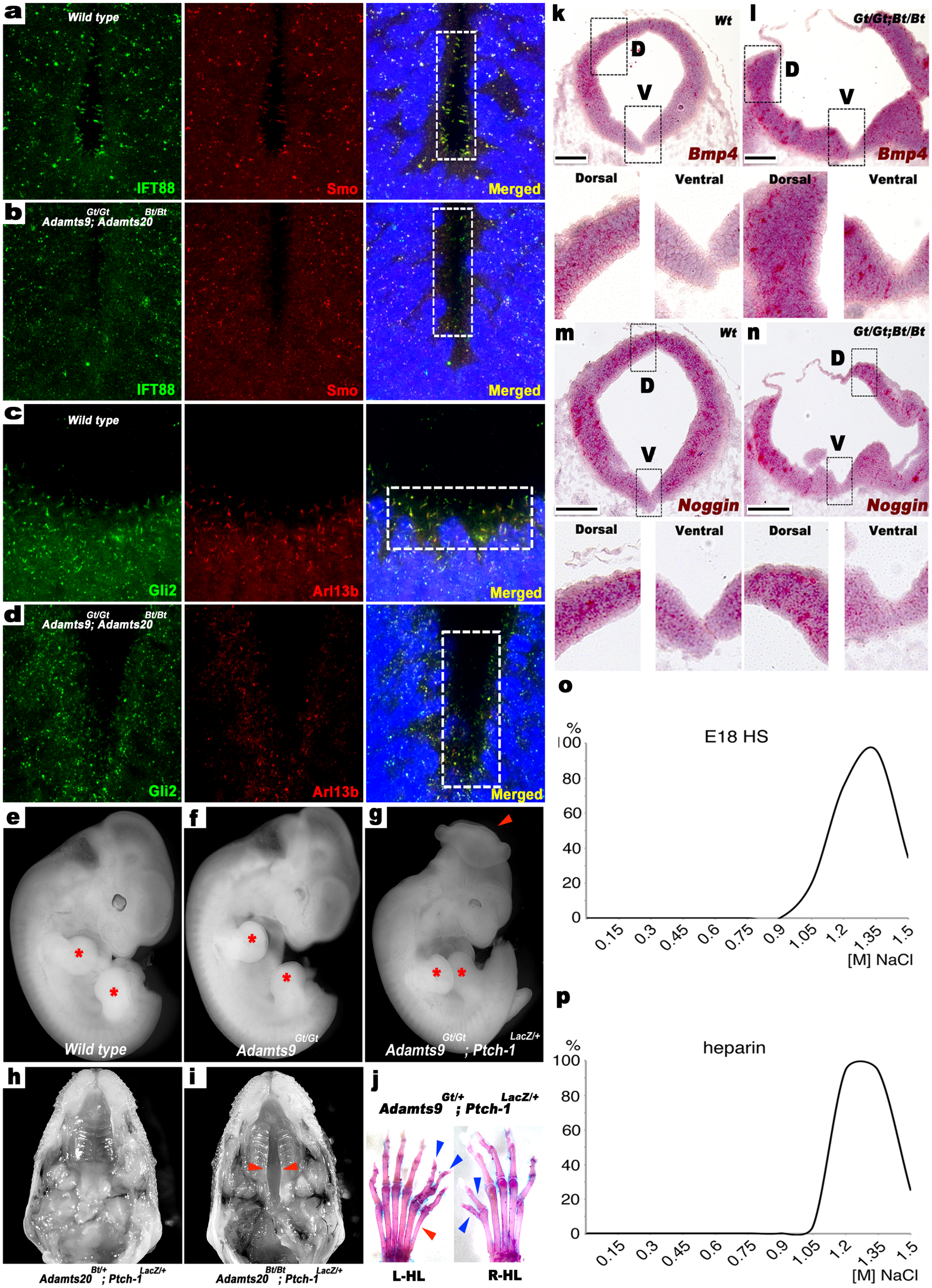

Supplemental Figure 6, Related to Figures 6,7: ECM dynamics, hedgehog genetic interactions and signaling defects in *Adamts9* and *Adamts20* mutants.

(a-b) Immunostaining for ciliary axoneme marker IFT-88 (green), and smoothened (red) shows their co-localization in wild type neural tube and fewer discernable primary cilia in the *Adamts9*$^{Gt/Gt}$*; Adamts20*$^{Bt/Bt}$ neural tubes.

(c-d) Immunostaining with Arl13b (red) and Gli-2 (green) also indicates fewer, short primary cilia and loss of hedgehog signaling in the mutant neural tubes. Higher magnification images of the boxed areas in **a-d** are shown in the main **Figure-6a,b**.

(e-g) Inactivation of one *Ptch1* allele in *Adamts9*$^{Gt/Gt}$ embryos causes fully penetrant occipital exencephaly, shown by a red arrowhead in E11.5 *Adamts9*$^{Gt/Gt}$*; Ptch1*$^{LacZ/+}$ embryos (n=3). Red asterisks show the abnormally approximated right limbs. Both defects were similar to those of *Adamts20*$^{Bt/Bt}$; *Adamts9*$^{Gt/+}$ embryos.

(h-i) Deletion of one *Ptch1* allele in *Adamts20*$^{Bt/Bt}$ embryos causes fully penetrant cleft palate (n=11) shown by the red arrowheads, a phenotype observed in 100% of *Adamts20*$^{Bt/Bt}$; *Adamts9*$^{Gt/+}$ and *Adamts20*$^{Bt/Bt}$; *Adamts9*$^{Del/+}$ embryos.

(j) Alcian blue and Alizarin red stained skeletal preparation from an adult *Adamts9*$^{Gt/+}$*; Ptch-1*$^{LacZ/+}$ mouse showing highly penetrant preaxial polydactyly in hind limbs (HL) (8/11, 72%), greater than the observed penetration of preaxial polydactyly in *Ptch1*$^{LacZ/+}$ mice (<2%). The red arrowhead indicates the extra metatarsal and blue arrowheads indicate duplicated phalanges in digits I (R) and II (L).

(k-l) *Bmp4* in-situ hybridization in E9.5 wild type and *Adamts9*$^{Gt/Gt}$*; Adamts20*$^{Bt/Bt}$ embryos shows increased expression in the dorsal neural tube and ventral expansion of the *Bmp4* domain in the mutant embryos. The boxed fields in the upper panels are shown at higher magnification in the lower panels.

(m-n) *Noggin* expression is comparable in E9.5 *Adamts9*$^{Gt/Gt}$*; Adamts20*$^{Bt/Bt}$ and wild type embryos.

(o-p) Quantification of SDS-PAGE band intensities for ADAMTS9 eluted from fast protein liquid chromatography of heparan sulfate isolated from E18.0 mouse embryos **(o),** or commercial heparin Sepharose column **(p**) using the indicated salt concentrations. Peak ADAMTS9 elution was observed with 1.35 M NaCl, indicative of strong interaction with heparan sulfate and heparin. Scale bars =100μm in **k-n**.

Supplemental Figure 7, Related to Figure 9

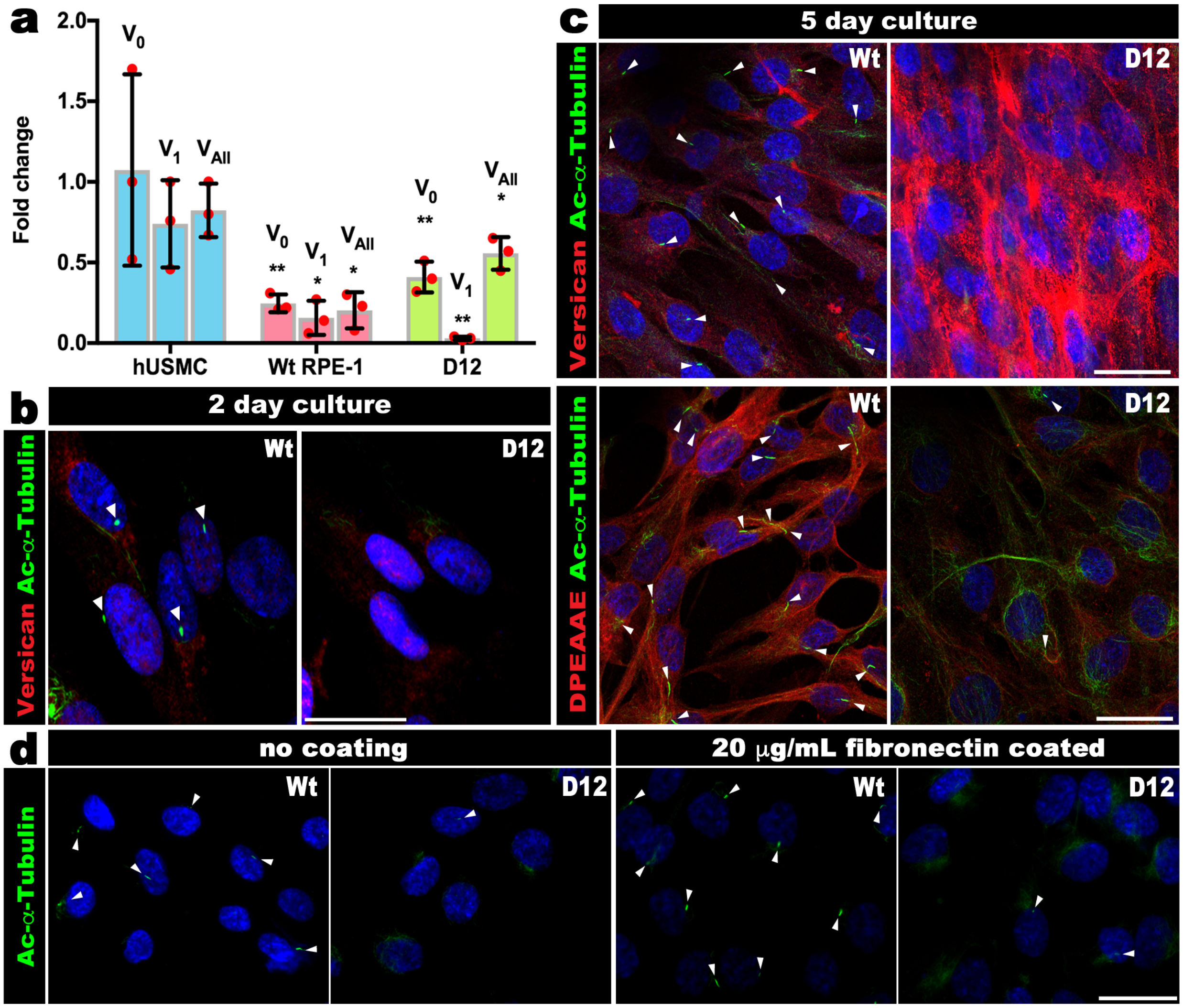

Supplemental Figure 7, Related to Figure 9: Versican levels and culture of RPE-1 cells in fibronectin coated wells.

(a) qRT-PCR for *VCAN* isoforms shows significantly lower levels of expression in wild type or mutant RPE-1 cells compared to human uterine smooth muscle cells (N=3 experiments, .

(b,c) Immunostaining for versican (red) and acetylated α-tubulin (green) shows minimal versican immunofluorescence in RPE-1 cells cultured for 2 days **(b)** and detectable levels of versican accumulation and loss of versican cleavage in mutant cells cultured for 5 days **(c)** (N=3 experiments). **(d)** Acetylated α-tubulin staining (green) of wild type and D12 RPE-1 cells cultured in 20 μg/mL fibronectin coated wells showing no effect on primary cilia (white arrowheads) in wild type cells or D12 cells. Scale bars =20μm in **b, c, d.** In the box and whisker

plot, whiskers indicate the range and boxes mark upper and lower quartiles. The center-line indicate the mean.

Supplemental Figure 8

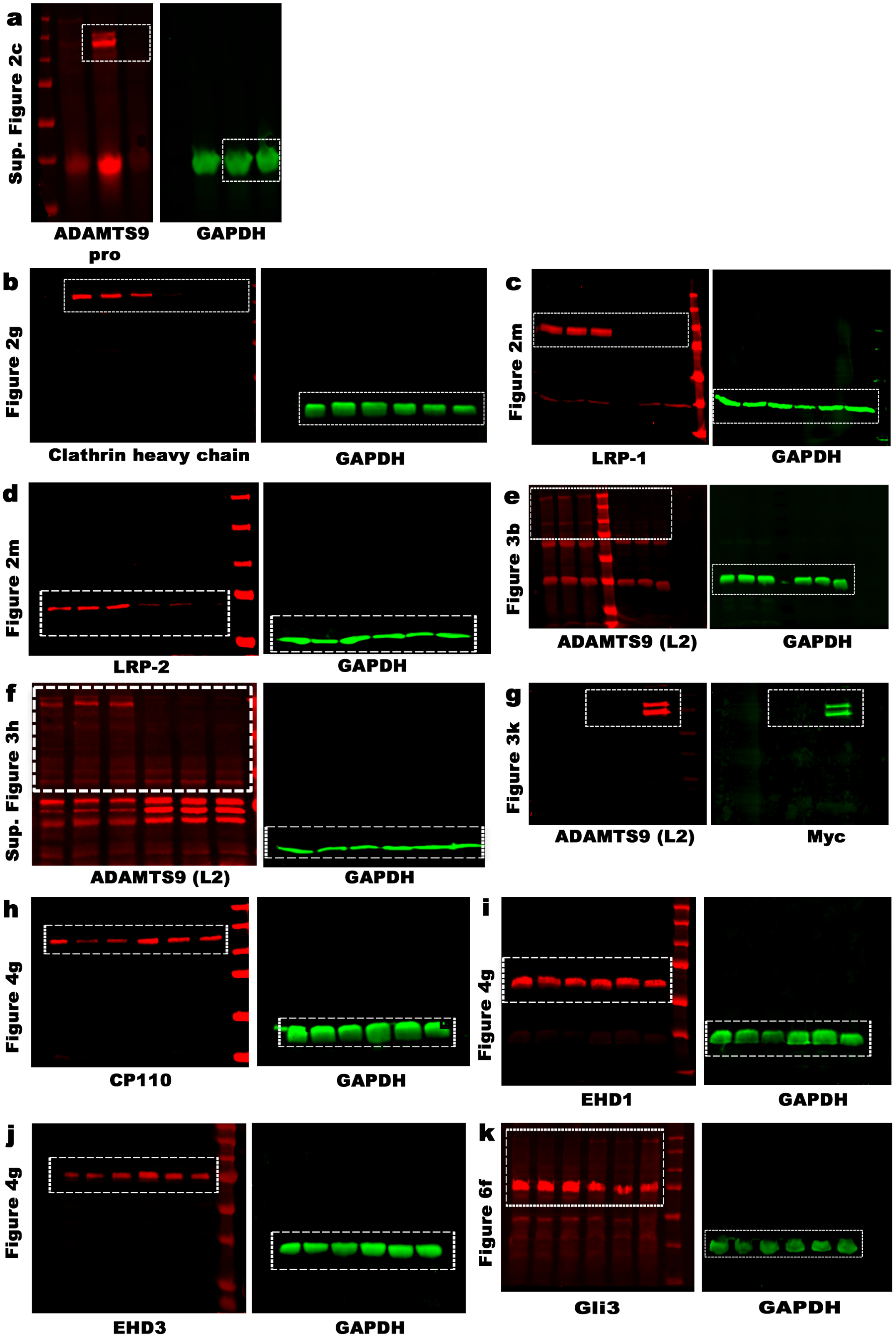

Supplemental Figure 8: Complete unprocessed western blots used in this study.

(a-k) White boxes indicate regions used in main and supplemental figures.

Supplementary Table 1

Antibody	Source	Catalogue No.	Dilution
Rabbit polyclonal human ADAMTS9 (Linker 2)	Sigma-Aldrich	HPA028567<br>Lot: R28204	1:600 (IF)<br>1:1000 (WB)<br>1:600 (I-EM)
Rabbit polyclonal human ADAMTS9 (Propeptide)	Sigma-Aldrich	HPA028577<br>Lot: R27248	1:400 (IF)<br>1:1000 (WB)
Rabbit polyclonal mouse ADAMTS9 (Linker 2)	Apte laboratory	N/A	1:500 (IF)<br>1:1000 (WB)
Rabbit polyclonal mouse ADAMTS20 (Linker 2)	Apte laboratory	N/A	1:500 (IF)<br>1:1000 (WB)
Rabbit polyclonal human ADAMTS20 (C-terminal)	Sigma-Aldrich	HPA027609<br>Lot: R28017	1:600 (IF)
Mouse monoclonal acetylated α-Tubulin (6-11B-1)	Invitrogen	32-2700	1:400 (IF)
Rat monoclonal Alexa-488 Tubulin	Abcam	Ab195883	1:200 (IF)
Mouse monoclonal γ-Tubulin (GTU-88)	Sigma-Aldrich	T6557	1:500 (IF)
Mouse monoclonal Shh (5E1)	DSHB	5E1-b	1:100 (IF)
Rat monoclonal Patched1	R&D Systems	MAB41051	1:100 (IF)
Mouse monoclonal Olig2 (211F1.1)	Millipore-Sigma	MABN50	1:200 (IF)
Mouse monoclonal Nkx6.1	DSHB	F55A10-c	1:200 (IF)
Mouse monoclonal Pax6	DSHB	PAX6-b	1:100 (IF)
Mouse monoclonal Pax7	DSHB	PAX7-c	1:100 (IF)
Mouse monoclonal FOXA2	DSHB	4C7-c	1:50 (IF)
Mouse monoclonal Nkx2.2	DSHB	74.5A5-c	1:50 (IF)
Rabbit polyclonal smoothened	Dr. Annabel Christ	N/A	1:400 (IF)
Rabbit polyclonal Arl13b	Abcam	Ab83879	1:200 (IF)
Goat polyclonal IFT88	NOVUS	NB100-2475	1:200 (IF)
Rabbit polyclonal Gli2	Millipore-Sigma	ABN2241	1:200 (IF)
Rabbit polyclonal Gli3	NOVUS	NBP2-29627	1:1000 (WB)
Mouse monoclonal CEP170	Life Technologies	413200	1:400 (IF)
Mouse monoclonal CEP164	Sigma-Aldrich	SAB2702133	1:200 (IF)
Rabbit polyclonal CEP164	Invitrogen	PA5-58034	1:400 (IF)
Rabbit polyclonal clathrin heavy chain	Abcam	Ab21679	1:100 (IF)
Mouse monoclonal clathrin heavy chain (X22)-Alexa647	Invitrogen	MA1065A647	1:100 (IF)
Mouse monoclonal Rab11	BD Transduction Laboratories	610658	1:100 (IF)
Rabbit polyclonal Rab11A	Proteintech	20229-1AP	1:400 (IF)
Rabbit monoclonal LRP-1	Abcam	Ab21679	1:1000 (WB)
Rabbit polyclonal LRP-2	Proteintech	19700-1-AP	1:1000 (WB)
Mouse monoclonal GAPDH	Millipore-Sigma	MAB374	1:2500 (WB)
Rabbit polyclonal GAPDH	NOVUS	NB300-323	1:2500 (WB)
Rabbit polyclonal mouse versican	Millipore-Sigma	AB1033	1:200 (IF)
Rabbit polyclonal versican-Neo (DPEAAE)	Thermo Fisher Scientific	PA1-1748A	1:100 (IF)
Rabbit polyclonal human versican (pVC)	Apte laboratory	N/A	1:400 (IF)
Rabbit polyclonal fibronectin	Abcam	Ab2413	1:200 (IF)
Rabbit polyclonal laminin	Sigma-Aldrich	L9393	1:400 (IF)

Rabbit polyclonal collagen IV	Rockland antibodies & assays	600-401-106	1:800 (IF)
Mouse monoclonal chondroitin sulfate (7D4 epitope)	Dr. Clare Hughes	N/A	1:200 (IF)
Mouse monoclonal heparan sulfate-FITC (10E4)	US Biological	H1890-10	1:100 (IF)
Rabbit polyclonal phospho-histone-H3 (Ser-10)	Millipore-Sigma	09-797	
Mouse monoclonal c-myc (9E10)	Cleveland Clinic Hybridoma core	N/A	1:400 (IF)<br>1:1000 (WB)
Rabbit polyclonal c-myc	Sigma-Aldrich	C3956	1:400 (IF)<br>1:1000 (WB)
Rabbit polyclonal CP110	Proteintech	12780-1-AP	1:200 (IF)<br>1:1000 (WB)
Rabbit monoclonal EHD1	Abcam	Ab109311	1:1000 (WB)
Mouse monoclonal EHD3	Abnova	H00030845-A01	1:1000 (WB)
Rabbit polyclonal TCTN2	Proteintech	17053-1-AP	1:300 (IF)
Goat anti-rabbit Alexa-488	Thermo Fisher Scientific	A-11034	1:500 (IF)
Goat anti-rabbit Alexa-568	Thermo Fisher Scientific	A-11011	1:500 (IF)
Goat anti-mouse Alexa-488	Thermo Fisher Scientific	A-11029	1:500 (IF)
Goat anti-mouse Alexa-568	Thermo Fisher Scientific	A11004	1:500 (IF)
Goat anti-rat Alexa-647	Thermo Fisher Scientific	A-21247	1:500 (IF)
Donkey anti-goat Alexa-488	Thermo Fisher Scientific	A-11055	1:500 (IF)
Goat anti-rabbit 5 nm gold-conjugated antibody	EMS	15725	1:50 (I-EM)
Goat anti-rabbit IR dye 800CW	LI-COR	926-32211	1:10,000 (WB)
Goat anti-mouse IR dye 800CW	LI-COR	926-32210	1:10,000 (WB)
Goat anti-rabbit IR dye 680RD	LI-COR	926-68071	1:10,000 (WB)
Goat anti-mouse IR dye 680RD	LI-COR	926-68070	1:10,000 (WB)
**Bacterial and Virus Strains**	**Source**	**Catalogue No.**	
E. coli : One Shot TOP10 cells	Invitrogen	C404006	
**Chemicals, Peptides, and Recombinant Proteins**	**Source**	**Catalogue No.**	**Dilution**
Hyaluronan binding protein (HAbp)	Millipore-Sigma (Calbiochem)	385911	1:100 (IF)
Alexa-647 transferrin	Invitrogen	T23366	0.25mg/mL (IF)
Biotinylated isolectin B_4	Vector Laboratories	B-1205	1:400 (IF)
Pitstop2	Abcam	Ab120687	10 μM
Dyngo4A	Abcam	Ab120689	20 μM
InSolution Smoothened Agonist (SAG)	EMD Millipore	566660	500 nM
Human plasma Fibronectin	Corning	354008	20 μg/mL (Cell culture)
TRIzol RNA extraction reagent	Thermo Fisher	15596026	

**Critical Commercial Assays**	**Source**	**Catalogue No.**	
RNAscope mouse *Adamts9 in situ* probe	Advanced Cell Diagnostics	ACD 400401	
RNAscope mouse *Adamts20 in situ* probe	Advanced Cell Diagnostics	ACD 400541	
RNAscope mouse *Vcan* Exon 7 *in situ* probe (GAG α)	Advanced Cell Diagnostics	ACD 428311	
RNAscope mouse *Vcan* Exon 8 *in situ* probe (GAG β)	Advanced Cell Diagnostics	ACD 428321	
RNAscope mouse *Shh in situ* probe	Advanced Cell Diagnostics	ACD 314361	
RNAscope mouse *Ptch1 in situ* probe	Advanced Cell Diagnostics	ACD 402811	
RNAscope mouse *Gli1 in situ* probe	Advanced Cell Diagnostics	ACD 311001	
RNAscope mouse *Gli2 in situ* probe	Advanced Cell Diagnostics	ACD 405771	
RNAscope mouse *Bmp4 in situ* probe	Advanced Cell Diagnostics	ACD 401301	
RNAscope mouse *Noggin in situ* probe	Advanced Cell Diagnostics	ACD 467391	
**Experimental Models: Cell Lines**	**Source**	**Catalogue No.**	
hTERT-RPE1	ATCC	CRL-4000	
IMCD-3	ATCC	CRL-2123	
NIH-3T3	ATCC	CRL-1658	
HEK-293	ATCC	CRL1573	
Human dermal fibroblasts	Lonza	CC-2509	
**Experimental Models: Organisms/Strains**	**Source**	**Catalogue No.**	
Mouse: C57BL6/J wild type	Jackson Laboratories	000664	
Mouse: *Ptch1^{tm1Mps}/J*	Jackson Laboratories	003081	
Mouse: B6.Cg-*Shh$^{tm(EGFP/Cre)Cjt}$/J*	Jackson Laboratories	005622	
Mouse: *Adamts9^{Gt}*	Apte laboratory	N/A	
Mouse: *Adamts9^{Del}*	Apte laboratory	N/A	
Mouse: *Adamts20^{Bt}*	Rao C et al., *Development* (2003)	N/A	
Mouse: *Vcan^{Hdf}*	Yamamura H et al., *Dev Biol* (1997)	N/A	
**Oligonucleotides**	**Source**	**Catalogue No.**	
Silencer Select Clathrin heavy chain siRNA sense 5'-GGGAATTCTTCGTACTCCATT-3'	Ambion	S477 Cat# 4390824	
Silencer Select LRP-1 siRNA sense 5'-GGACGGCATCCACAATGTGTT-3'	Ambion	106762 Cat# AM51331	
Silencer Select LRP-2 siRNA sense 5'-CTCTGGATATGATATCGAATT-3'	Ambion	143880 Cat# AM16708	
Silencer Select ADAMTS16 siRNA sense 5'-CCCGAGAACTTAATCGCTATT-3'	Ambion	S46855 Cat# 4392420	

Silencer Select negative control siRNA #2	Ambion	Cat# 4390846	
**Recombinant DNA**	**Source**	**Catalogue No.**	
ADAMTS9 N-L2 pCDNA3.1 Myc/His plasmid DNA	Apte laboratory	N/A	
ADAMTS9 full length pCDNA3.1 Myc/His plasmid DNA	Apte laboratory	N/A	
ADAMTS9 full length E/A pCDNA3.1 Myc/His plasmid DNA	Apte laboratory	N/A	
ADAMTS20 full length pCDNA3.1 Myc/His plasmid DNA	Apte laboratory	N/A	
ADAMTS20 full length E/A pCDNA3.1 Myc/His plasmid DNA	Apte laboratory	N/A	
ADAMTS9 N-TSR12 pCDNA3.1 Myc/His plasmid DNA	Apte laboratory	N/A	
ADAMTS1 pCDNA3.1 Myc/His plasmid DNA	Apte laboratory	N/A	
ADAMTS5 pCDNA3.1 Myc/His plasmid DNA	Apte laboratory	N/A	
TIMP3 (pSAmhT3) pEE12 plasmid DNA	Apte laboratory	N/A	
pCR-Blunt II-TOPO vector	Invitrogen	K2800-40	
**qRT-PCR primers**	**Source**	**Catalogue No.**	
Human *CP110*-F 5'-TGTCTCAAGCGGACTCACTCCA-3'	Invitrogen	N/A	
Human *CP110*-R 5'-CCAGAGGTAGAATGGTGCTTCG-3'	Invitrogen	N/A	
Human *EHD1*-F 5'-GCGTTTGGCAACGCTTTCCTCA-3'	Invitrogen	N/A	
Human *EHD1*-R 5'-ATCCGCTGCTTCTCTCCAGACA-3'	Invitrogen	N/A	
Human *EHD3*-F 5'-TGGAGAGCATCAGCGTCATCGA-3'	Invitrogen	N/A	
Human *EHD3*-R 5'-CGAAGAGCAGAATGATGCGGTC-3'	Invitrogen	N/A	
Human *SMO*-F 5'-TGCTCATCGTGGGAGGCTACTT-3'	Invitrogen	N/A	
Human *SMO*-R 5'-ATCTTGCTGGCAGCCTTCTCAC-3'	Invitrogen	N/A	
Human *GLI1*-F 5'-AGCCTTCAGCAATGCCAGTGAC-3'	Invitrogen	N/A	
Human *GLI1*-R 5'-GTCAGGACCATGCACTGTCTTG-3'	Invitrogen	N/A	
Human *GLI2*-F 5'-GTCAGAGCCATCAAGACCGAG-3'	Invitrogen	N/A	
Human *GLI2*-R 5'-GCATCTCCACGCCACTGTCATT-3'	Invitrogen	N/A	
Human *PTCH1*-F 5'-GCTGCACTACTTCAGAGACTG-3'	Invitrogen	N/A	
Human *PTCH1*-R 5'-CACCAGGAGTTTGTAGGCAAGG-3'	Invitrogen	N/A	
Human *VCAN* all isoforms-F 5'-TTCAACCTTAATAGTAACCCATGC-3'	Invitrogen	N/A	

Human *VCAN* all isoforms-R 5'-AAGGTAGGCTGACTTTTCCAGAG-3'	Invitrogen	N/A	
Human *VCAN* -V0-F 5'-CAGCAAGCACAAAATTTCACC-3'	Invitrogen	N/A	
Human *VCAN* -V1-F 5'-TCGTTTTGAGAACCAGACAGG-3'	Invitrogen	N/A	
Human *VCAN* -V0/V1-R 5'-CTCAAATCACTCATTCGACCTG-3'	Invitrogen	N/A	
Human *GAPDH*-F 5'-AGCCTCAAGATCATCAGCAATG-3'	Invitrogen	N/A	
Human *GAPDH*-R 5’-CTTCCACGATACCAAAGTTGTCAT-3’	Invitrogen	N/A	
Mouse *Vcan* V0/V1-F 5’-CCCTTTGAGCATAGCAGTAGTAGTCA-3’	Invitrogen	N/A	
Mouse *Vcan* V0/V1-R 5’- ATGAGTTCCATACCAGTACTAGCATCTC-3’	Invitrogen	N/A	
Mouse *Fn1*-F 5’-GTCTAGGCGAAGGCAATGG-3’	Invitrogen	N/A	
Mouse *Fn1*-R 5’-CCTATAGGATGTCCGGGTGT-3’	Invitrogen	N/A	
Mouse *Has2*-F 5’-GGTCCAAGTGCCTTACTGAAAC-3’	Invitrogen	N/A	
Mouse *Has2*-R 5’-TGTAGAGCCACTCTCGGAAGTA-3’	Invitrogen	N/A	
Mouse *Hprt*-F 5’-GATCCATTCCTATGACTGTAGAT-3’	Invitrogen	N/A	
Mouse *Hprt*-R 5’-AGATCATCTCCACCAATAACTT-3’	Invitrogen	N/A	
**Software and Algorithms**	**Source**	**Catalogue No.**	
Image J / Image J FIJI	NIH	N/A	
LI-COR Image Studio (Ver4.0)	Li-COR Biosciences	N/A	
Leica Application Suite X (LAS-X)	Leica Microsystems	N/A	
Huygens HyVolution-2 plug-in to LAS-X	Scientific volume Imaging	N/A	
NRecon v1.6	Skycan	N/A	

Supplementary Table 1: Antibodies and other key reagents
