# Supplementary material for: Secreted metalloproteases ADAMTS9 and ADAMTS20 have a non-canonical role in ciliary vesicle growth during ciliogenesis
Source: Nat Commun. 2019 Feb 27;10:953. doi: 10.1038/s41467-019-08520-7 (PMC6393521; doi:10.1038/s41467-019-08520-7)
Supplement: Supplementary file 3 — Description of Additional Supplementary Files [file 41467_2019_8520_MOESM3_ESM.pdf]

Description of Additional Supplementary Files

File Name: Supplementary Movie 1
Description: Related to Figure 5: E13.5 wild type embryo imaged by optical projection tomography (OPT).

File Name: Supplementary Movie 2
Description: Related to Figure 5: E13.5 *Adamts9*$^{Gt/Gt}$*; Adamts20*$^{Bt/Bt}$ embryo imaged by OPT shows open neural tube, asymmetric body axis, craniofacial and limb anomalies, and organ heterotaxy.

File Name: Supplementary Movie 3
Description: Related to Figure 5: Coronal optical sections of an E13.5 wild type embryo imaged by OPT.

File Name: Supplementary Movie 4
Description: Related to Figure 5: Coronal optical sections of E13.5 *Adamts9*$^{Gt/Gt}$*; Adamts20*$^{Bt/Bt}$ embryo imaged by OPT shows heterotaxy of the visceral organs and asymmetric body axis.

File Name: Supplementary Movie 5
Description: Related to Figure 7: 3D-reconstruction of E9.5 wild type embryo section stained for chondroitin sulfate (anti-CS) shows specific staining in the neural tube floor plate.

File Name: Supplementary Movie 6
Description: Related to Figure 7: 3D-reconstruction of E9.5 *Adamts9*$^{Gt/Gt}$*; Adamts20*$^{Bt/Bt}$ embryo section stained with anti-CS shows massive CS accumulation throughout the neural tube.
