# Supplementary material for: Secreted metalloproteases ADAMTS9 and ADAMTS20 have a non-canonical role in ciliary vesicle growth during ciliogenesis
Source: Nat Commun. 2019 Feb 27;10:953. doi: 10.1038/s41467-019-08520-7 (PMC6393521; doi:10.1038/s41467-019-08520-7)
Supplement: Supplementary file 10 — Reporting Summary [file 41467_2019_8520_MOESM10_ESM.pdf]

nature research

Corresponding author(s): Suneel S. Apte

Reporting Summary

Nature Research wishes to improve the reproducibility of the work that we publish. This form provides structure for consistency and transparency in reporting. For further information on Nature Research policies, see Authors & Referees and the Editorial Policy Checklist.

Statistical parameters

When statistical analyses are reported, confirm that the following items are present in the relevant location (e.g. figure legend, table legend, main text, or Methods section).

n/a	Confirmed	
☐	☒	The exact sample size (*n*) for each experimental group/condition, given as a discrete number and unit of measurement
☐	☒	An indication of whether measurements were taken from distinct samples or whether the same sample was measured repeatedly
☐	☒	The statistical test(s) used AND whether they are one- or two-sided<br>*Only common tests should be described solely by name; describe more complex techniques in the Methods section.*
☒	☐	A description of all covariates tested
☒	☐	A description of any assumptions or corrections, such as tests of normality and adjustment for multiple comparisons
☐	☒	A full description of the statistics including central tendency (e.g. means) or other basic estimates (e.g. regression coefficient) AND variation (e.g. standard deviation) or associated estimates of uncertainty (e.g. confidence intervals)
☒	☐	For null hypothesis testing, the test statistic (e.g. *F*, *t*, *r*) with confidence intervals, effect sizes, degrees of freedom and *P* value noted<br>*Give P values as exact values whenever suitable.*
☒	☐	For Bayesian analysis, information on the choice of priors and Markov chain Monte Carlo settings
☒	☐	For hierarchical and complex designs, identification of the appropriate level for tests and full reporting of outcomes
☒	☐	Estimates of effect sizes (e.g. Cohen's *d*, Pearson's *r*), indicating how they were calculated
☐	☒	Clearly defined error bars<br>*State explicitly what error bars represent (e.g. SD, SE, CI)*

Our web collection on statistics for biologists may be useful.

Software and code

Policy information about availability of computer code

Data collection	Image J FIJI NIH<br>LI-COR Image Studio (Ver4.0) Li-COR Biosciences<br>Leica Application Suite X (LAS-X) Leica Microsystems<br>Huygens HyVolution-2 plug-in to LAS-X Scientific volume Imaging<br>NRecon v1.6 Skycan
Data analysis	Prism 7 for MacOS version 7.0.2 + Prism 5 for MacOS version 5.0

For manuscripts utilizing custom algorithms or software that are central to the research but not yet described in published literature, software must be made available to editors/reviewers upon request. We strongly encourage code deposition in a community repository (e.g. GitHub). See the Nature Research guidelines for submitting code & software for further information.

Data

Policy information about availability of data

All manuscripts must include a data availability statement. This statement should provide the following information, where applicable:

- Accession codes, unique identifiers, or web links for publicly available datasets
- A list of figures that have associated raw data
- A description of any restrictions on data availability

Provide your data availability statement here.

Field-specific reporting

Please select the best fit for your research. If you are not sure, read the appropriate sections before making your selection.

☒ Life sciences ☐ Behavioural & social sciences ☐ Ecological, evolutionary & environmental sciences

For a reference copy of the document with all sections, see nature.com/authors/policies/ReportingSummary-flat.pdf

Life sciences study design

All studies must disclose on these points even when the disclosure is negative.

Sample size	No sample size pre-calculation was made. The sample size varied depending on whether we were quantifying mouse phenotypes or cellular cilium measurements. For the former, it depended on the ease of getting the various combined genotypes, and for the latter it depended on the technical challenges of the imaging method. A minimum of 100 cells taken from 5 or more independent were used for conventional fluorescent microscopy. For electron microscopy, an N of 50 wild-type basal bodies and N of 100 mutant basal bodies were examined. For super-resolution imaging, the N varied from 8 micrographs for measurement of vesicle distance and size (Supplemental Fig. 2). For western blots, an N of 3 experiments was used. For SEM, n of 3 wild-type or mutant were used. These sample sizes proved to be sufficent for clearly discriminating the effects of ADAMTS9 mutation and pharmacological treatments (SAG, Dyngo4A).
Data exclusions	None
Replication	Biological replicates were used for mouse analysis as indicated in the text and sample size. For the cell biology, we used a single CRISPR-Cas9 clone D12, but essentially similar findings were present in the hemizygous RB4 clone. All experiments were replicated in technical and biological replicates as specified in figure legends.
Randomization	Not relevant to the present study
Blinding	Not relevant to the present study. Although initial phenotyping of mice was blinded, we had to obtain genotypes and match them to determine genotype-phenotype correlation

Reporting for specific materials, systems and methods

Materials & experimental systems

n/a	Involved in the study	
☐	☒	Unique biological materials
☐	☒	Antibodies
☐	☒	Eukaryotic cell lines
☒	☐	Palaeontology
☐	☒	Animals and other organisms
☒	☐	Human research participants

Methods

n/a	Involved in the study	
☒	☐	ChIP-seq
☒	☐	Flow cytometry
☒	☐	MRI-based neuroimaging

Unique biological materials

Policy information about availability of materials

Obtaining unique materials	No restrictions

Antibodies

Antibodies used	Described in Materials Table, uploaded as a supplemental file
Validation	Validation of new ADAMTS9 and ADAMTS20 antibodies by western blotting is described in Sup Fig 1; Only previously validated commercial antibodies were used. All antibodies to ADAMTS9 were validated by lack of staining in D12 cells.

Eukaryotic cell lines

Policy information about cell lines

Cell line source(s)	Please see Supplementary Table 1
Authentication	The cell lines were procured from ATCC and are thus authenticated
Mycoplasma contamination	All cell lines tested negative
Commonly misidentified lines (See ICLAC register)	None of the cell lines used appear in the ICLAC register

Animals and other organisms

Policy information about studies involving animals; ARRIVE guidelines recommended for reporting animal research

Laboratory animals	Mus musculus, C57BL/6, various ages and sex
Wild animals	None
Field-collected samples	None
