# Supplementary material for: Secreted metalloproteases ADAMTS9 and ADAMTS20 have a non-canonical role in ciliary vesicle growth during ciliogenesis
Source: Nat Commun. 2019 Feb 27;10:953. doi: 10.1038/s41467-019-08520-7 (PMC6393521; doi:10.1038/s41467-019-08520-7)
Supplement: Supplementary file 2 — Peer Review File [file 41467_2019_8520_MOESM2_ESM.pdf]

Reviewers' comments:

Reviewer #1 (Remarks to the Author):

The manuscript by Nandadasa et al, describes a role for the recycled secreted metalloproteases ADAMTS9 and ADAMTS20 in cilia assembly and function. Here, the authors reported that ADAMTS9 and ADAMTS20 function in ciliogenesis. They performed a detailed analysis of the ADAMTS9/ADAMTS20 double mutant mice and found impaired hedgehog signaling and abnormal ciliogenesis. Similarly, CRISPR was used to generate an ADAMTS9 null allele in cultured cells which showed ciliogenesis defects, and could be rescued by ADAMTS9 or ADAMTS20 expression. In the absence of ADAMTS9, ciliogenesis may be arrested at a stage where CP110 removal was blocked preventing mother centriole maturation. These proteins localized to the base of cilia on Rab11 positive endosomal recycling membrane vesicles. Further analysis revealed that ADAMTS9 and ADAMTS20 are recruited to this compartment in a Clathrin-dependent endocytic process following activation by furin at the surface of the cell. Overall, this work is of importance to the field because little is known about the early ciliogenesis regulation.

To strengthen arguments that ADAMTS's affect stages associated with membrane trafficking some additional analysis is important to include. It has been reported that early ciliogenesis stages including preciliary vesicle/distal appendage vesicle (DAV) stages can be identified from the CV stage. The authors appear to have combined these two discrete stages in Fig 4B. A CV is shown in bottom right and a DAV appears to be shown in upper right. Are these EM images representative of the percentage of structures observed? A clearer analysis of these structures could enable more precise function for the ADAMTS's in ciliogenesis processes. Given the importance of ADAMTS's in CP110 removal it would be advisable to quantify CP110 loss using imaging approaches as has been reported by many groups. The CV stage has also been linked to intraflagellar transport protein and transition zone protein recruitment. Did the authors examine these processes in knockdown cells? Furthermore, CP110 loss has been linked to another regulator of the endosome recycling compartment, EHD1. Were connections to this protein investigated? I also wonder if the deconvolution super resolution microscopy approach used in Figure 1 is able to accurately determine the size of individual vesicles, especially those that may be packed together in the pericentriolar area of cell If vesicles are closer together than the resolving power of this imaging approach you could be overestimating the size of these structures (296nm) compared to those further away from the basal body (190nm).

Additional concerns/questions/suggestions are listed below.

1. Shorter cilia were shown by SEM in fig.6C and 6D in the mutant mice. Did the authors look ciliogenesis in mutant MEFs to see if these was a stronger ciliogenesis effect as observed in RPE cells?
2. ADAMTS16 has been shown to regulate cilia length (Gopalakrishanan et al, 2012). Because ADAMTS9 and 16 seem have different substrates, ADAMTS16 should not be able to rescue ciliogenesis in ADAMTS9 KO RPE cells if the function of ADAMTS9 in ciliogenesis is direct. Did the authors try to rescue ciliogenesis with ADAMTS16 in ADAMTS9 KO RPE cells?
3. In Fig 3O, it is difficult to see the localization of Versican. Does it have ciliary base accumulation or ECM localization? Better to have a zoomed image.
4. It would be helpful to show parts of the basal body in Fig 1P as is shown in Fig 1Q.
5. In Fig. 4A, the Cep170 looks like a half ring in RB4 cells. Is this a representative image?
6. Reference 51 published in 2012 reviewing Rab11 endosomal ciliogenesis function is probably a bit dated and would not cover more recent observations about the contributions of other regulators of the endosome recycling compartment in ciliogenesis.
7. In the introduction the authors state that "transmembrane proteins such as smoothened are not targeted to the ciliary membrane by lateral transport through plasma membrane but via a complex mechanism utilizing cilia-bound recycling endocytic vesicles from the recycling endosome." However, Milenkovic L et al 2009 suggested that Smoothened can be transport to cilia through both lateral and vesicle pathways. I do not think the reference that was cited by the authors ruled out lateral transport

as a possible mechanism.

Reviewer #2 (Remarks to the Author):

The work by Nandasasa et al addresses for the first time the direct role of the proteases Adamts9 (and Adamts20) in ciliogenesis in vitro and in vivo mouse models. Recent reports have shown the complexity of cilia formation and functional screenings have identified components of the ciliosome whose absence results in congenital diseases (particularly heart congenital diseases; Li et al., 2015). While in these reports the endocytic machinery came up as relevant for ciliogenesis how vesicle traffic impacts on ciliogenesis is unclear. In this study, the authors demonstrate that in the absence of ADAMTS9 there is a defect in cilia (reduced numbers or shorter cilia in cells or mice, respectively) and the embryos lacking the protease show defects compatible with those reported in ciliopathies. It is also demonstrated that ADAMTS9 absence and thus altered cilia impairs the Hedgehog signaling pathway in the neural system and also in the yolk sac. However how ADAMTS9 is actually regulating cilia number and/or extension remains unclear and the authors present a collection of data in the cellular and mouse models difficult to reconcile. In addition, by employing state-of-the art super-resolution and electron microscopy, authors visualize ADAMTS9 at recycling Rab11+ vesicles around the basal body of the cilia and claim that ADAMTS9 at this subcellular location regulates cilia formation. The mechanism by which ADAMTS9 is sorted there and play a function to promote axoneme elongation is however incompletely defined since although the catalytic activity of Adamts9 is shown to be required it is not clear whether Adamts9 processes a non-identified intracellular substrate or by binding and processing proteoglycans (versican) it modulates indirectly cilia formation extracellularly. Distinguishing between these two possibilities would strengthen the conclusiveness and novelty of the study.
Although the reviewer appreciates elements of novelty that might be relevant for ciliogenesis and matrix proteases fields, particularly in the congenital disease context, and the experiments are overall well-performed and controlled, some of the conclusions regarding the role of recycled secreted Adamts9 in ciliogenesis need further experimental support or otherwise claims should be smoothened. In addition, in the reviewer's opinion the manuscript will benefit of re-writing and reordering some of the contents to make the message fully accessible.
Major concerns
1. The percentage of cilia in RPE-1 cells should be quantitated not only in clathrin-siRNA depleted cells but also in cells treated with the endocytosis inhibitors (Pistop-2 and Dingo4A) and in cells with LRP1/LRP2-siRNA deletion since in these cases only the impact on the presence of Adamts9 in vesicles is analyzed (Figures 2L and 2P). Indeed Figures 2L and 2O show cilia of apparent normal length in spite of the reduction/absence of Adamts9 at the recycling compartment arguing against a requirement for the protease at this location for ciliogenesis.
2. Since both siRNA_clathrin and siRNA-LRP1/LRP2 can affect endocytosis and recycling of many proteins present and involved in cilia formation (Li et al., 2015), authors should try to selectively block Adamts9 internalization/recycling by mutating the Gon-1 domain by which Adamts9 interacts with LRP1 and quantitate the presence of Adamts9 at recycling vesicles as well as the cilia phenotype. Biotin-labelling and Adamts9 pulse-chase experiments could also be considered to better establish the actual traffic route of secreted Adamts9 to the recycling compartment and cilium complex.
3. Figure 3G shows the peri-centriolar localization of the Adamts9-myc construct in D12 cells. Is the E/A mutant recycled/located nearby centriole/cilium base? This experiment will at least show whether indeed whether Adamts9 catalytic activity is required prior to/for internalization (as suggested by the conditioned medium rescue and the TIMP3 inhibition experiment?) or once in the recycling compartment to likely act intracellularly.
4. In Figure 3N, authors conclude that versican processing is not involved in the cilia defects observed in the absence of Adamts9 in cultured cells and that this defect is mediated by its presence in vesicles and its accumulation around the base of the cilium contributing to CP110 release, but there is no evidence for ADAMTS9 proteolytic action on CP110 releasing at this location. How are CP110 mRNA

levels in D12 cells? Is CP110 accumulation a primary or secondary event in cilia defects? Authors do not show co-localization of Adamts9 (or Rab11+) with CP110 in RPE-1 cells to show at least that they are at the same compartments.

5. Based on previous reports, authors claim that Shh pathway is altered in Adamts9 mutant mice downstream of the cilia defect. However, since Shh binding to HSPG shifts its dual developmental function towards proliferation (Chan et al. 2009; Vaillant & Monard, 2009)), it is still possible that accumulation of versican (or other related Adamts9 HSPG substrates) underlies the misbalanced Gli-A/Gli-R and enhanced proliferation observed in Adamts9 mutant embryos (Figure 6). Authors should test this possibility by analyzing if Shh pathway is also altered in D12 vs RPE-1 cells as in embryos and in such a case test whether soluble Shh could rescue the cilia phenotype. Accordingly, the contribution of unprocessed versican to the cilia phenotype in cells is not completely ruled out. Although mRNA levels are low in these cells, the authors should check the protein abundance/fragments and more importantly the Adamts9-generated neo-epitope by WB and/or IFI in the matrix deposited by RPE-1/D12 cells. Authors may also plate the cells on versican (or fibronectin) to check whether the cilia defect in Admats9 absence is exacerbated. Accumulated versican (or fibronectin) may directly affect apical-basal polarity and thus cilia formation by interacting with β1 integrin signals as for example recently shown for ECM inducing cilia growth via YAP to the nucleus (Xu et al., 2018; Kim et al., 2015). Is versican processing required for Adamts9 internalization (for example plating on mutated versican for the cleavage site or treating the cells with TIMP3 and checking Adamts9 at Rab11+ compartment) or the other way around, is Adamts9 presence at recycling Rab11+ vesicles required for versican processing by inhibiting endocytosis and checking for versican processing/accumulation? This will characterize this pathway more solidly even not ruling out additional intracellular actions to be explored in the future.

Minor concerns

1. Authors should clarify and discuss if the phenotype observed in the absence of Adamts9 mainly involves ciliogenesis (formation of cilia) or cilia extension and why since in vitro they quantitate percentage of cilia and is reduced but in vivo the number of cilia is normal but they are much shorter.
2. In the opinion of the reviewer, contents of current Figures 5 and 6 could be reordered: i) the phenotype of shorter cilia observed in Adamts9 mutant embryos; ii) their phenotypic features coincident with other ciliopathies; and iii) Shh aberrant signaling particularly in regions of the embryo where Adamts9 is expressed and its unprocessed substrates (versican and fibronectin) accumulated in the mutant mice.
3. In the images presented in Figures 1L-O, Adamts9 is in vesicles forming rosettes and with double membrane. Is it possible that Adamts9 is located in multi-vesicular bodies (MVB)/exosomes? Authors could comment about it since proteolytic activity driven by exosome traffic towards the apex of the cilium has been shown to be important for regulation of cilia in other models (Wood et al., 2013).
4. Several details should be revised in reference and numbering of Figures: Supp Fig. 1D is missing in the Figure but referred in the text (page 5); Fig. 3H-J (page 7) should better read Fig. 3H-K; in page 9 reference to Supp Fig. 4A is mistaken and it should be Supp Fig. 5A; in page 12 authors refer to Fig 4C and it should be Supp Fig. 7C. In the text reference to Figure panels is not always order (for example in page 7 Fig. 3L-M is referred in the text prior to Fig. 3H-K). In none of the legends authors mention de 'n' of individuals/experiments performed to generate the quantitative data and it is unclear how the number and length of cilia has been quantitated.
5. The reviewer suggests to revisit the title (to adjust it better to the presented data and conclusions; for example, ADAMTS9 localizes to the basal body and its catalytic activity is required for ciliogenesis), the Abstract (highlighting the key novelties and conclusions of the study), and the introduction providing basic concepts on primary cilia (definition, ciliogenesis, components, signaling and ciliopathies or defining 'gatekeeping transition zone') to facilitate further comprehension of the data and their relevance. There is also no mention to the Hedgehog pathway in the introduction and smoothening some statements as the las sentence is recommended since non-extracellular actions have been described for other matrix proteases (Shimizu-Hirota et al., 2012; Marchant et al., 2014). Conclusions at the end of every Result section often relied on 'suggest'. Authors should be more assertive on their conclusions from the data.

6. Working model shown in Figure 8 about Adamts9 traffic route and its contribution to the cilia phenotype have many open questions and should be refined.

Reviewer #3 (Remarks to the Author):

In the manuscript entitled, "Recycled secreted metalloproteases are essential for ciliogenesis", Nandadasa et al., provide evidence that the secreted proteases, ADAMTS9 and ADAMTS20 are enriched at the base of primary cilia, and that they are required for ciliogenesis in RPE1 cells, as well as in multiple tissues during mouse embryonic development. The authors further demonstrate a role for these proteases in HH signaling in multiple tissues, presumably through their effect on ciliogenesis. The authors also provide evidence that the loss of these proteases affects ECM composition in the developing neural tube. Overall, the results presented in this manuscript are novel and will be of high interest to multiple groups of researchers. Further, the experiments are largely well-controlled and of high quality. I have several comments and suggestions for the authors (detailed below) that I believe should be substantively addressed prior to further consideration of this manuscript. However, if addressed, then I would be supportive of publication of this manuscript. I believe this will be an important contribution to the fields of ciliogenesis, ADAMTS proteases, and HH signaling.

Major Comments:

1. I am a little confused by the data presented in Figure 6. The authors demonstrate what appears to be a slight reduction of Shh transcript in the rostral neural tube in Adamts9/20 mutants (6L), but a complete loss of SHH protein in both the notochord and floor plate (6H). Similarly contradictory data are presented for Ptch1 transcript and PTCH1 protein in Figures 6N and 6I. The authors need to address this discrepancy in their results. Along similar lines, it was surprising to me that Shh transcript was still detected in the floor plate, but OLIG2 and NKX6.1 expression were absent in the neural tube. Presumably, the higher levels of HH signaling required to induce SHH in the floor plate would be sufficient to induce both OLIG2 and NKX6.1. Here I would recommend that the authors expand their analysis to include both FOXA2 (a second FP marker) and NKX2.2 (another high level direct HH pathway target). There are good Abs for both these proteins (available from DSHB) that can be effectively used to further characterize this phenotype with little extra effort. The overall concern here is that if Shh is maintained in the notochord and floor plate, then how can the authors explain the loss of OLIG2 and NKX6.1? Conversely, if there is no SHH protein, then perhaps the loss of OLIG2 and NKX6.2 are due to a failure to initiate or maintain Shh expression in the notochord.

2. The increased cell proliferation observed in Adamts9/20 mutants in the neural tube is quite striking. I wonder if the authors have considered whether the increased cell proliferation is not a consequence of reduced ciliogenesis, but rather the cause of reduced ciliogenesis? Given that cells disassemble their cilia during mitosis, it seems plausible that cells undergoing such increased division would naturally have reduced ciliogenesis. I am not sure that I have a clean experiment to distinguish between these possibilities, but the author should at least address this possibility in their manuscript.

3. The preaxial polydactyly observed in Adamts9;Ptch1 compound mutants is quite interesting. However, it was not apparent whether there were any digit specification defects in Adamts9/20 mutants. The authors should explicitly address this in the text, and also comment on (or provide data for) Adamts9/20 expression in the limb mesenchyme.

4. In Figure 7, the authors provide evidence of increased HS abundance, while Perlecan levels are unchanged. This is a surprising result given that Perlecan is the major ECM HSPG; do the authors have evidence that cell surface HSPGs (e.g. Syndecans or Glypicans) have altered expression in their Adamts mutants? It is not clear to me how the authors can explain this finding

Minor Comments:

1. Using TIMP3 transfection to inhibit ciliogenesis in RPE-1 cells was a particularly interesting result. I wonder if the authors could perform a similar experiment in NIH/3T3 cells, which are HH-responsive? This would allow the authors to correlate TIMP3 transfection with reduced ciliogenesis, and presumably reduced HH pathway activity (as measured by qPCR for Gli1/Ptch1, or by a HH-responsive luciferase construct). While not an essential experiment, this could nicely corroborate their RPE-1 data, and also tie together the reduced HH signaling that they observe in the neural tube of the ADAMTS9/20 mutants.

2. Related to Figure 6, it would be useful to examine OLIG2 and NKX6.1 expression in more caudal sections of ADAMTS9/20 mutants to investigate whether there are any changes in neural patterning that might suggest non-cell autonomous effects of these secreted proteases at axial levels where at least Adamts9 is not expressed.

3. It is not clear to me why Supplemental Figure 7 is in the supplement. That is, I would recommend that the authors incorporate these data as a primary figure.

4. Can the authors utilize their ADAMTS9 and ADAMTS20 antibodies to characterize the in vivo localization of these proteins (relative to primary cilia) in either the developing neural tube, or the yolk sac?

We thank the reviewers for their constructive comments. In the point-by-point response below, the comments are in italics and our response is in plain text. We hope the reviewers will feel that the revisions adequately address their concerns.

Reviewer #1 (Remarks to the Author):

The manuscript by Nandadasa et al, describes a role for the recycled secreted metalloproteases ADAMTS9 and ADAMTS20 in cilia assembly and function. Here, the authors reported that ADAMTS9 and ADAMTS20 function in ciliogenesis. They performed a detailed analysis of the ADAMTS9/ADAMTS20 double mutant mice and found impaired hedgehog signaling and abnormal ciliogenesis. Similarly, CRISPR was used to generate an ADAMTS9 null allele in cultured cells which showed ciliogenesis defects, and could be rescued by ADAMTS9 or ADAMTS20 expression. In the absence of ADAMTS9, ciliogenesis may be arrested at a stage where CP110 removal was blocked preventing mother centriole maturation. These proteins localized to the base of cilia on Rab11 positive endosomal recycling membrane vesicles. Further analysis revealed that ADAMTS9 and ADAMTS20 are recruited to this compartment in a Clathrin-dependent endocytic process following activation by furin at the surface of the cell. Overall, this work is of
importance to the field because little is known about the early ciliogenesis regulation.

To strengthen arguments that ADAMTS's affect stages associated with membrane trafficking some additional analysis is important to include. It has been reported that early ciliogenesis stages including preciliary vesicle/distal appendage vesicle (DAV) stages can be identified from the CV stage. The authors appear to have combined these two discrete stages in Fig 4B. A CV is shown in bottom right and a DAV appears to be shown in upper right. Are these EM images representative of the percentage of structures observed? A clearer analysis of these structures could enable more precise function for the ADAMTS's in ciliogenesis processes. Given the importance of ADAMTS's in CP110 removal it would be advisable to quantify CP110 loss using imaging approaches as has been reported by many groups.

As recommended, we performed a thorough TEM analysis to distinguish clearly between DAV and CV stages and have now relabeled the DAV and CV stages in the revised TEM figures. The mutant TEM images shown are representative of the CV and DAV stages identified and new data for their quantification represents the percentages observed for each stage in revised Fig 4b,c. It is now clear from these results that there is a significant impairment of vesicular growth and progression in the absence of ADAMTS9 (Fig. 4c). We thank the reviewer for suggesting further analysis of the TEM data, which has been insightful in determining the specific stage of ciliogenesis arrest.

The CV stage has also been linked to intraflagellar transport protein and transition zone protein recruitment. Did the authors examine these processes in knockdown cells?

To address these suggestions, we have added immunostaining data for the TZ marker TCTN2 (revised Fig. 4j). The majority of knock out cells do not show an axoneme associated with the basal body, but the ones that do show a short axoneme and TCTN2 localizing to the TZ. Relevant to this observation we have now also added cilium length quantification to all the relevant experiments through out the manuscript.

Furthermore, CP110loss has been linked to another regulator of the endosome recycling compartment, EHD1. Were connections to this protein investigated?

In addition to EHD1, we provide new data on EHD3 (Fig. 4f.g.h). Western blot did not show any differences of either EHD1 and or EHD3. We also performed qRT-PCR for CP110, EHD1 and EHD3. Interestingly, the CP110 transcript level was significantly lower in the mutant cells, despite its persistence in the mutant maternal centrosome/basal body. These data are shown in revised Fig. 4f,g.

I also wonder if the deconvolution super resolution microscopy approach used in Figure 1 is able to accurately determine the size of individual vesicles, especially those that may be packed together in the pericentriolar area of cell If vesicles are closer together than the resolving power of this imaging approach you could be overestimating the size of these structures (296nm) compared to those further away from the basal body (190nm).

According to the manufacturer (Leica Microsystems) the Leica SP8 Hyvolution super-resolution microscopy can capture intracellular details down to 140nm. We believe that the pericentriolar vesicles appear larger not due to limitation in microscopy resolving power but due to the nature of the Rab11+ vesicles which are prone to fusion, thus generating a large ciliary vesicle. The smaller vesicles we observe further away from the cilium are on average 190nm in diameter. We have modified the text to specify the resolution of the imaging used.

Additional concerns/questions/suggestions are listed below.

1. Shorter cilia were shown by SEM in fig.6C and 6D in the mutant mice. Did the authors look ciliogenesis in mutant MEFs to see if these was a stronger ciliogenesis effect as observed in RPE cells?

We have not obtained or analyzed MEFs from the double mutant mice. This is because ADAMTS9 and ADAMTS20 show very specific expression in tissues and the ADAMTS9 mutant allele used here is hypomorphic. Indeed, combination of this allele with an inactivated one shown in Fig. 6d and Sup 5i-l gave a more severe phenotype that demonstrates how much function is retained in the hypomorph. In contrast RPE-1 cells have a complete knockout of ADAMTS9 and do not express ADAMTS20 at all. In the context of the mutant mice, there is already considerable evidence for reduction of cilia and cilium length in the neural tube and yolk sac mesoderm.

2. ADAMTS16 has been shown to regulate cilia length (Gopalakrishanan et al, 2012). Because ADAMTS9 and 16 seem have different substrates, ADAMTS16 should not be able to rescue ciliogenesis in ADAMTS9 KO RPE cells if the function of ADAMTS9 in ciliogenesis is direct. Did the authors try to rescue ciliogenesis with ADAMTS16 in ADAMTS9 KO RPE cells?

We investigated ADAMTS16 both by transfecting *Adamts16* or by knocking down ADAMTS16 in wild type and ADAMTS9 KO RPE1 cells (we confirmed first that RPE-1 cells express *ADAMTS16* mRNA). We performed the knock down experiment since in the above-mentioned publication, it was loss of Adamts16 by targeted inactivation in rats, which increased cilia length, and therefore knock down would be the more relevant experiment. Neither over-expression of *Adamts16* nor depletion of ADAMTS16 rescued ciliogenesis in ADAMTS9 null RPE1 cells. Over expression of *Adamts16* in wild type RPE1 cells did significantly increase the percentage of ciliated cells. The new data is shown in revised Fig. 9l. ADAMTS16 is phylogenetically distinct from ADAMTS9 and ADAMTS20, and ADAMTS16 mutant rats did not have developmental defects, so this new finding was not surprising.

3. In Fig 3O, it is difficult to see the localization of Versican. Does it have ciliary base accumulation or ECM localization? Better to have a zoomed image.

Very little versican expression and staining is observed in wild type RPE-1 cells during a 2 day experimental culture period (Sup Fig. 7a,b). We therefore obtained additional immunostaining data from cells cultured for 5 days where accumulation of versican in KO cells was indeed observed (Sup Fig 7c). However, the ciliogenesis analysis in vitro was done 24 hours after serum starvation and after a total of 2 days in culture. Hence versican accumulation after 5 days may not be relevant. Furthermore, we overexpressed versican in RPE-1 cells and did not observe any effect on primary cilia. These new data are shown in revised Fig. 9e-h. A new panel, Fig. 9f, shows higher resolution imaging of versican after over-expression in RPE-1 cells.

4. It would be helpful to show parts of the basal body in Fig 1P as is shown in Fig 1Q.
We revised Fig. 1p to included images containing the basal body.

5. In Fig. 4A, the Cep170 looks like a half ring in RB4 cells. Is this a representative image?

The half-ring structure shown in Fig 4a of the initial submission was one staining pattern observed, and is not representative of all cells. We imaged more cells and replaced this image with another showing the expected ring structure. There was no difference in CEP170 distribution in Wt or mutant RPE1 cells.

6. Reference 51 published in 2012 reviewing Rab11 endosomal ciliogenesis function is probably a bit dated and would not cover more recent observations about the contributions of other regulators of the endosome recycling compartment in ciliogenesis.

We added new references to the text as suggested including a recent review by Wang and Dynlacht 2018 in Development.

7. In the introduction the authors state that "transmembrane proteins such as smoothened are not targeted to the ciliary membrane by lateral transport through plasma membrane but via a complex mechanism utilizing cilia-bound recycling endocytic vesicles from the recycling endosome." However, Milenkovic L et al 2009 suggested that Smoothened can be transport to cilia through both lateral and vesicle pathways. I do not think the reference that was cited by the authors ruled out lateral transport as a possible mechanism.

We thank the reviewer for pointing this out. The text has been revised accordingly and the reference suggested by the reviewer is included in the citation list.

Reviewer #2 (Remarks to the Author):

The work by Nandasasa et al addresses for the first time the direct role of the proteases Adamts9 (and Adamts20) in ciliogenesis in vitro and in vivo mouse models. Recent reports have shown the complexity of cilia formation and functional screenings have identified components of the ciliosome whose absence results in congenital diseases (particularly heart congenital diseases; Li et al., 2015). While in these reports the endocytic machinery came up as relevant for ciliogenesis how vesicle traffic impacts on ciliogenesis is unclear. In this study, the authors

demonstrate that in the absence of ADAMTS9 there is a defect in cilia (reduced numbers or shorter cilia in cells or mice, respectively) and the embryos lacking the protease show defects compatible with those reported in ciliopathies. It is also demonstrated that ADAMTS9 absence and thus altered cilia impairs the Hedgehog signaling pathway in the neural system and also in the yolk sac. However how ADAMTS9 is actually regulating cilia number and/or extension remains unclear and the authors present a collection of data in the cellular and mouse models difficult to reconcile. In addition, by employing state-of-the art super-resolution and electron microscopy, authors visualize ADAMTS9 at recycling Rab11+ vesicles around the basal body of the cilia and claim that ADAMTS9 at this subcellular location regulates cilia formation. The mechanism by which ADAMTS9 is sorted there and play a function to promote axoneme elongation is however incompletely defined since although the catalytic activity of Adamts9 is shown to be required it is not clear whether Adamts9 processes a non-identified intracellular substrate or by binding and processing proteoglycans (versican) it modulates indirectly cilia formation extracellularly. Distinguishing between these two possibilities would strengthen the conclusiveness and novelty of the study.
Although the reviewer appreciates elements of novelty that might be relevant for ciliogenesis and matrix proteases fields, particularly in the congenital disease context, and the experiments are overall well-performed and controlled, some of the conclusions regarding the role of recycled secreted Adamts9 in ciliogenesis need further experimental support or otherwise claims should be smoothened. In addition, in the reviewer's opinion the manuscript will benefit of re-writing and reordering some of the contents to make the message fully accessible.

Major concerns
1. The percentage of cilia in RPE-1 cells should be quantitated not only in clathrin-siRNA depleted cells but also in cells treated with the endocytosis inhibitors (Pistop-2 and Dingo4A) and in cells with LRP1/LRP2-siRNA deletion since in these cases only the impact on the presence of Adamts9 in vesicles is analyzed (Figures 2L and 2P).

We have now added quantification of cilium length to all the relevant experiments in addition to the measurement of percentage of ciliated cells throughout the manuscript. The cilium length quantifications for the specific experiments mentioned above are shown in revised Sup. Fig. 3a-e.

Indeed Figures 2L and 2O show cilia of apparent normal length in spite of the reduction/absence of Adamts9 at the recycling compartment arguing against a requirement for the protease at this location for ciliogenesis.

This experiment referred to was misinterpreted by the reviewer. To clarify how it was done, we added the endocytosis inhibitors 24 hrs after serum starvation (i.e., after the cells have made the cilium) to specifically inquire if ADAMTS9 was required for the maintenance of the cilium compared to its biogenesis. Endocytosis inhibitor-mediated removal of ADAMTS9 post ciliogenesis did not have an affect on the cilium but showed that endocytosis was necessary for ADAMTS9 localization at the base of the cilium. We have revised the text to better explain this experiment.

2. Since both siRNA_clathrin and siRNA-LRP1/LRP2 can affect endocytosis and recycling of many proteins present and involved in cilia formation (Li et al., 2015), authors should try to selectively block Adamts9 internalization/recycling by mutating the Gon-1 domain by which Adamts9 interacts with LRP1 and quantitate the presence of Adamts9 at recycling vesicles as well as the cilia phenotype. Biotin-labelling and Adamts9 pulse-chase experiments could also be

considered to better establish the actual traffic route of secreted Adamts9 to the recycling compartment and cilium complex.

Although the Gon-1 domain was used as bait in the yeast two-hybrid screen, it may not be the only interacting domain. For example, in a recently submitted manuscript, where we characterized ADAMTS9 interaction with fibronectin, another putative partner identified in the screen, we found multiple interacting domains of ADAMTS9. The revised manuscript includes data suggesting that the Gon-1 domain is not essential for endocytosis of ADAMTS9 since addition of conditioned medium containing ADAMTS9 N-Linker 2 domain (lacking the Gon-1 domain and the last 7 TSRs) rescues ciliogenesis and that this protein localizes at the base of the cilium (Fig. 3j-m).

3. Figure 3G shows the peri-centriolar localization of the Adamts9-myc construct in D12 cells. Is the E/A mutant recycled/located nearby centriole/cilium base? This experiment will at least show whether indeed whether Adamts9 catalytic activity is required prior to/for internalization (as suggested by the conditioned medium rescue and the TIMP3 inhibition experiment?) or once in the recycling compartment to likely act intracellularly.

We conducted this experiment and added the new images showing that the catalytically inactive mutant also localizes to the cilium base (Fig. 3h,i). Furthermore, we overexpressed Timp-3 and performed Rab11 and ADAMTS9 co-staining (Fig. 3p). Together, these new experiments showed that blocking catalytic activity did not affect ADAMTS9 localization at the cilium base.

4. In Figure 3N, authors conclude that versican processing is not involved in the cilia defects observed in the absence of Adamts9 in cultured cells and that this defect is mediated by its presence in vesicles and its accumulation around the base of the cilium contributing to CP110 release, but there is no evidence for ADAMTS9 proteolytic action on CP110 releasing at this location. How are CP110 mRNA levels in D12 cells? Is CP110 accumulation a primary or secondary event in cilia defects? Authors do not show co-localization of Adamts9 (or Rab11+) with CP110 in RPE-1 cells to show at least that they are at the same compartments.

ADAMTS9 (secreted/ intravesicular) and CP110 (cytosolic) are in different cellular compartments and so we do not believe that CP110 is an ADAMTS9 substrate. We therefore believe the increased CP110 protein content and the persistent cap in the mutant RPE-1 basal body is a secondary affect due to reduced CV formation and docking in the mutant RPE1 cells. We performed qRT-PCR for *CP110, EHD1, EHD3* and added the new data in revised Fig. 4f. Notably, despite increased protein levels in mutant cells, *CP110* mRNA was reduced, implying a feedback regulatory loop. As suggested by the reviewer we did super-resolution microscopy imaging of Rab11 and CP110 co-immunostained cells after serum starvation for 6 hours only (Fig. 4i). We observe Rab11 vesicles close to the CP110 signal of the basal body; however it is difficult to interpret if the contents of the Rab11 vesicles are exposed to the CP110 cap, although as stated at the start of this paragraph, this is unlikely. We have modified the text accordingly.

5. Based on previous reports, authors claim that Shh pathway is altered in Adamts9 mutant mice downstream of the cilia defect. However, since Shh binding to HSPG shifts its dual developmental function towards proliferation (Chan et al. 2009; Vaillant & Monard, 2009)), it is still possible that accumulation of versican (or other related Adamts9 HSPG substrates) underlies the misbalanced Gli-A/Gli-R and enhanced proliferation observed in Adamts9 mutant embryos (Figure 6). Authors should test this possibility by analyzing if Shh pathway is also

altered in D12 vs RPE-1 cells as in embryos and in such a case test whether soluble Shh could rescue the cilia phenotype.

As recommended, we analyzed Shh responsiveness in the KO RPE-1 cells by adding SAG to culture medium (revised Fig9a-d). Similar to the mutant embryos, the KO RPE1 cells showed significantly low levels of Shh signaling (revised Fig. 9a-d). Furthermore, treatment with SAG did not rescue cilium defects of the KO cells (revised Fig. 9b,c).

Accordingly, the contribution of unprocessed versican to the cilia phenotype in cells is not completely ruled out. Although mRNA levels are low in these cells, the authors should check the protein abundance/fragments and more importantly the Adamts9-generated neo-epitope by WB and/or IFI in the matrix deposited by RPE-1/D12 cells.

We added new versican and versican neo epitope staining data after prolonged (5 day) culture of RPE-1 cells (Sup. Fig. 7c).

The temporal characteristic of the observed versican change is inconsistent with the more rapid kinetics of ciliogenesis, and moreover, ciliogenesis was not impaired upon versican overexpression (Fig. 9e-h). We overexpressed versican-V1 in RPE-1 cells and co stained for versican and primary cilia. Increasing versican did not have an effect on cilium length or the percentage of ciliated cells (Fig. 9e-h). Therefore, we propose that ADAMTS9 acts from within the cell in ciliogenesis and not indirectly via versican processing in ECM.

Authors may also plate the cells on versican (or fibronectin) to check whether the cilia defect in Admats9 absence is exacerbated. Accumulated versican (or fibronectin) may directly affect apical-basal polarity and thus cilia formation by interacting with β1 integrin signals as for example recently shown for ECM inducing cilia growth via YAP to the nucleus (Xu et al., 2018; Kim et al., 2015).

In addition to versican overexpression, we plated wild type and KO RPE-1 cells on fibronectin as suggested by the reviewer. This did not have an effect on ciliogenesis in wild type or mutant cells, and specifically did not rescue loss of ciliogenesis of the KO cells (Fig. 9i,j; Sup Fig 7d). These data further support a cell-intrinsic role for ADAMTS9 in ciliogenesis.

Is versican processing required for Adamts9 internalization (for example plating on mutated versican for the cleavage site or treating the cells with TIMP3 and checking Adamts9 at Rab11+ compartment) or the other way around, is Adamts9 presence at recycling Rab11+ vesicles required for versican processing by inhibiting endocytosis and checking for versican processing/accumulation? This will characterize this pathway more solidly even not ruling out additional intracellular actions to be explored in the future.

We performed the TIMP3 over expression experiment suggested by the reviewer. Inhibition of ADAMTS9 catalytic activity by overexpression of TIMP3 did not affect ADAMTS9 localization in Rab11 vesicles (Fig.3p). We have separately generated a versican cleavage resistant mouse (unpublished) by mutating the known ADAMTS9 cleavage site of versican (E441-A442). The homozygous mice are developmentally normal other than having soft-tissue syndactyly (seen in a number of ADAMTS mutants, supporting the requirement for ADAMTS-mediated versican processing in interdigit web regression) and do not show any cilium/Shh related defects. Our interpretation is that versican cleavage and ciliogenesis are tindependent functions of ADAMTS9 and ADAMTS20.

Minor concerns
1. Authors should clarify and discuss if the phenotype observed in the absence of Adamts9 mainly involves ciliogenesis (formation of cilia) or cilia extension and why since in vitro they quantitate percentage of cilia and is reduced but in vivo the number of cilia is normal but they are much shorter.

We have modified the text better explaining these observations and also added cilium length quantification with the RPE-1 cell experiments. We believe the hypomorphism of the *Adamts9* mouse allele and variable expressivity in the mice, compared to the complete KO in clonally isolated RPE-1 cells is the main cause of variability in ciliogenesis in mouse tissues and differences with the knockout RPE-1 cells.

2. In the opinion of the reviewer, contents of current Figures 5 and 6 could be reordered: i) the phenotype of shorter cilia observed in Adamts9 mutant embryos; ii) their phenotypic features coincident with other ciliopathies; and iii) Shh aberrant signaling particularly in regions of the embryo where Adamts9 is expressed and its unprocessed substrates (versican and fibronectin) accumulated in the mutant mice.

We agree that this is a very good alternative to the current flow of the manuscript and we gave it serious consideration. However, we concluded that readers would better follow the direction of the work in the present format.

3. In the images presented in Figures 1L-O, Adamts9 is in vesicles forming rosettes and with double membrane. Is it possible that Adamts9 is located in multi-vesicular bodies (MVB)/exosomes? Authors could comment about it since proteolytic activity driven by exosome traffic towards the apex of the cilium has been shown to be important for regulation of cilia in other models (Wood et al., 2013).

Despite extensive analysis of ADAMTS9 distribution in ciliated cells, we have not seen its localization in the axoneme. Although we agree that localization in exosomes would be an attractive possibility, it is not supported by any of the present data.

4. Several details should be revised in reference and numbering of Figures: Supp Fig. 1D is missing in the Figure but referred in the text (page 5); Fig. 3H-J (page 7) should better read Fig. 3H-K; in page 9 reference to Supp Fig. 4A is mistaken and it should be Supp Fig. 5A; in page 12 authors refer to Fig 4C and it should be Supp Fig. 7C. In the text reference to Figure panels is not always order (for example in page 7 Fig. 3L-M is referred in the text prior to Fig. 3H-K). In none of the legends authors mention de 'n' of individuals/experiments performed to generate the quantitative data and it is unclear how the number and length of cilia has been quantitated.

We thank the reviewer for careful editing; we have fixed these errors and also added the "N" values for each quantitative experiment in the figure legends. We have also added information about cilium length and number data acquisition to the methods section.

5. The reviewer suggests to revisit the title (to adjust it better to the presented data and conclusions; for example, ADAMTS9 localizes to the basal body and its catalytic activity is required for ciliogenesis), the Abstract (highlighting the key novelties and conclusions of the study), and the introduction providing basic concepts on primary cilia (definition, ciliogenesis,

components, signaling and ciliopathies or defining 'gatekeeping transition zone') to facilitate further comprehension of the data and their relevance. There is also no mention to the Hedgehog pathway in the introduction and smoothening some statements as the last sentence is recommended since non-extracellular actions have been described for other matrix proteases (Shimizu-Hirota et al., 2012; Marchant et al., 2014). Conclusions at the end of every Result section often relied on 'suggest'. Authors should be more assertive on their conclusions from the data.

We thank the reviewer for these suggestions and have made the recommended changes to the text (title, abstract, introduction and discussion) to improve the clarity of the introduction and to render the discussion more assertive. We added the recommended citations, but clarified that although these are examples of secreted and cell surface proteases acting intracellularly, none are known to participate in organelle biogenesis.

6. Working model shown in Figure 8 about Adamts9 traffic route and its contribution to the cilia phenotype have many open questions and should be refined.

We refined the model in new Fig. 10A by adding a recycling endosome, marking the Rab11 vesicles emanating from it, and by dividing the figure clearly depicting the intracellular and extracellular functions.

Reviewer #3 (Remarks to the Author):

In the manuscript entitled, "Recycled secreted metalloproteases are essential for ciliogenesis", Nandadasa et al., provide evidence that the secreted proteases, ADAMTS9 and ADAMTS20 are enriched at the base of primary cilia, and that they are required for ciliogenesis in RPE1 cells, as well as in multiple tissues during mouse embryonic development. The authors further demonstrate a role for these proteases in HH signaling in multiple tissues, presumably through their effect on ciliogenesis. The authors also provide evidence that the loss of these proteases affects ECM composition in the developing neural tube. Overall, the results presented in this manuscript are novel and will be of high interest to multiple groups of researchers. Further, the experiments are largely well-controlled and of high quality. I have several comments and suggestions for the authors (detailed below) that I believe should be substantively addressed prior to further consideration of this manuscript.
However, if addressed, then I would be supportive of publication of this manuscript. I believe this will be an important contribution to the fields of ciliogenesis, ADAMTS proteases, and HH signaling.

Major Comments:

1. I am a little confused by the data presented in Figure 6. The authors demonstrate what appears to be a slight reduction of Shh transcript in the rostral neural tube in Adamts9/20 mutants (6L), but a complete loss of SHH protein in both the notochord and floor plate (6H). Similarly contradictory data are presented for Ptch1 transcript and PTCH1 protein in Figures 6N and 6I. The authors need to address this discrepancy in their results.

We performed new RNA in situ hybridization experiments using E9.5 samples embedded in a true transverse sectional plane. This provided paraffin sections going through the affected

neural tube area in the mutants (similar plane to the vibratome sections acquired by agarose embedding, used for the immunostaining experiments). We performed *Shh*, *Gli-1*, *Gli-2* and *Ptch-1* in-situs and added the new data to Fig. 6p. These show a comparable loss of Shh signaling to that observed by protein immunostaining.

Along similar lines, it was surprising to me that Shh transcript was still detected in the floor plate, but OLIG2 and NKX6.1 expression were absent in the neural tube. Presumably, the higher levels of HH signaling required to induce SHH in the floor plate would be sufficient to induce both OLIG2 and NKX6.1. Here I would recommend that the authors expand their analysis to include both FOXA2 (a second FP marker) and NKX2.2 (another high level direct HH pathway target). There are good Abs for both these proteins (available from DSHB) that can be effectively used to further characterize this phenotype with little extra effort. The overall concern here is that if Shh is maintained in the notochord and floor plate, then how can the authors explain the loss of OLIG2 and NKX6.1? Conversely, if there is no SHH protein, then perhaps the loss of OLIG2 and NKX6.2 are due to a failure to initiate or maintain Shh expression in the notochord.

We purchased these antibodies from DSHB and performed the experiments suggested by the reviewer (revised Fig.6L,M). Both FOXA2 and Nkx2.2 immunostaining showed reduced staining in the mutants. Our findings suggested that Shh is NOT maintained in the floor plate.

2. The increased cell proliferation observed in Adamts9/20 mutants in the neural tube is quite striking. I wonder if the authors have considered whether the increased cell proliferation is not a consequence of reduced ciliogenesis, but rather the cause of reduced ciliogenesis? Given that cells disassemble their cilia during mitosis, it seems plausible that cells undergoing such increased division would naturally have reduced ciliogenesis. I am not sure that I have a clean experiment to distinguish between these possibilities, but the author should at least address this possibility in their manuscript.

We believe the increased cell proliferation observed in the neural tube could be due to the loss of balance in cell signaling cascades and the massive upregulation of BMP signaling observed in the mutant neural tube. In the mutant mice, there is a visibly more dramatic effect on cilium length rather than the percentage of ciliated cells; that the observed cells in the neural epithelium are ciliated (albeit with short cilia) argues they are not in a proliferative state in the cell cycle. We measured cell proliferation in the KO RPE-1 cells (Fig.4k). Similar to the mouse neural tissue, non-serum starved (cycling) KO RPE-1 cells showed significantly more pHH3 positive cells. However, upon serum starvation for cilia induction, the KO cells did successfully exit from the cell cycle and show a minimal number of pHH3 positive cells similar to that seen in wild type cells. We have added this to the discussion and acknowledged the possibility suggested by the reviewer.

3. The preaxial polydactyly observed in Adamts9;Ptch1 compound mutants is quite interesting. However, it was not apparent whether there were any digit specification defects in Adamts9/20 mutants. The authors should explicitly address this in the text, and also comment on (or provide data for) Adamts9/20 expression in the limb mesenchyme.

We never observe polydactyly in the Adamts9/20 combined mutants and we modified the text as the reviewer suggested. Previous publications from the lab showed Adamts9 and Adamts20 playing important roles during web regression during limb development and each showed expression in the limb and inter digit mesenchyme. Conditional deletion of Adamts9 in the limbs

(using Prx-Cre; Dubail et al, Genesis 2014) did not affect patterning. We updated the text giving more emphasis to the publications showing expression of Adamts9 and Adamts20 in the limb (Dubail et al Genesis 2014, McCulloch et al., Dev Cell 2008, Jungers K et al., Gene Expr Patterns 2005).

4. In Figure 7, the authors provide evidence of increased HS abundance, while Perlecan levels are unchanged. This is a surprising result given that Perlecan is the major ECM HSPG; do the authors have evidence that cell surface HSPGs (e.g. Syndecans or Glypicans) have altered expression in their Adamts mutants? It is not clear to me how the authors can explain this finding.

The anti-HS and perlecan staining patterns even in the wild type embryos (Fig. 7e,g) are significantly different from one another, arguing against the interpretation of perlecan being the major HSPG of embryos. Perlecan staining is specific to the basement membrane while anti HS shows interstitial ECM staining in the neural tube floorplate, and the surrounding mesenchyme in addition to the basement membrane. As the reviewer pointed out we do not know the source of increased HS staining. Among candidate substrates that this finding points to are a large number of syndecans and glypicans as well as agrin. We do not pursue this possibility in the present manuscript, but agree it is worthy of follow-up.

Minor Comments:

1. Using TIMP3 transfection to inhibit ciliogenesis in RPE-1 cells was a particularly interesting result. I wonder if the authors could perform a similar experiment in NIH/3T3 cells, which are HH-responsive? This would allow the authors to correlate TIMP3 transfection with reduced ciliogenesis, and presumably reduced HH pathway activity (as measured by qPCR for Gli1/Ptch1, or by a HH-responsive luciferase construct). While not an essential experiment, this could nicely corroborate their RPE-1 data, and also tie together the reduced HH signaling that they observe in the neural tube of the ADAMTS9/20 mutants.

We performed a different but related experiment in relation to HH-responsiveness in the KO RPE-1 cells by treating the cells with SAG and analyzing ciliogenesis and HH responsive gene transcription. The new data are shown in Fig 9a-d.

2. Related to Figure 6, it would be useful to examine OLIG2 and NKX6.1 expression in more caudal sections of ADAMTS9/20 mutants to investigate whether there are any changes in neural patterning that might suggest non-cell autonomous effects of these secreted proteases at axial levels where at least Adamts9 is not expressed.

We added new Olig2 and Nkx6.1 immunostaining data of the caudal NT as suggested by the reviewer (revised Fig. 6n,o). The caudal neural tube did not show a defect in ventralization or Shh signaling.

3. It is not clear to me why Supplemental Figure 7 is in the supplement. That is, I would recommend that the authors incorporate these data as a primary figure.

As suggested we made it a main figure (new Figure 8).

4. Can the authors utilize their ADAMTS9 and ADAMTS20 antibodies to characterize the in vivo

localization of these proteins (relative to primary cilia) in either the developing neural tube, or the yolk sac?

We have not been successful at getting the Adamts9 and Adamts20 antibodies to work in vivo mouse tissues. Specifically, visualizing the staining at the base of the cilium at sufficiently high resolution has been challenging owing to high endogenous fluorescent signal.

REVIEWERS' COMMENTS:

Reviewer #1 (Remarks to the Author):

This revised work from Nandadasa et al has addressed many of my concerns. In particular the TEM, TCTN2 and CP110 analyses provide a more detailed explanation of ADAMTS9 function on ciliogenesis. This work should be published.

I am still not convinced by the explanation provided in the rebuttal about the vesicle size data in Figure 1j,k. I do not feel this is data is critical to the conclusions in the manuscript. Notably, the authors do not provide any evidence that ADAMTS9 is important for promoting vesicle fusion adjacent to the basal body that is specific to ciliogenesis. While the super resolution microscopy approach used has a lateral resolution of 140 nm the axial resolution was not given. It is well know that the MTOC region has a high density of Rab11 vesicles, which could make it challenging to identify individual vesicles using this imaging approach. I would suggest removing this data as there is the potential for an incorrect interpretation.

Other points:
1. Since EHD proteins were not affected in ADAMTS9 KO cells, this data could be moved to supplementary.
2. In figure legend, 4f is missing.
3. Adamts9bt/bt should be Adamts20bt/bt in line 231
4. It is not accurate to say Rab11 vesicles bound to the cp110 cap. It is more likely that these structures are docked to distal appendages near or close to the cp110 cap (line 576)

Reviewer #2 (Remarks to the Author):

The reviewer appreciates the effort of the authors to incorporate most of the requested experimental work as well as textual/graph clarifications and considers that this revised version satisfactorily addresses the key concerns raised by the original manuscript.

Reviewer #3 (Remarks to the Author):

In the revised manuscript from Nandadasa et al., the authors perform significant additional experiments that support the overall conclusions of their manuscript. I only have a couple of minor comments for the authors to consider:

1) In the revised Figure 6, panels i, l, and m all appear to have significantly reduced background signal in the mutant tissues compared to their WT counterparts. When making the claim that PTCH1 expression is reduced, and that FOXA2+ and NKX2.2+ neural progenitors are reduced, one would assume that the tissues were processed and imaged under identical conditions. However, the differences in background (e.g., NKX2.2 signal in the dorsal neural tube) raise this question.

2) In Figure 9D, it would be expected that Gli1 and Ptch1 levels would be reduced, since they are well-established transcriptional targets of the HH pathway. In some circumstances, Gli2 expression has also been shown to be regulated by HH signaling. However, the lower Smo expression data is confusing. Smo is not thought to be a HH-responsive gene, so its reduction is perplexing.

Response to reviewer's comments

The reviewer comments are in italics, and our response is in normal text. We hope the revised manuscript and clarifications provided below address the reviewer's comments adequately.

Reviewer #1:

This revised work from Nandadasa et al has addressed many of my concerns. In particular the TEM, TCTN2 and CP110 analyses provide a more detailed explanation of ADAMTS9 function on ciliogenesis. This work should be published.

I am still not convinced by the explanation provided in the rebuttal about the vesicle size data in Figure 1j,k. I do not feel this is data is critical to the conclusions in the manuscript. Notably, the authors do not provide any evidence that ADAMTS9 is important for promoting vesicle fusion adjacent to the basal body that is specific to ciliogenesis. While the super resolution microscopy approach used has a lateral resolution of 140 nm the axial resolution was not given. It is well know that the MTOC region has a high density of Rab11 vesicles, which could make it challenging to identify individual vesicles using this imaging approach. I would suggest removing this data as there is the potential for an incorrect interpretation.

Thank you for this comment. We feel the data is useful in that it indicates that the ADAMTS9 vesicles that are near the basal body are a distinct population from others further away. As Figure 1k shows, there is a marked size increase in the juxta-ciliary vesicles, that suggests vesicular growth, possibly by fusion. We discussed the resolution metrics with Technical Support at Leica and they informed us that the axial resolution was comparable to the lateral resolution, although it was dependent on the optical section thickness and wavelength of the channel used. We note that in other images, which are single optical sections, such as Figure 1l-n, and Figure 4a, the resolution is sufficient to discriminate between CEP170 and CEP164, which occupy proximate but distinct cell compartments. The data is also relevant to Figure 1 o, which uses electron microscopy to show the location of ADAMTS9; as indicated in the text, the results were consistent across techniques.

Other points:

1. Since EHD proteins were not affected in ADAMTS9 KO cells, this data could be moved to supplementary.

We retained the data in this location, since it was coupled to measurement of *CP110* mRNA.

2. In figure legend, 4f is missing.

We have revised the figure legend to include a description of 4f.

3. Adamts9bt/bt should be Adamts20bt/bt in line 231

Thank you. This has been corrected.

4. It is not accurate to say Rab11 vesicles bound to the cp110 cap. It is more likely that these structures are docked to distal appendages near or close to the cp110 cap (line 576)

We revised the text to state that the Rab11 vesicles are near the CP110- cap.

Reviewer #2:

The reviewer appreciates the effort of the authors to incorporate most of the requested experimental work as well as textual/graph clarifications and considers that this revised version satisfactorily addresses the key concerns raised by the original manuscript.

No response was needed.

Reviewer #3:

In the revised manuscript from Nandadasa et al., the authors perform significant additional experiments that support the overall conclusions of their manuscript. I only have a couple of minor comments for the authors to consider:

1) In the revised Figure 6, panels i, l, and m all appear to have significantly reduced background signal in the mutant tissues compared to their WT counterparts. When making the claim that PTCH1 expression is reduced, and that FOXA2+ and NKX2.2+ neural progenitors are reduced, one would assume that the tissues were processed and imaged under identical conditions. However, the differences in background (e.g., NKX2.2 signal in the dorsal neural tube) raise this question.
The tissues were indeed processed and imaged identically. We revised the panels l and m to include the DAPI stain, to provide a spatial context, but the lack of background is essential as seen experimentally. For the ptch1 staining introducing DAPI obscured the signal, so this image was left as is.
2) In Figure 9D, it would be expected that Gli1 and Ptch1 levels would be reduced, since they are well-established transcriptional targets of the HH pathway. In some circumstances, Gli2 expression has also been shown to be regulated by HH signaling. However, the lower Smo expression data is confusing. Smo is not thought to be a HH-responsive gene, so its reduction is perplexing.
We agree, and have revised the text to state that "although *Smo*, which is not a known Shh target, was also unexpectedly downregulated (Fig. 9d).
